# Direct long-read RNA sequencing identifies a subset of questionable exitrons likely arising from reverse transcription artifacts

**DOI:** 10.1186/s13059-021-02411-1

**Published:** 2021-06-28

**Authors:** Laura Schulz, Manuel Torres-Diz, Mariela Cortés-López, Katharina E. Hayer, Mukta Asnani, Sarah K. Tasian, Yoseph Barash, Elena Sotillo, Kathi Zarnack, Julian König, Andrei Thomas-Tikhonenko

**Affiliations:** 1 Institute of Molecular Biology (IMB), Ackermannweg 4, 55128 Mainz, Germany; 2 Division of Cancer Pathobiology, Children’s Hospital of Philadelphia, Philadelphia, PA 19104 USA; 3 The Bioinformatics Group, Children’s Hospital of Philadelphia, Philadelphia, PA 19104 USA; 4 Division of Oncology, Children’s Hospital of Philadelphia, Philadelphia, PA 19104 USA; 5 Department of Genetics, Perelman School of Medicine at the University of Pennsylvania, Philadelphia, PA 19104 USA; 6 Present address: Stanford Cancer Institute, 265 Campus Dr., Stanford, CA 94305 USA; 7 Buchmann Institute for Molecular Life Sciences (BMLS) and Faculty of Biological Sciences, Goethe University Frankfurt, Max-von-Laue-Str. 15, 60438 Frankfurt, Germany; 8 Department of Pathology & Laboratory Medicine, Perelman School of Medicine at the University of Pennsylvania, Philadelphia, PA 19104 USA

**Keywords:** Long-read sequencing, Oxford Nanopore Technologies, Alternative splicing, mRNA isoforms, Exitrons, Reverse transcription, CD19, Immunotherapy, Blinatumomab

## Abstract

Resistance to CD19-directed immunotherapies in lymphoblastic leukemia has been attributed, among other factors, to several aberrant *CD19* pre-mRNA splicing events, including recently reported excision of a cryptic intron embedded within *CD19* exon 2. While “exitrons” are known to exist in hundreds of human transcripts, we discovered, using reporter assays and direct long-read RNA sequencing (dRNA-seq), that the *CD19* exitron is an artifact of reverse transcription. Extending our analysis to publicly available datasets, we identified dozens of questionable exitrons, dubbed “falsitrons,” that appear only in cDNA-seq, but never in dRNA-seq. Our results highlight the importance of dRNA-seq for transcript isoform validation.

**Supplementary Information:**

The online version contains supplementary material available at 10.1186/s13059-021-02411-1.

## Background

Aberrant splicing plays an important role in therapeutic resistance either by generating protein isoforms resistant to treatment or by eliminating target proteins entirely. A prime example of this phenomenon is B cell acute lymphoblastic leukemia (B-ALL) acquiring resistance to chimeric antigen receptor-armed autologous T cells (CART-19), which are engineered to target the CD19 surface antigen of B cells [1]. We previously demonstrated that skipping of exon 2 of *CD19* pre-mRNA generates a protein variant inherently resistant to killing by CART-19 and mis-localized in the endoplasmic reticulum [2, 3]. Subsequently, we and others have shown that retention of the *CD19* intron 2 containing a premature termination codon contributes to CART-19 resistance as well [4, 5]. Of note, several publications reported that apparent removal of a cryptic intron fully embedded within *CD19* exon 2 generates a novel isoform in healthy individuals and B-ALL patients (termed Δex2part) [2, 6–8]. One study further suggested that this event could mediate resistance to blinatumomab, a CD19-CD3-bispecific T cell engager ([6]; commentary by [9]). The same publication hypothesized that excision of the embedded intron might be catalyzed by the IRE1 (ERN1) endoribonuclease, which is responsible for unconventional splicing of the *XBP1* transcript during the unfolded protein response [10].

Such “exitrons” are known to exist in hundreds of human transcripts and are thought to evolve from ancestral coding exons, often preserving the open reading frames [11]. Given the potential significance of the reported *CD19* exitron, we began to investigate its nature using long-read Oxford Nanopore Technologies (ONT) sequencing. Long-read applications allow sequencing of complete transcript isoforms and have re-shaped our understanding of the complexities of human transcriptomes [12–14]. Different ONT protocols are currently available. In cDNA-seq, reverse transcribed (and often PCR-amplified) cDNA molecules are sequenced, while in dRNA-seq, polyadenylated mRNA molecules themselves are passed through the pores and read [15]. Both protocols can capture full transcripts, including alternatively spliced isoforms. However, dRNA-seq typically yields fewer reads and thus is most commonly used for detecting RNA modifications, such as adenine methylation [16]. Our data presented here indicate that the use of this method also avoids mis-identification of questionable exitrons (dubbed “falsitrons”), including but not limited to the one in *CD19* exon 2.

## Results and discussion

**Fig. 1:**
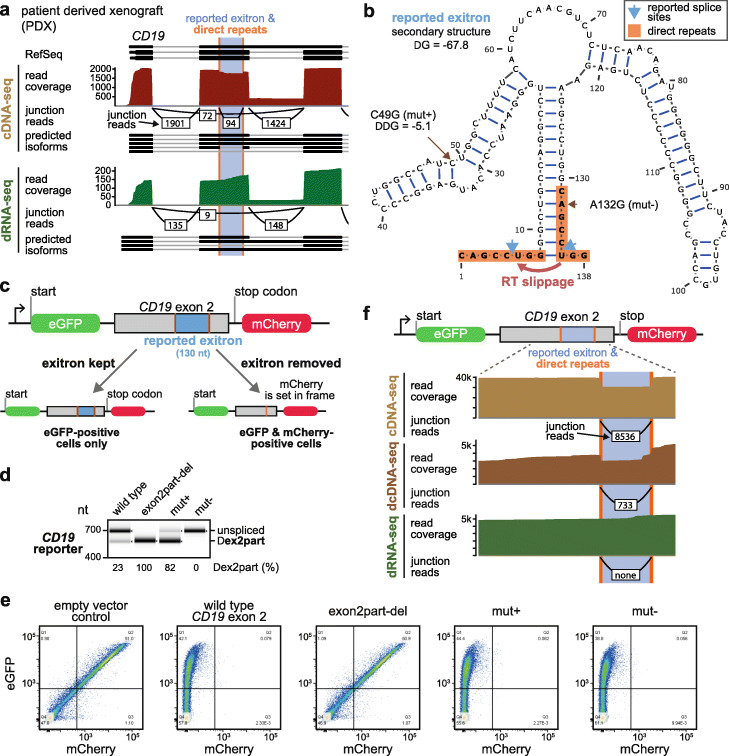
The reported exitron in the *CD19* exon 2 is a reverse transcription artifact. **a** Genome browser view showing cDNA-seq and dRNA-seq data for RNA from a patient-derived xenograft (PDX). Junction reads supporting the reported Δex2part product can be observed in cDNA-seq but are absent in the dRNA-seq. **b** Schematic of the predicted secondary structure and the direct repeats of the putative intron in *CD19* exon 2. **c** Schematic of the eGFP/mCherry-based reporter to detect splicing of the reported *CD19* exitron. **d** RT-PCR assay characterizing the *CD19* transcript isoforms for the wild type version and the variants of the reporter shown in panel c. They include two different point mutants predicted to stabilize the putative hairpin (mut+) or disrupt one of the direct repeats (mut−), as well as the control construct wherein the reported exitron has been deleted at the DNA level (exon2part-del). **e** Flow cytometry-based assay to characterize splicing of the reported exitron in HEK293T cells. **f** Genome browser view showing the region of *CD19* exon 2. cDNA-seq, dcDNA-seq, and dRNA-seq were performed on the same RNA sample from HEK293T cells expressing the mut+ reporter shown in panel c. Several hundred junction reads supporting exitron excision at the direct repeats in the cDNA-seq and dcDNA-seq data are detected, while none are found in the dRNA-seq

To investigate the processing of *CD19* exon 2, we treated the NALM-6 B-ALL cell line with thapsigargin, which induces unfolded protein response and IRE1 activity [10], and profiled select transcripts by RT-PCR. As anticipated, the levels of the spliced *XBP1* isoform were increased, but we did not detect changes in the reported *CD19* Δex2part product (Additional File 1: Fig. S1a). This called into question the role of IRE1 in exon 2 processing. We therefore decided to investigate aberrant splicing of *CD19* mRNA in B-ALL in more detail. To this end, we performed dRNA-seq and cDNA-seq on the same RNA sample from a therapy-resistant patient-derived xenograft [17] using long-read ONT sequencing. Both datasets documented the occurrence of several previously reported pathological *CD19* isoforms, including exon 2 skipping [2] and intron 2 retention [4]. Surprisingly, we failed to detect the Δex2part product in dRNA-seq, even though it was clearly observed in cDNA-seq (Fig. 1a). This suggested that it may be an artifact of the reverse transcription (RT)/PCR amplification-based protocol. Close examination of the *CD19* exon 2 sequence revealed that the putative exitron could be folding into a stable hairpin flanked by two 8-nt direct repeats (Fig. 1b), hinting at possible RT or PCR slippage at the base of the hairpin and ensuing product truncation.

To test this hypothesis, we engineered a dual-fluorescence GFP/RFP reporter (Fig. 1c) that would allow detection of *CD19* exitron excision by standard RT-PCR, and the corresponding protein product - via restoring the RFP open reading frame detectable by flow cytometry. Consistent with the *CD19* exitron excision being an RT-PCR artifact, we readily observed the corresponding RT-PCR product, but no RFP/GFP double-positive cells upon transfection into HEK293T cells (Fig. 1d, e). In addition, we introduced point mutations that were predicted to either increase the stability of the secondary structure (mut+; ΔΔG = − 5.1 kcal/mol) or disrupt one of the direct repeats (mut−; Fig. 1b). Consistent with our hairpin hypothesis, these reporter variants altered the levels of the Δex2part product in the RT-PCR-based assay. Namely, they were 82% higher in the case of mut+ or completely abolished in the case of mut− (Fig. 1d). Again, neither of them, not even mut+, yielded GFP/RFP double-positive cells (Fig. 1e). As a positive control, we removed the reported exitron from the reporter at the DNA level (exon2part-del) and readily observed both truncated RT-PCR product (Fig. 1d, e; Additional File 1: Fig. S1b, c) and robust expression of RFP (Fig. 1e).

To differentiate between RT and PCR artifacts, we performed dRNA-seq, direct cDNA (dcDNA)-seq omitting PCR amplification, and regular PCR-aided cDNA-seq on the reporter-transfected cells. To rule out the sensitivity issue, we used the mut+ reporter variant, which yields the highest levels of the Δex2part product in RT-PCR (Fig. 1e). Strikingly, in the long-read ONT data, the Δex2part product accounted for > 25% of dcDNA-seq and almost 30% of cDNA-seq reads, but was undetectable using dRNA-seq (Fig. 1f). This direct comparison of sequencing protocols indicated that excision of the reported *CD19* exitron occurs not in live cells, but in the test tube during the RT step, possibly due to the two direct repeats brought together at the base of the predicted hairpin structure. A similar phenomenon has been previously observed in the human *LIP1* and *FOXL2* genes [18, 19].

**Fig. 2:**
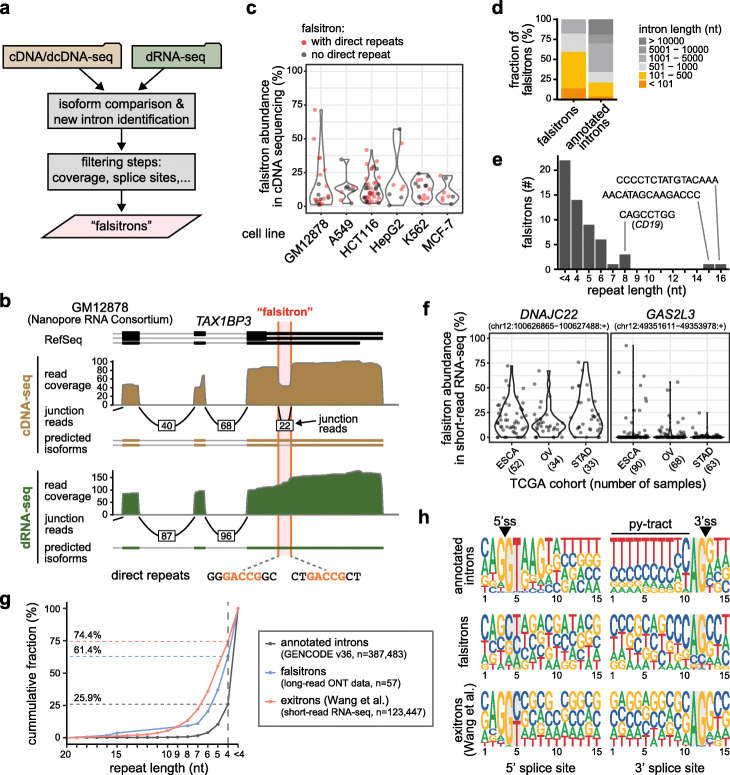
The detection of questionable exitrons is common in cDNA-seq and dcDNA-seq. **a** Schematic representation of the workflow to identify falsitrons in public ONT sequencing datasets. **b** Genome browser view showing the falsitron in *TAX1BP3* in ONT sequencing data for GM12878. **c** Violin plots indicating the detection of falsitrons in cDNA-seq and dcDNA-seq of different human cell lines. **d** Stacked bar plots showing the fraction of falsitrons of different lengths. **e** Bar graph depicting the length of falsitron-flanking direct repeats. **f** Violin plots show relative abundance of falsitron products in *DNAJC22* and *GAS2L3* for three TCGA cancer cohorts. ESCA, esophageal carcinoma. OV, ovarian serous cystadenocarcinoma. STAD, stomach adenocarcinoma. **g** Plot showing cumulative percentage with direct repeats of at least a given length. Dashed lines indicate the total fraction of introns with direct repeats (≥ 4 nt). **h** Sequence logos indicating nucleotide composition at 5′ and 3′ splice sites. Positions of splice site dinucleotide motifs are highlighted

Our results indicate that RT-based sequencing protocols can lead to the widespread mis-identification of exitrons. Indeed, the *CD19* exitron was recently reported to yield a new isoform in the long-read full-length cDNA-seq dataset obtained using the Rolling Circle Amplification to Concatemeric Consensus (R2C2) method serving to increase detection accuracy [7, 8]. To determine whether other transcripts are prone to such RT artifacts, we performed a targeted search in publicly available ONT sequencing datasets. Specifically, we screened for transcript isoforms that are present only in cDNA-seq but not in the matching dRNA-seq. This was achieved using several filtering steps, such as adjusting for read coverage and excluding the presence of canonical splice sites (Fig. 2a, Additional File 1: Fig. S2a, also see Methods). We first applied this comparison to cDNA-seq and dRNA-seq data for the B-lymphoblastoid cell line GM12878 from the Nanopore RNA Consortium [20]. We readily rediscovered the *CD19* exitron along with 19 other questionable exitrons, which we dubbed “falsitrons” (Fig. 2b, c, Additional File 1: Fig. S2b, Additional File 2: Data 1, Additional File 3: Table S1), supporting the common nature of such artifacts. We then extended our search to ONT sequencing data for five commonly used cell lines from the Singapore Nanopore Expression Project (SG-NEx) [21]: A549, HCT116, HepG2, K562, and MCF-7. In total, we discovered 100 candidate events corresponding to 57 unique falsitrons in 43 genes, for which “spliced” reads were present in the cDNA-seq (up to 70% of reads) but completely absent in the matched dRNA-seq (Fig. 2c, Additional File 2: Data 1, Additional File 3: Table S1). Many of these falsitrons were short (median length 353 nt; Fig. 2d), with the “spliced” regions flanked by direct repeats (35 out of 57; Fig. 2c, e). This discovery strengthens our hypothesis that falsitrons in many instances arise from RT slippage. These artifacts are not restricted to ONT data, but occur in other long-read sequencing protocols such as Iso-Seq (Isoform Sequencing, PacBio) as well [13]. We detected 33 out of 57 falsitrons in the reconstructed isoforms from publicly available Iso-Seq data for several human RNA samples (Alzheimer brain, lymphoblastoid cell line COLO829BL, melanoma cell line COLO829T and Human Universal Reference RNA—see the “Methods” section and Additional File 1: Fig. S2c).

Conceptually, such RT artifacts would not be restricted to long-read cDNA-seq data either and should also be found in conventional short-read RNA-seq protocols. To test this hypothesis, we screened the Cancer Genome Atlas (TCGA) database [22] and immediately found six of the falsitrons in several cancer types. Overall, the abundance of the corresponding isoforms was low (< 5%), but could rise up to > 90% for certain samples and tumor types (Fig. 2f). This is potentially important, because a recent paper reported more than 100,000 exitrons in the TCGA database and suggested that the corresponding isoforms are novel cancer drivers and neoepitopes [23]. To learn whether such analyses might be affected by RT artifacts, we overlaid the falsitrons from our ONT data comparison onto these reported exitrons. We found that five falsitrons, including the *CD19* one, overlapped with reported exitrons. To our surprise, we further detected direct repeats (≥ 4 nt) overlapping the putative splice sites in almost 75% of the reported exitrons (91,852 out of 123,337; median length 5 nt), i.e. even more than in our falsitron list (with the shorter median length of 4 nt; Fig. 2g). In contrast, only ~ 25% of all annotated introns harbored such direct repeats at their splice sites (median length < 4 nt). Moreover, even though exitrons had been selected for canonical splice site dinucleotides (GU/GC-AG), they lacked other characteristics of 5′ and 3′ splice sites such as U1 complementarity and the polypyrimidine tract (Fig. 2h). This finding indicates that a significant fraction of the reported exitrons could also be RT artifacts. Although this observation awaits experimental validation, it suggests that caution is required when interpreting RNA-seq mapping data. We envision that as more dRNA-seq data become available, the unequivocal classification of cryptic introns as exitrons or falsitrons will be possible.

## Conclusions

Here, we show that RT artifacts can lead to the detection of questionable exitrons (“falsitrons”) and non-existing transcript isoforms. Such artifacts are not limited to one study and occur reproducibly in all protocols which rely on RT, including standard RT-PCR and short-read RNA-seq, but also in ONT-based sequencing of cDNA (PCR-amplified or not). For laboratories looking to validate specific exitrons, utilization of thermo-stable reverse transcriptases (as in TGIRT-Seq [24]) and Northern blotting can be used to avoid artifacts, especially when exitrons in question are reasonably long. Moreover, at least one computational tool (SQANTI) has been developed to flag suspicious introns by implementing a machine learning classifier based on a variety of transcript descriptors [25]. For example, in the publicly available Iso-Seq dataset (PacBio) from the lymphoblastoid cell line COLO829BL derived from a melanoma patient [26], SQANTI2 correctly filters out the *CD19* falsitron (Additional File 1: Fig. S2c). However, such flagging could come at the expense of filtering out real exitrons. Thus, in our opinion, dRNA-seq should be utilized beyond RNA modification detection as a reliable validation tool for high-throughput transcriptome analysis. While it requires significant amount of input RNA and typically yield fewer reads, it does not pick up falsitrons and allows for a more accurate cataloging of bona fide transcript isoforms. As our work illustrates, the accuracy is particularly important when putative isoforms have clinical correlates, such as resistance to life-saving immunotherapies.

## Methods

### Cell lines and patient-derived xenografts

HEK293T cells were obtained from DSMZ. They were cultured in DMEM (Life Technologies) with 10% fetal bovine serum (Life Technologies) and 1% l-glutamine (Life Technologies). NALM-6 cells were obtained from ATCC and cultured in RPMI medium with the same additives as for HEK293T cells. All cells were kept at 37 °C in a humidified incubator containing 5% CO_2_. They were routinely tested for mycoplasma infection. Viably-cryopreserved cells from a patient-derived xenograft model of human B-ALL harboring a TCF3-HLF fusion (ALL1807) were established as previously described [17] and used for downstream sequencing studies.

### Cloning

The backbone of the splicing reporter (including both fluorophores) was generously provided by Ramanujan S. Hegde (MRC Laboratory of Molecular Biology, Cambridge, UK) [27]. We introduced exon 2 and part of exon 3 of the human *CD19* gene between GFP and mCherry. To this end, we amplified the *CD19* exon 2 insert sequence from human genomic DNA (Promega) with the following primers:

5′-GATGACGATGACAAGGCCGGATCTGGAGATAACGCTGTGCTGCA-3′ and 5′-GCCAACTTTGAGCCCAGGTGAATCGGTCCGAAACATTCCACCGGAACAGCTCCCCGCTGCCCTCCACATTGACT-3′. The backbone was amplified with the following primers 5′-GATTCACCTGGGCTCAAAGT-3′ and 5′-AGATCCGGCCTTGTCATCGT-3′. The amplification products were combined using Gibson assembly ready-made master mix from IMB Protein Production Core Facility. The generation of point mutations in the splicing reporter was achieved with the Q5 Site-Directed Mutagenesis Kit (New England Biolabs) according to the manufacturer’s recommendations.

### Dual-fluorescence splicing reporter assay via flow cytometry

Overexpression of the reporter plasmid was performed using Lipofectamine 2000 (Life Technologies) according to the manufacturer’s recommendation. Samples were transfected with reporter plasmids 48 h prior to flow cytometric analysis. Cells were washed in DPBS and trypsinized. After centrifugation, cells were washed twice with Dulbecco’s phosphate-buffered saline (DPBS) and resuspended in FACS buffer (DPBS, 1% BSA and 2 mM EDTA). Experiments were performed on the LSRFortessa SORP (BD Biosciences) and analyzed via the FlowJo (v10) software (FlowJo, LLC).

### Thapsigargin assay

Thapsigargin (Biomol GmbH) was used after 24 h post-transfection at a concentration of 250 nM for 2, 6, and 24 h on NALM-6 cells. Afterwards, cells were harvested and washed twice in PBS. RNA was isolated with the RNeasy Plus Mini Kit (Qiagen).

### Quantification of splicing isoforms with RT-PCR

Semiquantitative RT-PCR was used to quantify ratios of *CD19* and *XBP1* mRNA isoforms. To this end, reverse transcription was performed on 500 ng RNA with RevertAid Reverse Transcriptase (Thermo Fisher Scientific) according to the manufacturer’s recommendations. Subsequently, 1 μl of the cDNA was used as template for the RT-PCR reaction with the OneTaq DNA Polymerase (New England Biolabs) (Cycler conditions: 94 °C for 30 s, 28 cycles [reporter PCR] or 34 cycles [endogenous *CD19*, *XBP1*] of [94 °C for 20 s, 53 °C [reporter assay] or 55 °C [*CD19* endogenous] or 54 °C [*XBP1*] for 30 s, 68 °C for 30 s] and final extension at 68 °C for 5 min). The primers 5′-CGCGATCACATGGTCCTTAA-3′ and 5′-CATGTTATCCTCCTCGCCCT-3′ were used for the reporter assay, 5′-ACCTCCTCGCCTCCTCTTCTTC-3′ and 5′-CCGAAACATTCCACCGGAACAGC-3′ for the endogenous PCR on *CD19* and 5′-CCTGGTTGCTGAAGAGGAGG-3′ and 5′-CCATGGGGAGATGTTCTGGAG-3′ for *XBP1*. The TapeStation 2200 capillary gel electrophoresis instrument (Agilent) was used for quantification of the PCR products on D1000 tapes.

### Nanopore sequencing

For the ONT sequencing of the PDX sample ALL1807 or HEK293T cells transfected with the mut+ reporter construct, total RNA was extracted using Trizol reagent following manufacturer’s recommendation. The mRNA was isolated from 100 μg of total RNA using Dynabeads mRNA DIRECT Kit (Invitrogen). The mRNA samples were subjected to PCR-cDNA (SQK-PCS109, ONT), direct-cDNA (SQK-DCS109, ONT) and direct-RNA (SQK-RNA002, ONT) library preparation in parallel using the equipment and consumables according to each library protocol. Subsequently, each library was loaded into a Spot-ON flow cell R9 Version (FLO-MIN106D, ONT) and sequenced on a MinION Mk1B device (ONT) for 48 h. The RNA from the sample ALL1807 was submitted to the Sequencing Technologies and Analysis Core at Cold Spring Harbor Laboratory for PCR-cDNA library preparation and sequencing on a PromethION device (ONT).

### Nanopore sequence analysis

Base calling was performed using the ONT data processing toolkit guppy (version 3.4.5). guppy_basecaller was run with default settings providing the specific flow cell and library preparation pairs. The resulting reads were aligned to either the human reference genome (version hg38) or our custom *CD19* reporter (mut+) sequence using minimap2 (version 2.17-r941) [28], using the following flags “-k 12 -u b -x splice --secondary=no”. For downstream transcriptome analysis, we used the ONT pipeline [github.com/nanoporetech/pipeline-nanopore-ref-isoforms], which implements pre-processing with pychopper (DNA only), mapping with minimap2 and transcriptome reconstruction with StringTie [29] in long-read mode. Finally, the annotation obtained from StringTie was compared back to the existing annotation using gffcompare [30]. This pipeline was modified to run StringTie without annotation to guide the reconstruction and we omitted the “--conservative” flag.

### ONT data comparison to identify falsitrons

In order to identify additional falsitrons, we compared cDNA-seq and dRNA-seq data produced by the Nanopore RNA Consortium [20] and the Singapore Nanopore Expression Project (SG-NEx) [21]. The first dataset from the Nanopore RNA Consortium contains dRNA-seq and cDNA-seq data for the cell line GM12878. SG-NEx offers cDNA-seq, dcDNA-seq, and dRNA-seq for the five commonly used cell lines A549, HCT116, HepG2, K562 and MCF-7. For each dataset, we used StringTie for isoform reconstruction as described above. For read filtering, we used the default parameters specified in the pipeline: --minimum_mapping_quality 40, --poly_context 24, and --max_poly_run 8. We then contrasted the GFF transcript output files from StringTie using gffcompare which provides a summary of all the distinct isoforms between two GFF files. We searched for falsitrons that are supported by “spliced” reads only in cDNA-seq but not in dRNA-seq. To do this, we inspected the pairs of “non-equal” isoforms for junction-spanning reads that were present only in cDNA-seq and were fully contained within an exon (filter 1a, Additional File 1: Fig. S2a) or had start and end coordinates that were resided in two adjacent exons detected in the dRNA-seq (filter 1b, Additional File 1: Fig. S2a). Based on the characteristics of *CD19* Δexon2part, we applied additional filters, i.e. a minimum coverage of five reads of both cDNA-seq and dRNA-seq (as reported by StringTie), and the lack of canonical GU-AG splice sites. Using these search criteria, we identified 100 candidate events arising from 57 unique putative falsitrons. Of those, 35 contained direct repeats in the splice sites ranging from 3 to 16 nt, similar to the 8-nt repeats in *CD19* Δex2part. Read numbers, mapping statistics, and gffcompare results for the samples are reported in Additional File 4: Table S2. Genome browser views showing ONT cDNA-seq and dRNA-seq data from all putative falsitron events are shown in Additional file 2: Data 1. The code for the falsitron search is available in Zenodo/Github under an open source MIT license [31, 32].

### Direct repeat search

For each candidate event, we searched for the presence of the same *k*-mers with length from 4 to 20 nt in a 40-nt window around each splice site. The *k*-mers were required to overlap at least 1 nt of the 5′ and 3′ dinucleotide motifs. The same analysis was applied to all the exitrons detected in Wang et al. [23] as well as for all unique annotated introns in GENCODE gene annotation (v36, genome version hg38) [33].

### Junction search in TCGA

We use the R/Bioconductor package snapcount [https://github.com/langmead-lab/snapcount] to query the 57 putative falsitrons from our ONT data comparison in short-read RNA-seq data from the Cancer Genome Atlas (TCGA) database. As most of the putative falsitrons end in repetitive regions, like in the case of *CD19* Δex2part, we allowed the splice sites to be shifted outwards by an offset of up to 1 repeat length of that given intron, as long as the resulting junction did not differ by more than ± 1 repeat length from the original junction length. Following these filters, we detected six of our putative falsitrons in TCGA. These reside in the following genes (genomic coordinates of falsitron in brackets): *PHAX* (chr5:126625543-126625746:+), *CCDC86* (chr11:60842626-60842700:+), *DNAJC22* (chr12:49351611-49353978:+), *GAS2L3* (chr12:100626865-100627488:+), *CDC27* (chr17:47118517-47118594:−), and *H1F0* (chr22:37807089-37807354:+).

### Relative isoform abundance estimates

For the long-read ONT data, relative isoform abundance was calculated by dividing the number of split reads supporting the falsitron junction over the total number of reads overlapping the junction coordinates. Operations were performed using the R/Bioconductor package GenomicAlignments [34]. For the TCGA data, we calculated relative isoform abundances by dividing the spliced reads (quantified using snapcount) over the mean of reads overlapping the junction region. The latter were quantified with data from the ReCount database [35] via the R/Bioconductor packages megadepth and recount3 [36].

### Nucleotide composition at splice sites

For the sequence logos at splice sites, we retrieved the sequence in a 15-nt window (3 nt in the exon + 12 nt in the intron) of the 3′ and 5′ splice sites of the different sets of introns: our putative falsitrons from the ONT comparison (*n* = 57), all unique exitrons reported by Wang et al. [23] (*n* = 123,337) and all unique introns in GENCODE gene annotation (v36, genome version hg38) (*n* = 387,483). We used the R package ggseqlogo [37] to plot the frequency of nucleotides in each set.

### Analysis of Iso-Seq data

Isoform predictions for Iso-Seq data (PacBio Sequel) before and after SQANTI2 filtering (v2.7) were taken from https://github.com/PacificBiosciences/DevNet/wiki/Melanoma%2D%2DCancer-Cell-Line-Iso-Seq-Data (for the lymphoblastoid cell line COLO829BL and melanoma COLO829T; PacBio Sequel), and https://downloads.pacbcloud.com/public/dataset/Alzheimer2019_IsoSeq/ (for total RNA from an Alzheimer’s Disease brain sample; PacBio Sequel II). The Universal Human Reference (Agilent; PacBio Sequel II) did not contain the SQANTI2 correction in the initial 2019 release (https://downloads-ap.pacbcloud.com/public/dataset/UHR_IsoSeq/). Upon request, we obtained a 2021 version of the annotation, filtered with SQANTI3 (https://downloads.pacbcloud.com/public/dataset/UHRRisoseq2021/). In the filtered files only 4 falsitrons were detected, located in the following genes: *DNAJC22* (chr5:126625543-126625746:+), *GAS2L3* (chr12:49351611-49353978:+), *CDC27* (chr12:100626865-100627488:+), *PHAX* (chr17:47118517-47118594:−).

## Supplementary Information

**Additional file 1: Figure S1.** Levels of the Δex2part product are not affected by thapsigargin treatment. a) RT-PCR experiments followed by capillary electrophoresis to quantify different *CD19* and *XBP1* isoforms. NALM-6 cells were treated with thapsigargin for indicated time intervals. b) RT-PCR experiments followed by capillary electrophoresis to quantify different *CD19* isoforms in HEK293T cells transfected with a mixture of mut- (A; does not produce Δex2partband) and exon2part-del (B; the reported intron is removed at the DNA level) reporter constructs. c) Flow cytometry-based assay performed on the same cells. **Figure S2.** The workflow to detect falsitrons captures the truncated *CD19* Δex2part product. a) Extended schematic representation of the workflow to identify questionable exitrons (dubbed “falsitrons”). b) Genome browser view depicting detection of the *CD19* falsitron (Δex2part) in ONT cDNA-seq, but not dRNA-seq data from the Nanopore RNA Consortium. c) Genome browser view shows that the *CD19* falsitron (Δex2part) is detected in PacBio Iso-Seq experiments but is filtered out when applying SQANTI2.

**Additional file 2: Data 1.** Putative falsitrons in the genomic context.

**Additional file 3: Table S1.** Putative falsitrons detected from Oxford Nanopore Technologies (ONT) sequencing data for the five commonly used cell lines A549, HCT116, HepG2, K562 and MCF-7, as well as the B-lymphoblastoid cell line GM12878.

**Additional file 4: Table S2.** Mapping and gffcompare statistics for Oxford Nanopore Technologies (ONT) sequencing datasets used in this study.

**Additional file 5.** Review history.

## Data Availability

The long-read ONT sequencing data for the PDX sample ALL1807 (cDNA-seq and dRNA-seq) and the HEK293T cells transfected with the mut+ reporter construct (cDNA-seq, dcDNA-seq and RNA-seq) are avaible in NCBI Short Read Archive under accession numbers SRR14326969-14326973 [38]: https://www.ncbi.nlm.nih.gov/sra/?term=SRR14326969 https://www.ncbi.nlm.nih.gov/sra/?term=SRR14326970 https://www.ncbi.nlm.nih.gov/sra/?term=SRR14326971 https://www.ncbi.nlm.nih.gov/sra/?term=SRR14326972 https://www.ncbi.nlm.nih.gov/sra/?term=SRR14326973 The computational code for the detection of falsitrons in ONT-Seq data is available in Zenodo/Github under an open source MIT license (10.5281/zenodo.4906610) [31, 32].
