# Supplementary material for: Direct long-read RNA sequencing identifies a subset of questionable exitrons likely arising from reverse transcription artifacts
Source: Genome Biol. 2021 Jun 28;22:190. doi: 10.1186/s13059-021-02411-1 (PMC8240250; doi:10.1186/s13059-021-02411-1)
Supplement: Supplementary file 1 — Additional file 1: Figure S1. Levels of the Δex2part product are not affected by thapsigargin treatment. a) RT-PCR experiments followed by capillary electrophoresis to quantify different CD19 and XBP1 isoforms. NALM-6 cells were treated with thapsigargin for indicated time intervals. b) RT-PCR experiments followed by capillary electrophoresis to quantify different CD19 isoforms in HEK293T cells transfected with a mixture of mut- (A; does not produce Δex2partband) and exon2part-del (B; the reported intron is removed at the DNA level) reporter constructs. c) Flow cytometry-based assay performed on the same cells. Figure S2. The workflow to detect falsitrons captures the truncated CD19 Δex2part product. a) Extended schematic representation of the workflow to identify questionable exitrons (dubbed “falsitrons”). b) Genome browser view depicting detection of the CD19 falsitron (Δex2part) in ONT cDNA-seq, but not dRNA-seq data from the Nanopore RNA Consortium. c) Genome browser view shows that the CD19 falsitron (Δex2part) is detected in PacBio Iso-Seq experiments but is filtered out when applying SQANTI2. [file 13059_2021_2411_MOESM1_ESM.pdf]

# Direct long-read RNA sequencing identifies a subset of questionable exitrons likely arising from reverse transcription artifacts

Laura Schulz[1*], Manuel Torres-Diz[2*], Mariela Cortés-López[1*], Katharina E. Hayer[3*], Mukta Asnani[2], Sarah K. Tasian[4], Yoseph Barash[5], Elena Sotillo[2&], Kathi Zarnack[6], Julian König[1#], and Andrei Thomas-Tikhonenko[2,7 #]

[1] Institute of Molecular Biology (IMB), Ackermannweg 4, 55128 Mainz, Germany. [2] Division of Cancer Pathobiology, Children's Hospital of Philadelphia, Philadelphia, PA 19104, US. [3] The Bioinformatics Group, Children's Hospital of Philadelphia, Philadelphia, PA 19104, US. [4] Division of Oncology, Children's Hospital of Philadelphia, Philadelphia, PA 19104, US. [5] Department of Genetics, Perelman School of Medicine at the University of Pennsylvania, Philadelphia, PA 19104, US. [6] Buchmann Institute for Molecular Life Sciences (BMLS), Goethe University Frankfurt, Max-von-Laue-Str. 15, 60438 Frankfurt, Germany. [7] Department of Pathology & Laboratory Medicine, Perelman School of Medicine at the University of Pennsylvania, Philadelphia, PA 19104, US.

* These authors contributed equally.

[&] Present address: Stanford Cancer Institute, 265 Campus Dr., Stanford, CA 94305

[#] Corresponding authors: Julian König (j.koenig@imb-mainz.de) and Andrei Thomas-Tikhonenko (andreit@pennmedicine.upenn.edu)

## SUPPLEMENTARY FIGURES

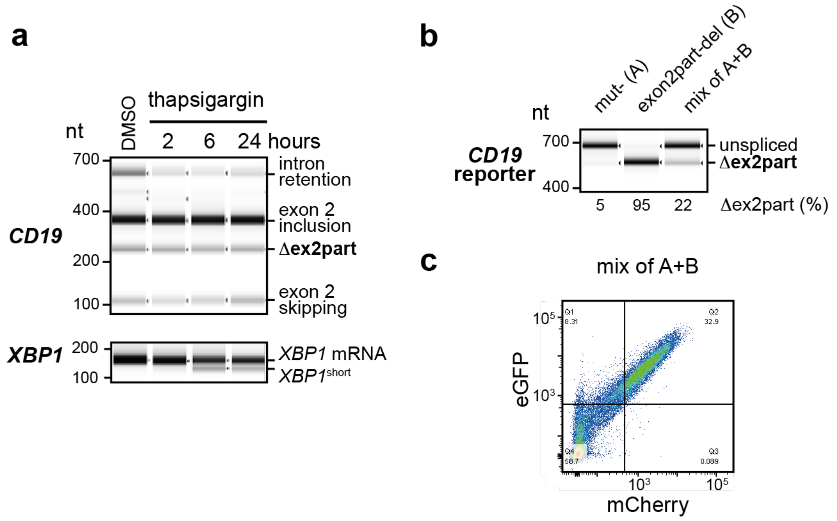


**Figure S1. Levels of the Δex2part product are not affected by thapsigargin treatment. a)** RT-PCR experiments followed by capillary electrophoresis to quantify different *CD19* and *XBP1* isoforms. NALM-6 cells were treated with thapsigargin for indicated time intervals. **b)** RT-PCR experiments followed by capillary electrophoresis to quantify different *CD19* isoforms in HEK293T cells transfected with a mixture of mut- (A; does not produce Δex2part band) and exon2part-del (B; the reported intron is removed at the DNA level) reporter constructs. **c)** Flow cytometry-based assay performed on the same cells.

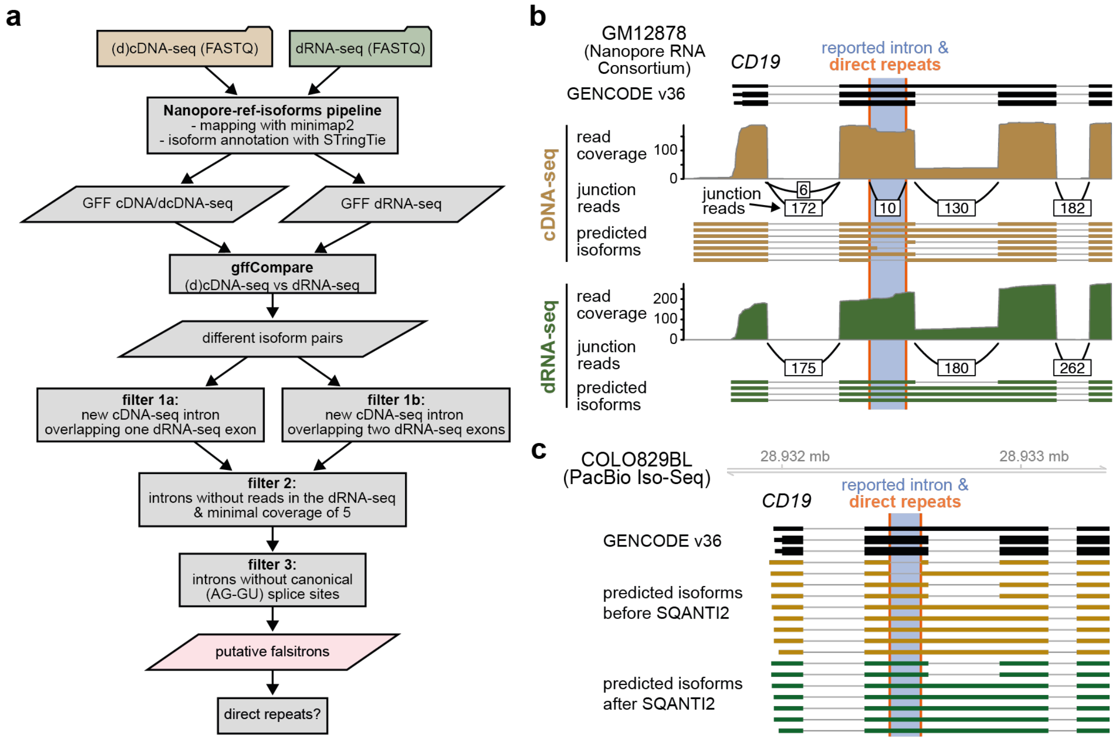


**Figure S2. The workflow to detect falsitrons captures the truncated *CD19* Δex2part product. a)** Extended schematic representation of the workflow to identify questionable exitrons (dubbed "falsitrons"). **b)** Genome browser view depicting detection of the *CD19* falsitron (Δex2part) in ONT cDNA-seq, but not dRNA-seq data from the Nanopore RNA Consortium. **c)** Genome browser view shows that the *CD19* falsitron (Δex2part) is detected in PacBio Iso-Seq experiments but is filtered out when applying SQANTI2.
