# Supplementary material for: Direct long-read RNA sequencing identifies a subset of questionable exitrons likely arising from reverse transcription artifacts
Source: Genome Biol. 2021 Jun 28;22:190. doi: 10.1186/s13059-021-02411-1 (PMC8240250; doi:10.1186/s13059-021-02411-1)

Data 1. Putative falsitrons in the genomic context. Genome browser view for 100 falsitron events that were detected in long-read ONT sequencing data produced by the Nanopore RNA Consortium and the Singapore Nanopore Expression Project (SG-NEx). For each event, the title provides the genomic coordinates (human genome version hg38), the gene symbol, the cell line in which the event was detected, and the direct repeat sequence if any. Each plot includes the following tracks: (i) reference transcript models (GENCODE v36), (ii) cDNA-seq read coverage (red), (iii) cDNA-seq sashimi plot of splice junctions, (iv) cDNA-seq transcript annotation (StringTie), (v) dRNA-seq read coverage (green), (vi) dRNA-seq sashimi plot of splice junctions, (vii) dRNA-seq transcript annotation (StringTie). The same tracks are also provided for dcDNA-seq (orange) if available. The position of the putative falsitron is highlighted in light red. For the sashimi plots, only junctions supported by a minimum of five reads are shown. Note that 11 events were detected in the comparison of both cDNA-seq and dcRNA-seq against dRNA-seq and are hence depicted on the same page.

chr1:28335161−28335708:+ MED18
Cell line: A549
Direct repeat sequence: CAGC

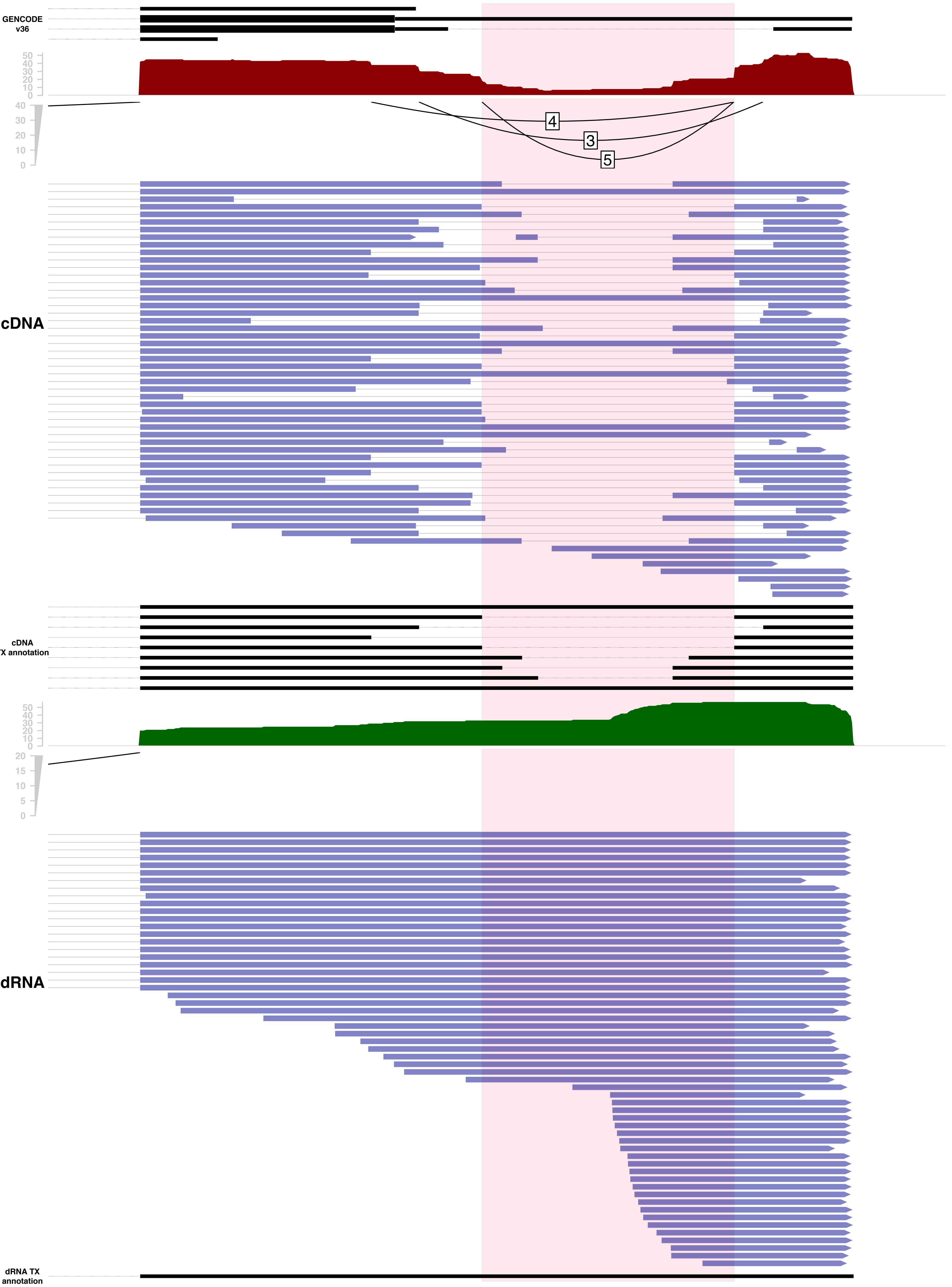

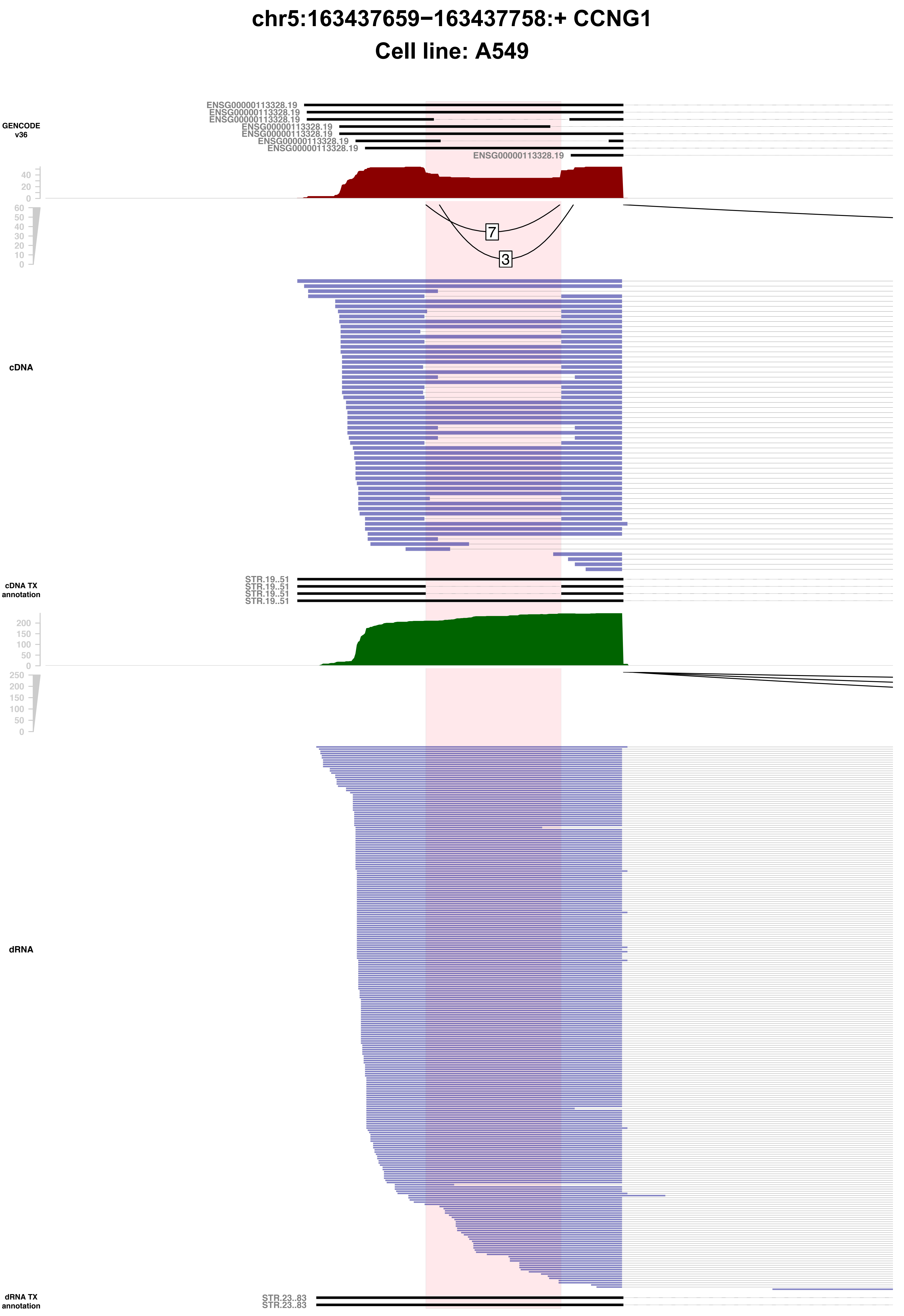
chr5:163437659−163437758:+ CCNG1
Cell line: A549
GENCODE v36
ENSG00000113328.19
cDNA
cDNA TX annotation
STR.19..51
dRNA
dRNA TX annotation
STR.23..83

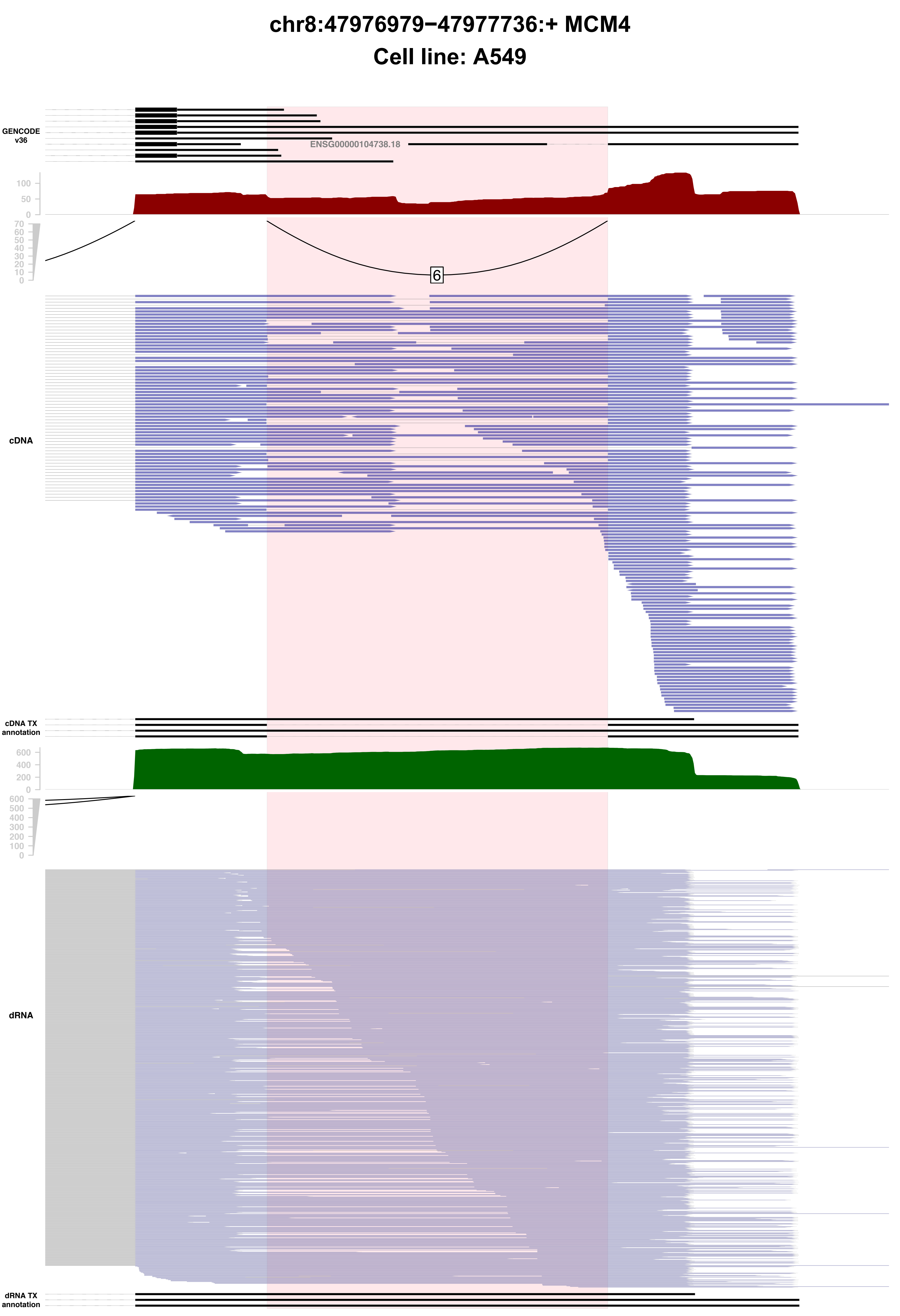

chr8:47976979−47977736:+ MCM4
Cell line: A549
GENCODE v36
ENSG00000104738.18
100
50
0
70
60
50
40
30
20
10
0
6
cDNA
cDNA TX annotation
600
400
200
0
600
500
400
300
200
100
0
dRNA
dRNA TX annotation

chr12:75501816−75501921:+ GLIPR1
Cell line: A549

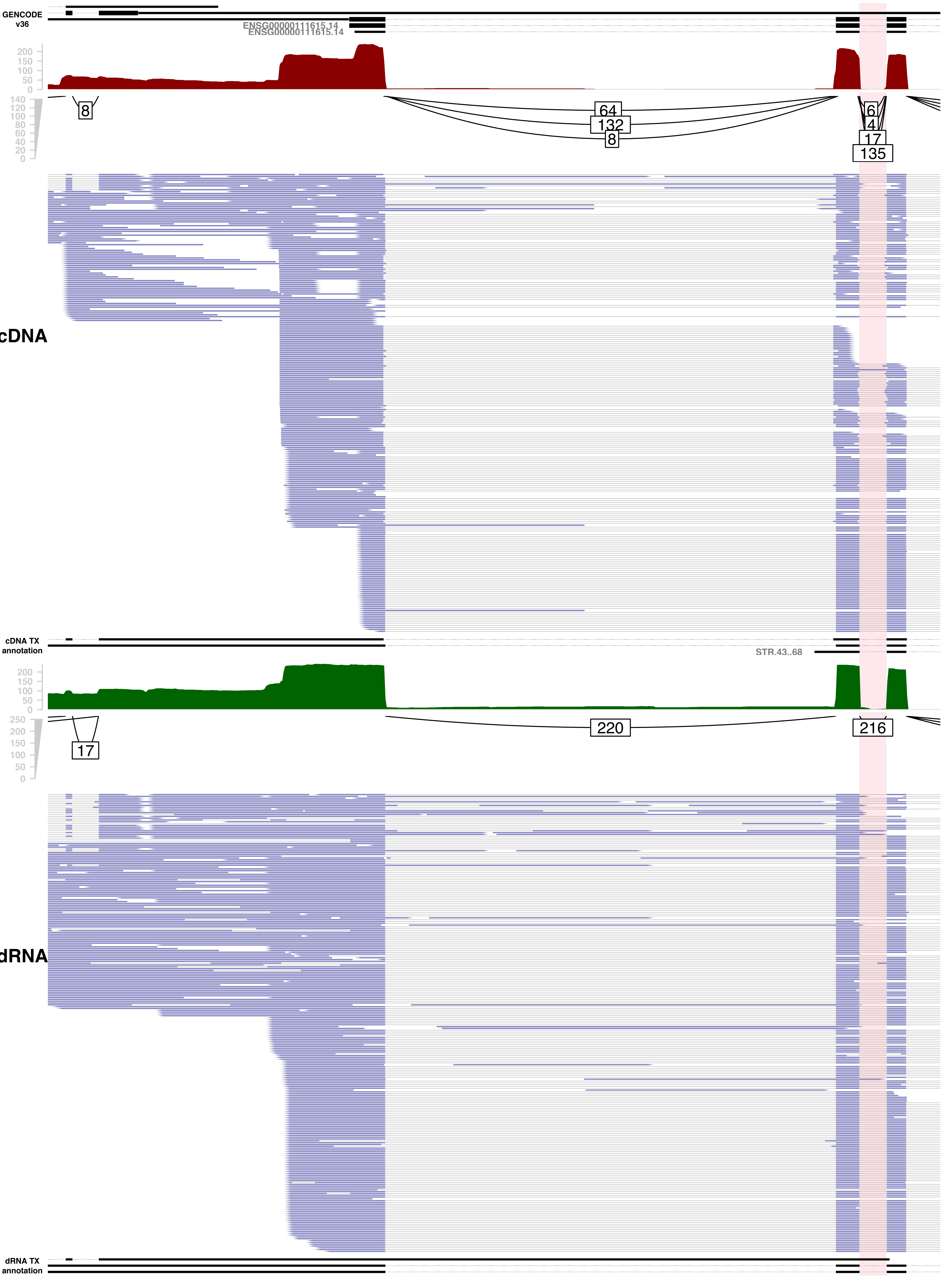

chr15:63121977−63122084:+ LACTB
Cell line: A549

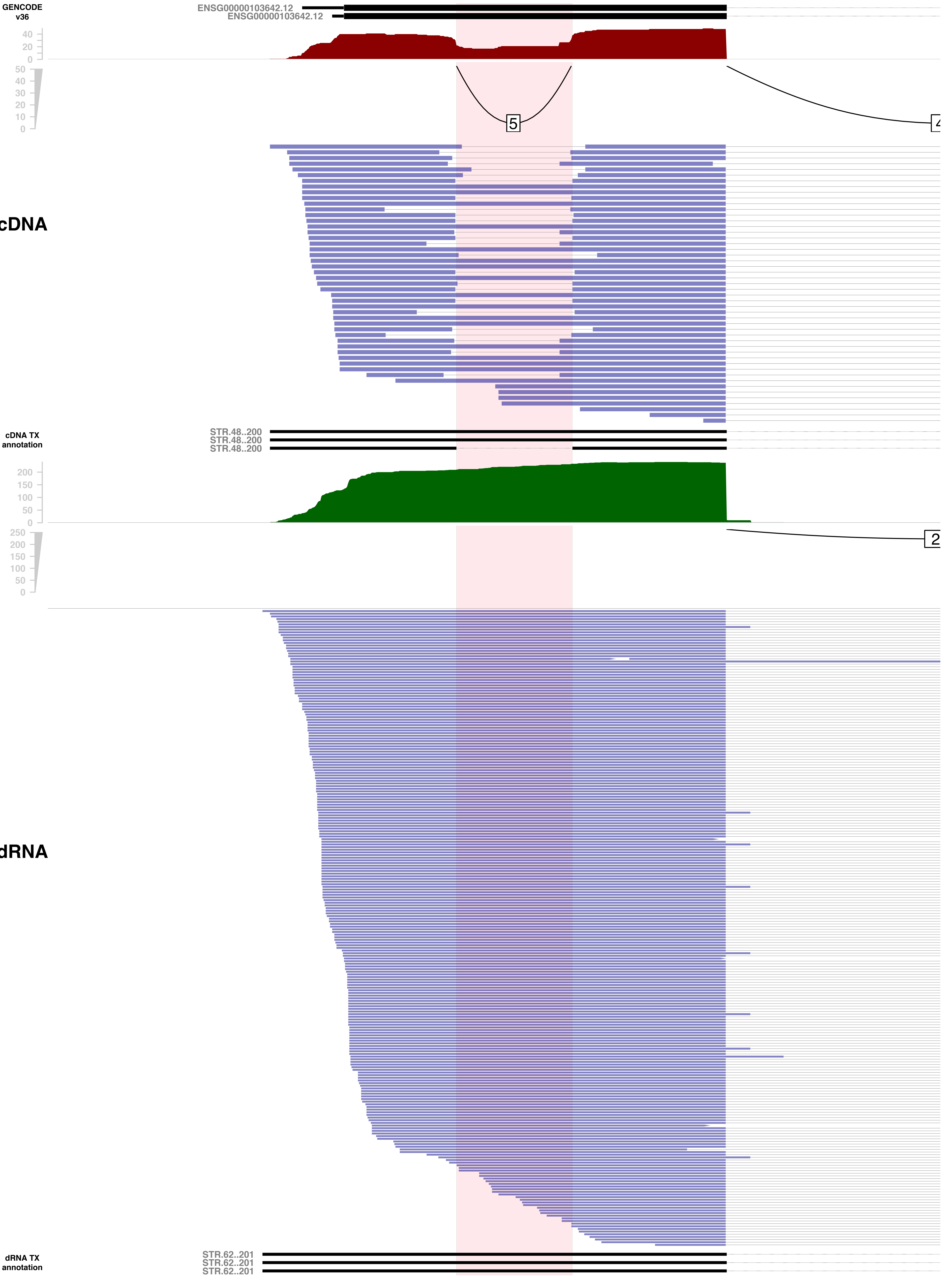

chr17:3663563−3663657:− TAX1BP3,P2RX5−TAX1BP3
Cell line: A549
Direct repeat sequence: GACCG

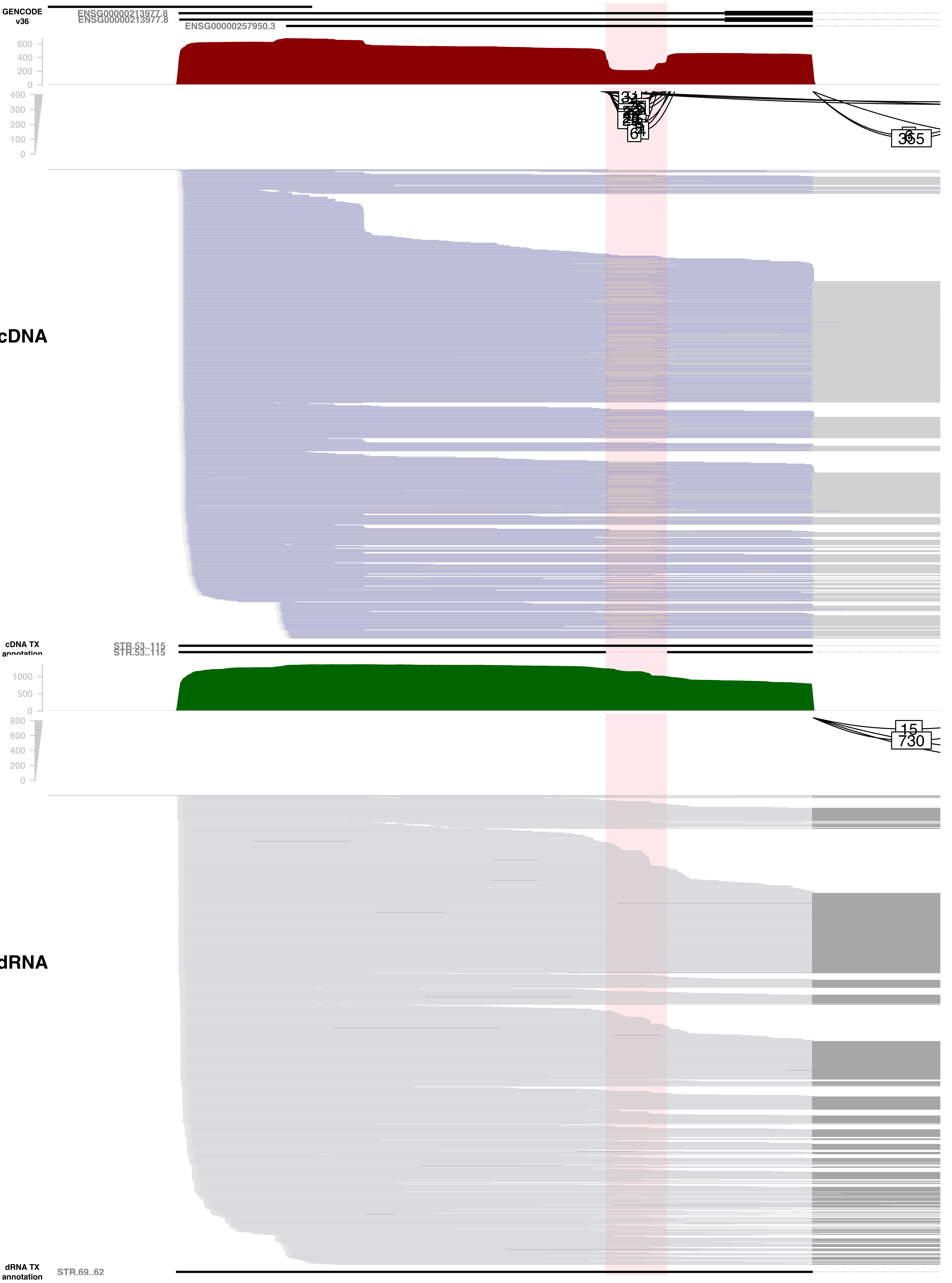

chr19:35746759−35746910:+ PSENEN,AC002398.9

Cell line: A549

Direct repeat sequence: CCTG

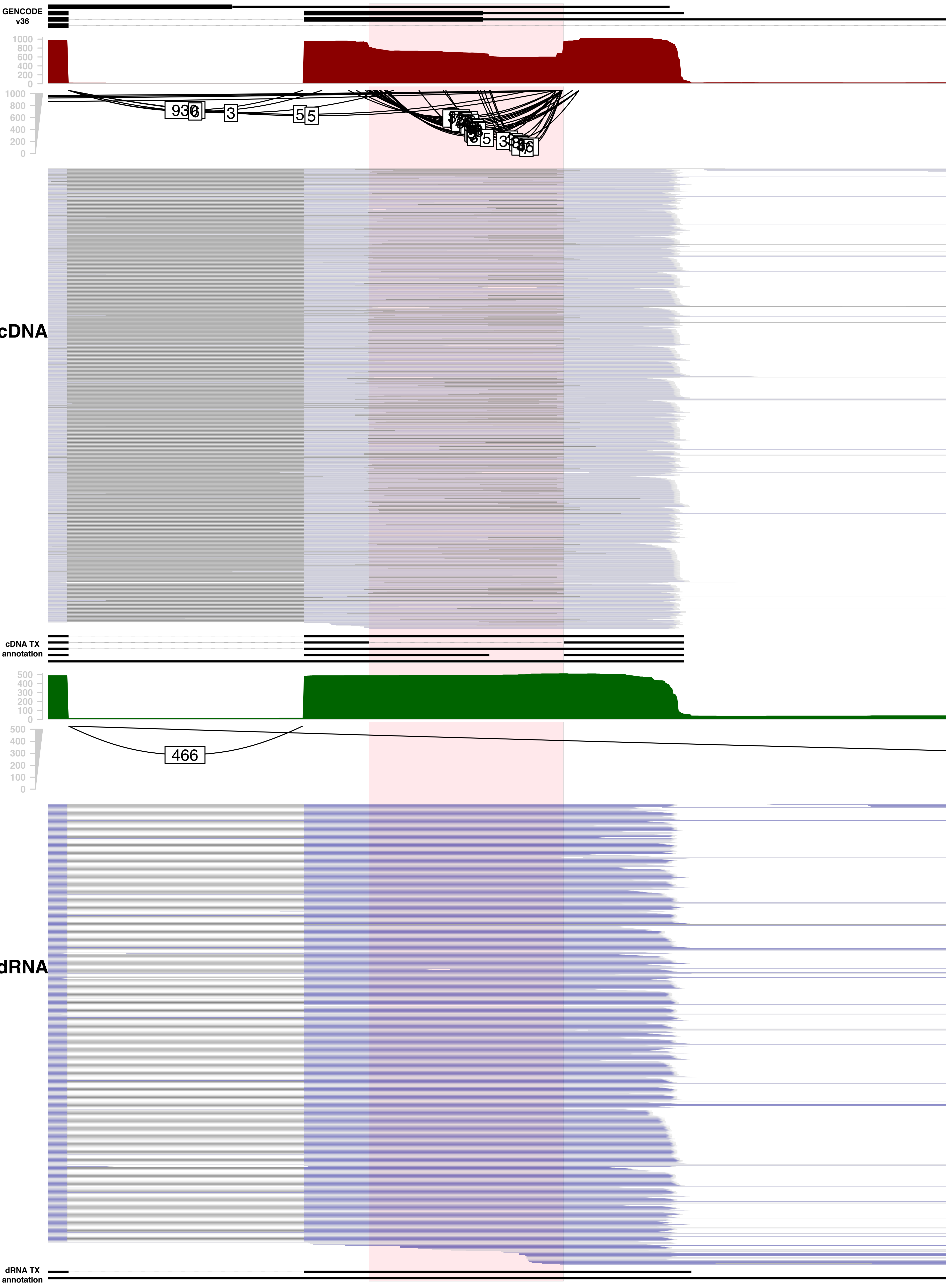

chr19:35746853−35746910:+ PSENEN,AC002398.9
Cell line: A549

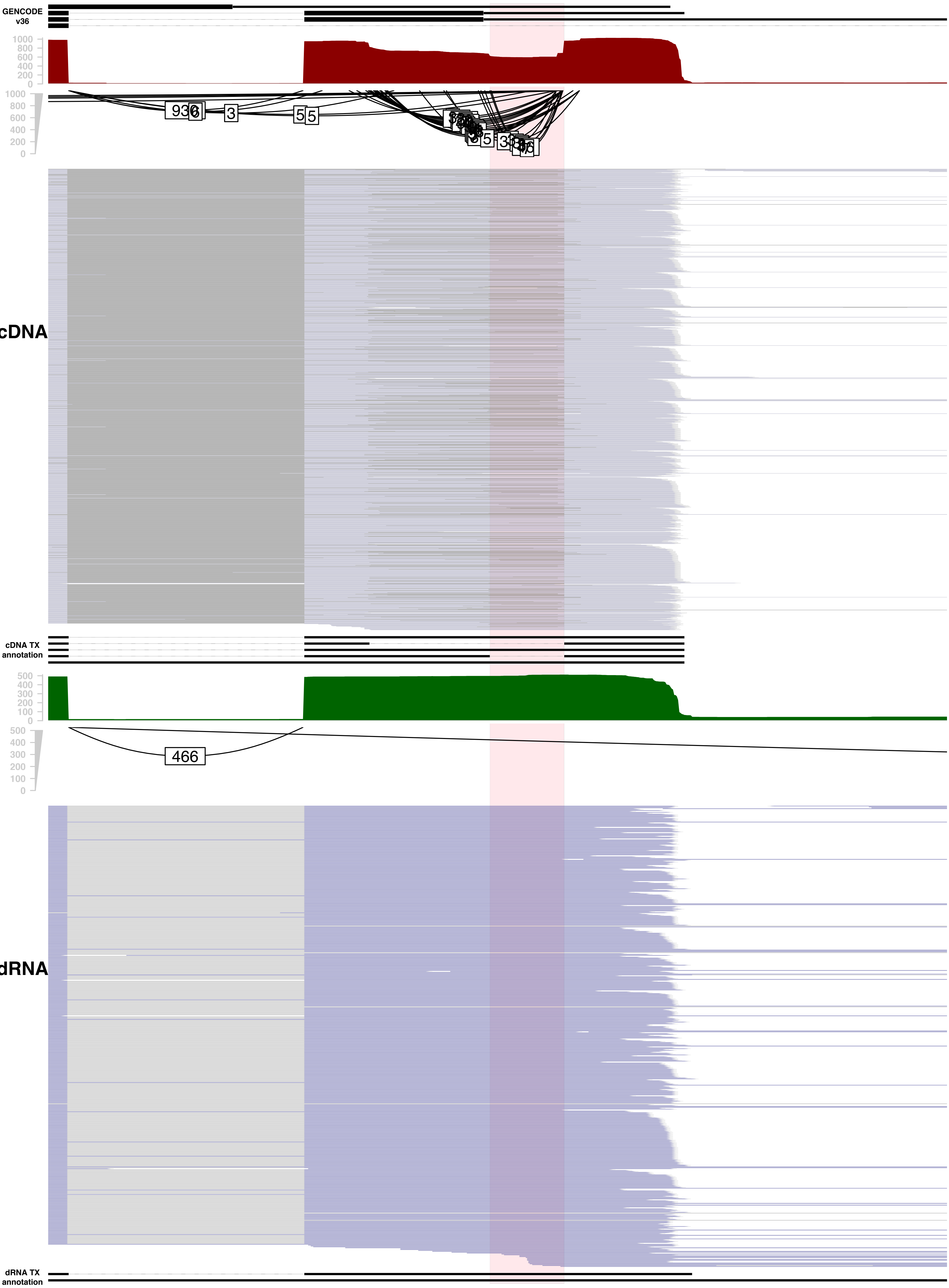

chr4:56459858−56460922:+ PAICS
Cell line: A549

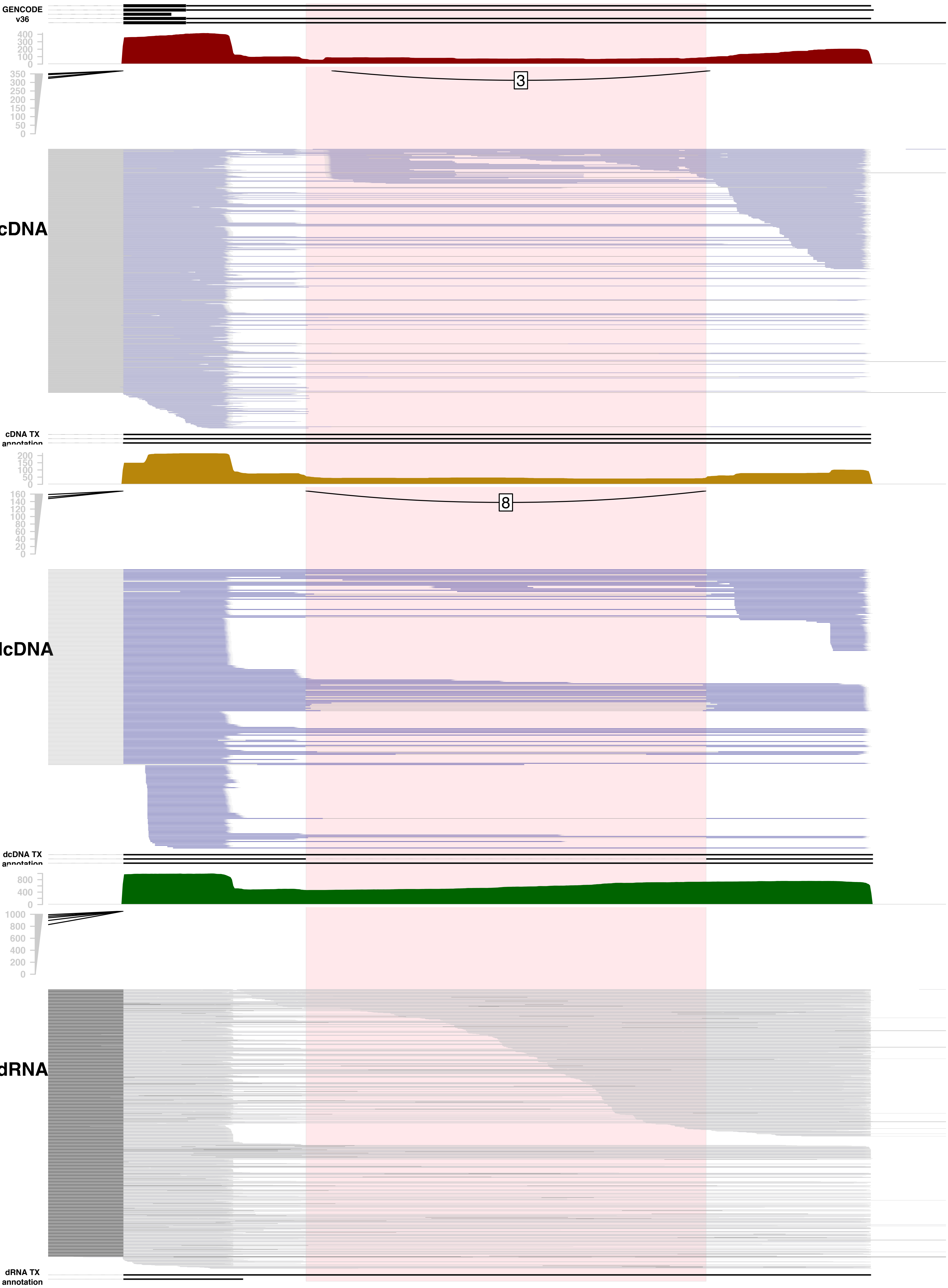

chr1:8012958−8013358:− ERRFI1

Cell line: Hct116

Direct repeat sequence: GGGAAGC

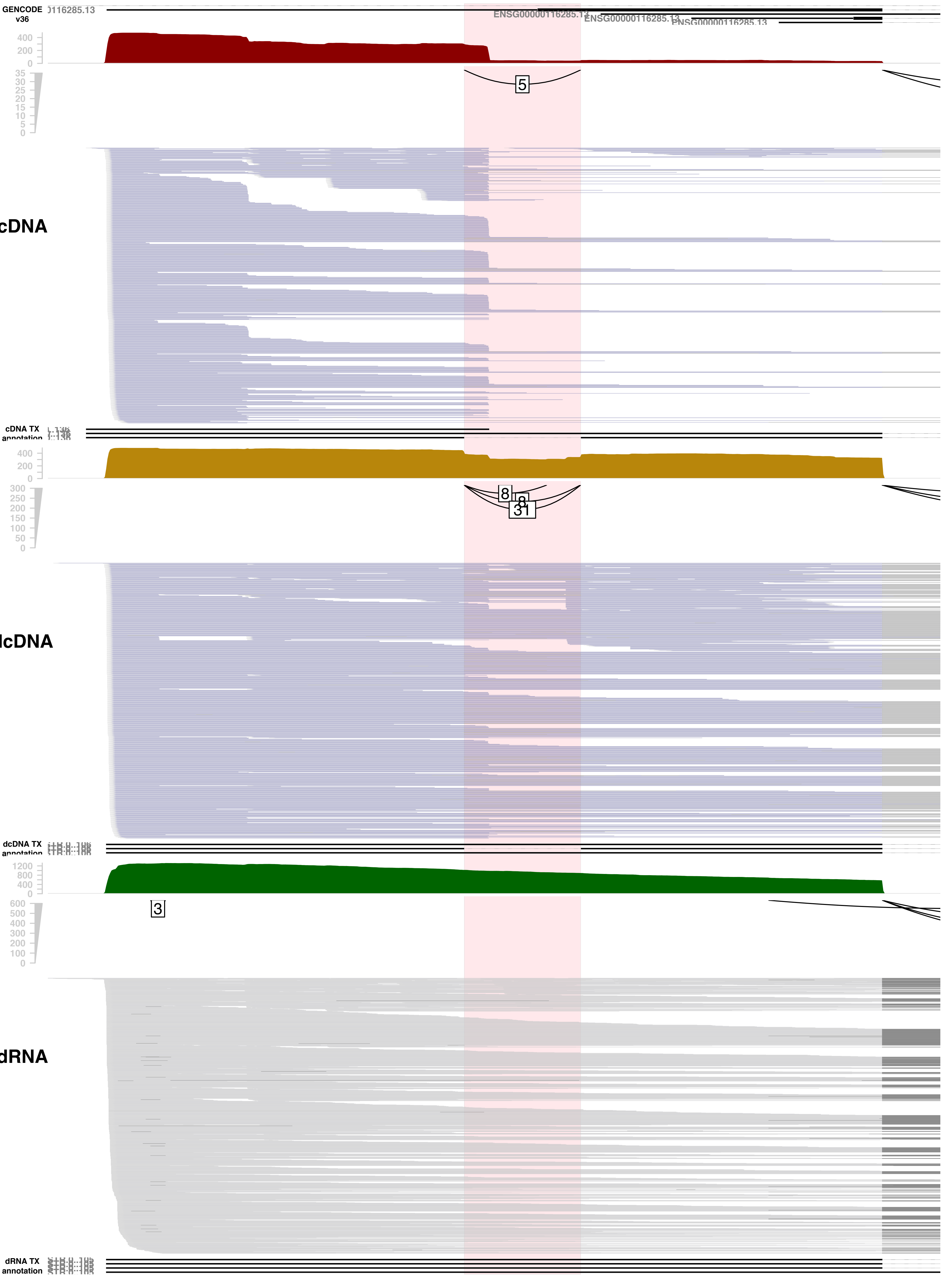

chr1:28335161−28335708:+ MED18
Cell line: Hct116
Direct repeat sequence: CAGC

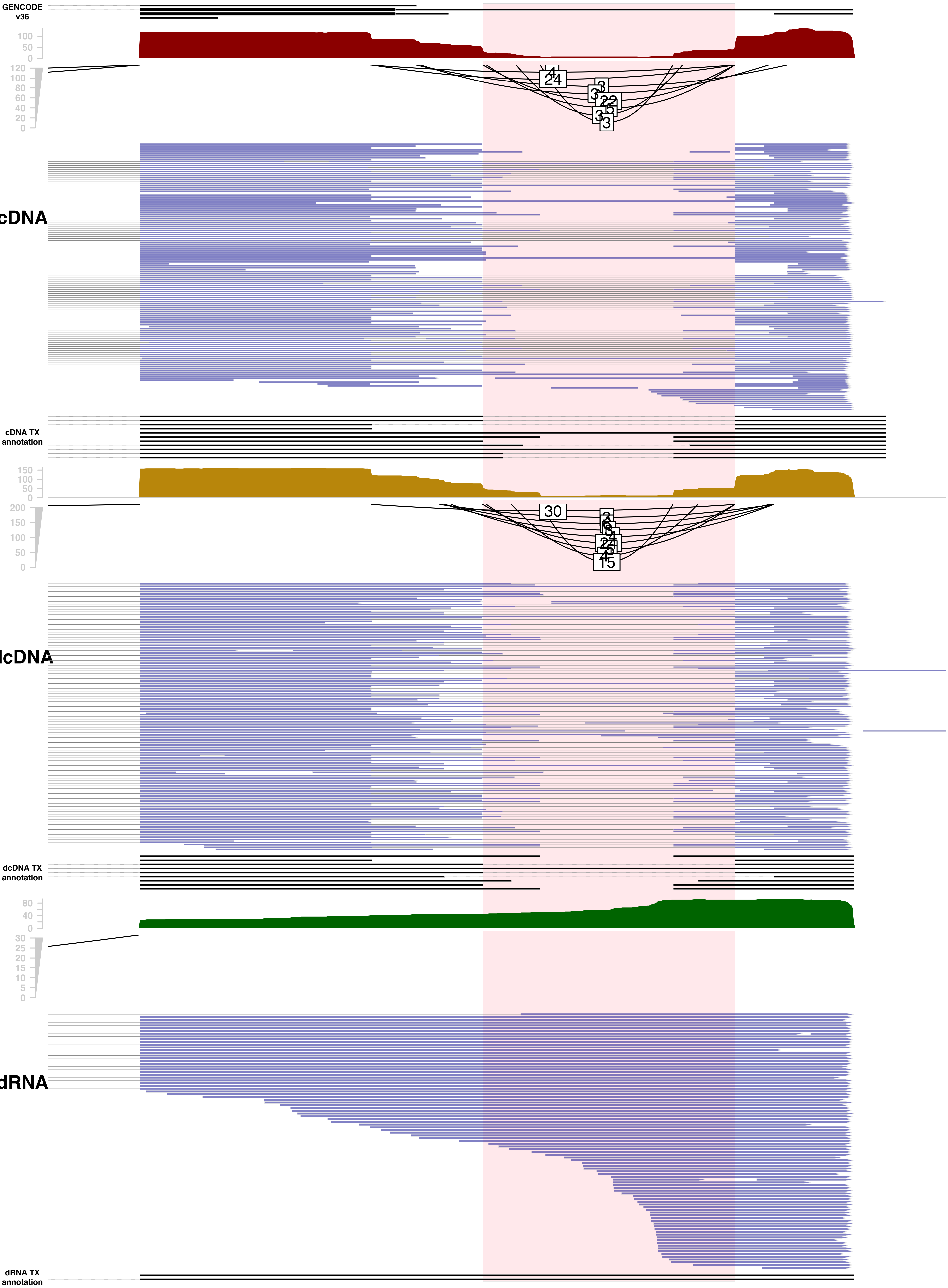

chr5:163437659−163437758:+ CCNG1
Cell line: Hct116

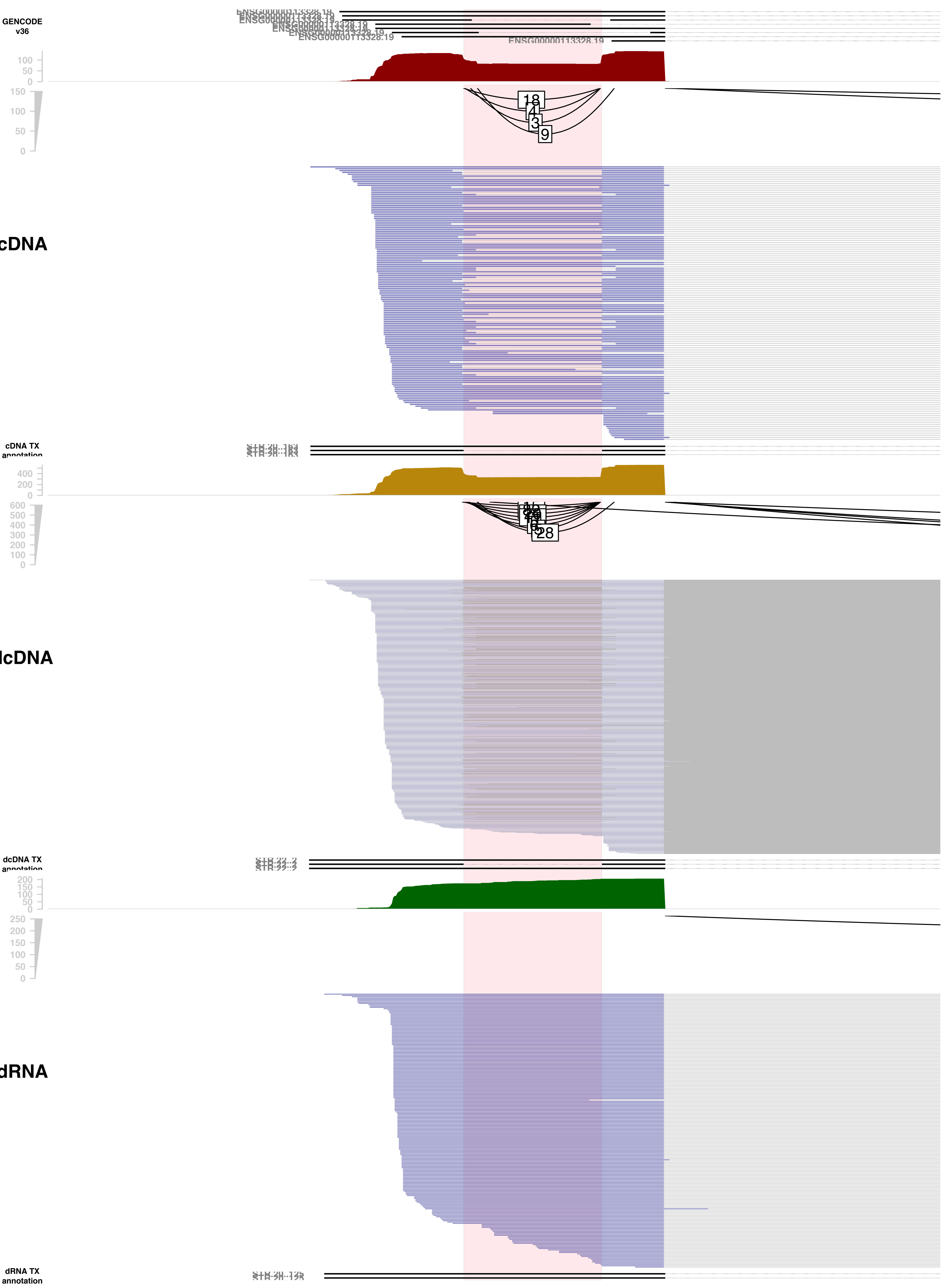

chr6:170584410−170584525:− PDCD2
Cell line: Hct116

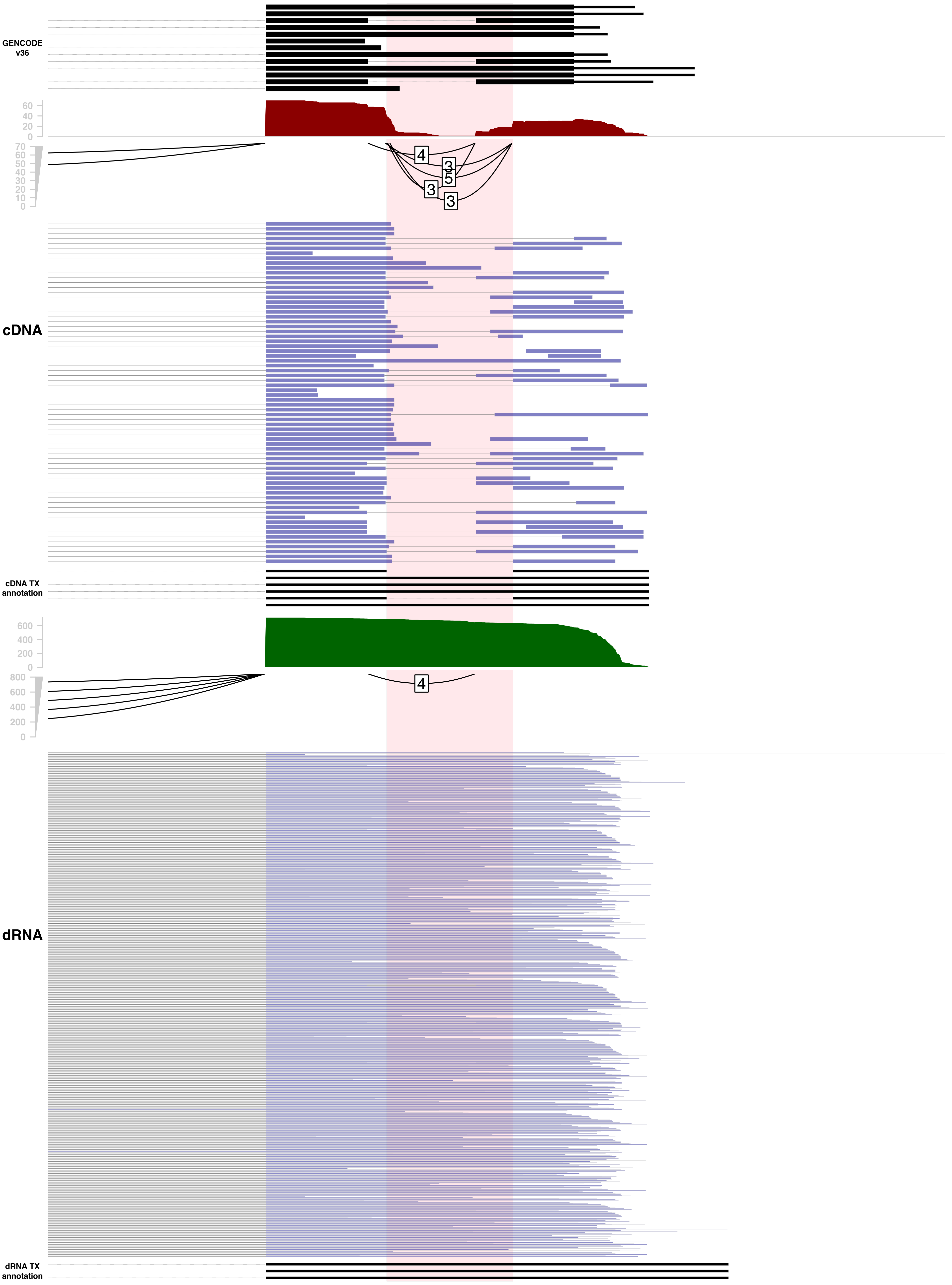

chr7:157139165−157139221:+ UBE3C
Cell line: Hct116
Direct repeat sequence: GCCC

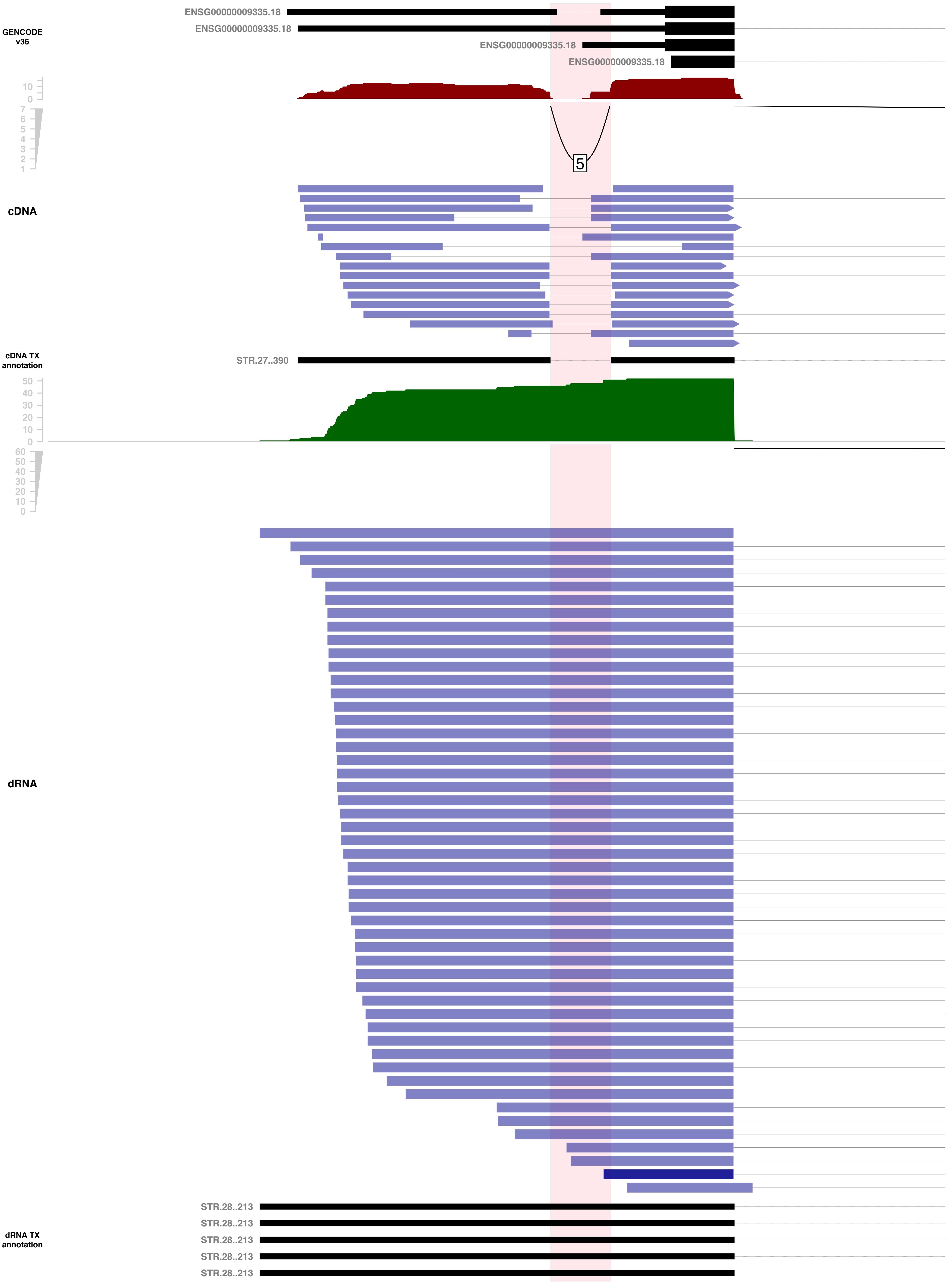

chr8:47976979−47977736:+ MCM4
Cell line: Hct116

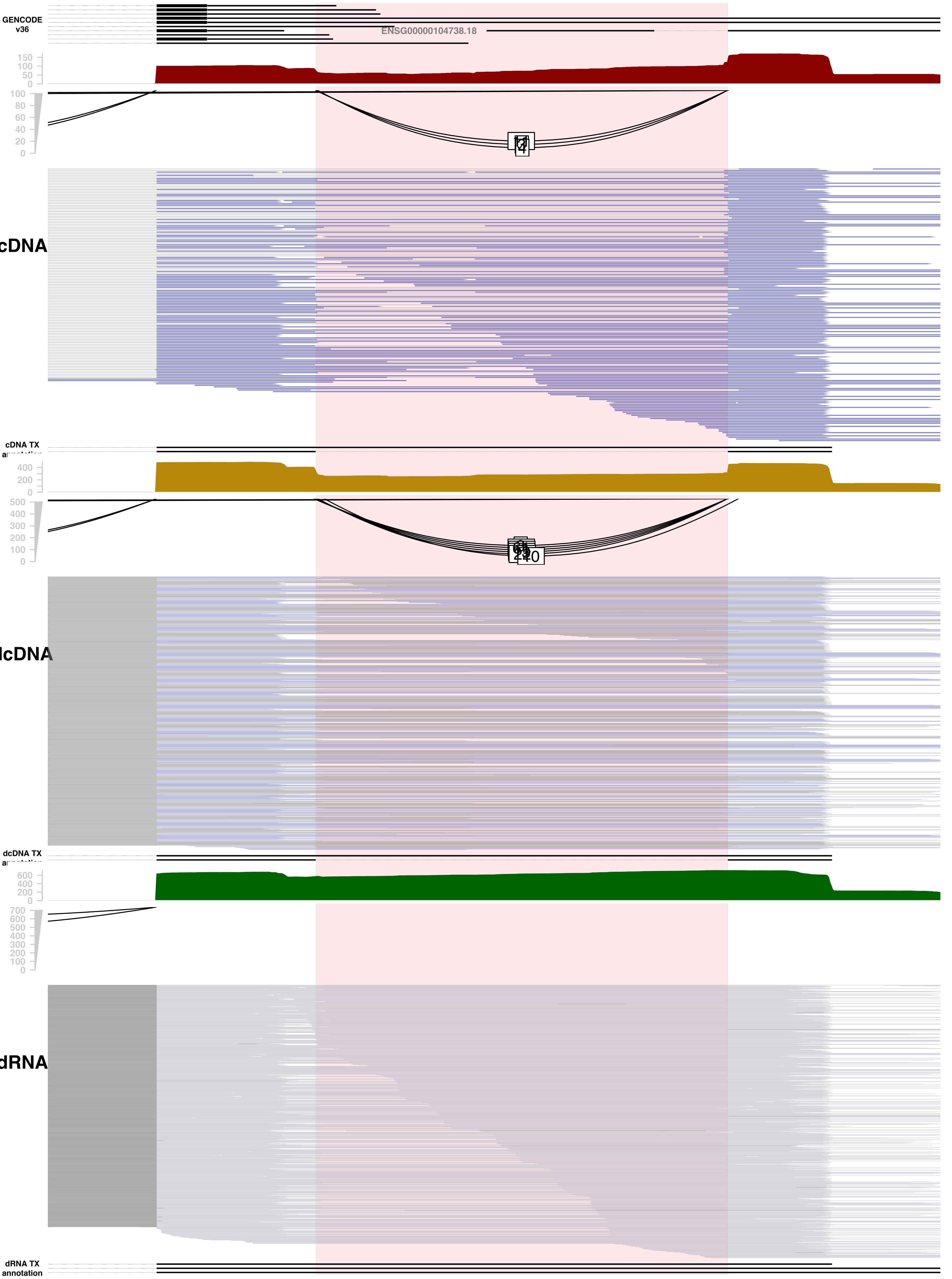

chr14:31447249−31447389:− RP11−176H8.1,DTD2

Cell line: Hct116

Direct repeat sequence: CAGCAC

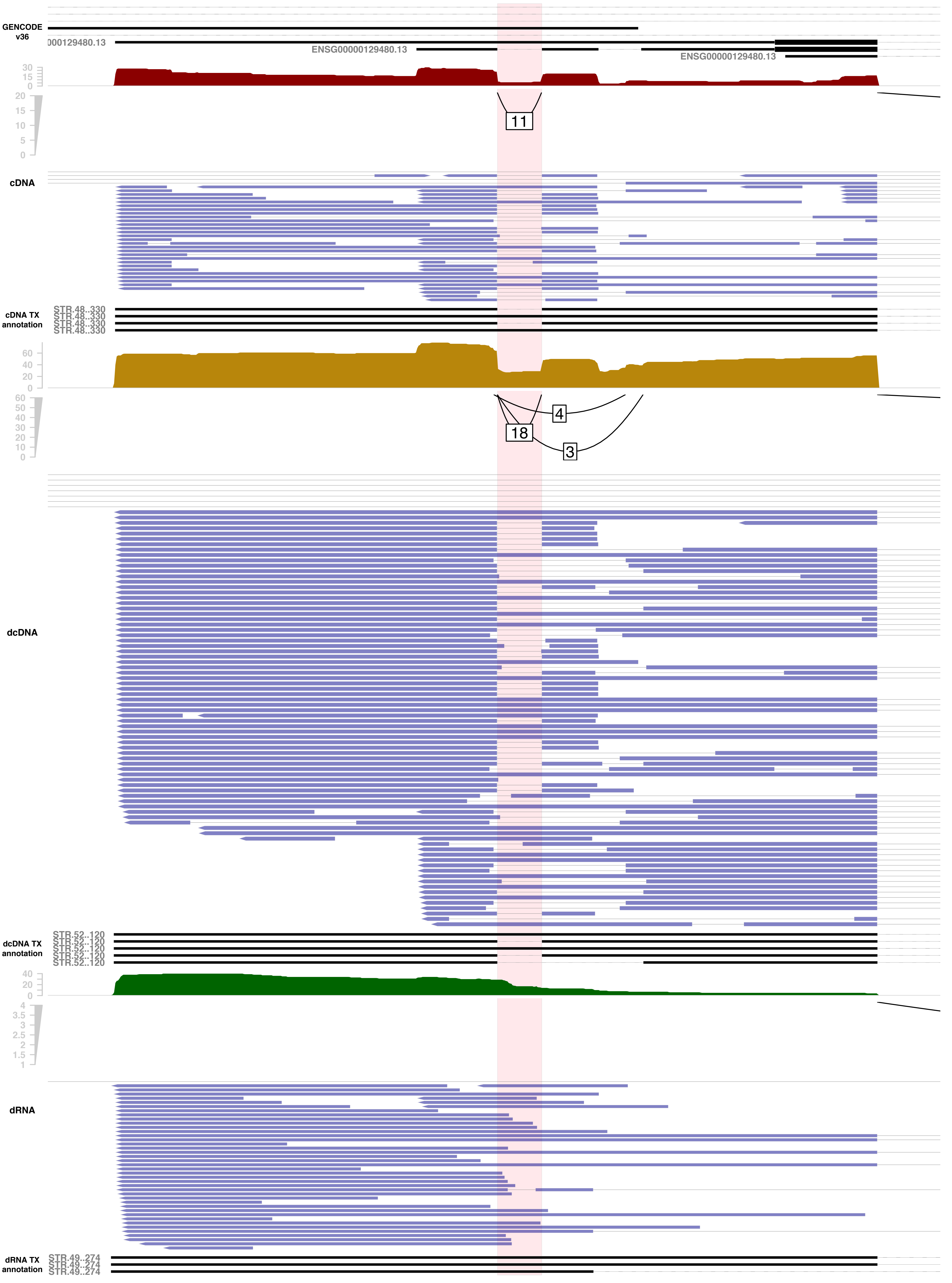

chr19:35746759−35746910:+ PSENEN,AC002398.9
Cell line: Hct116
Direct repeat sequence: CCTG

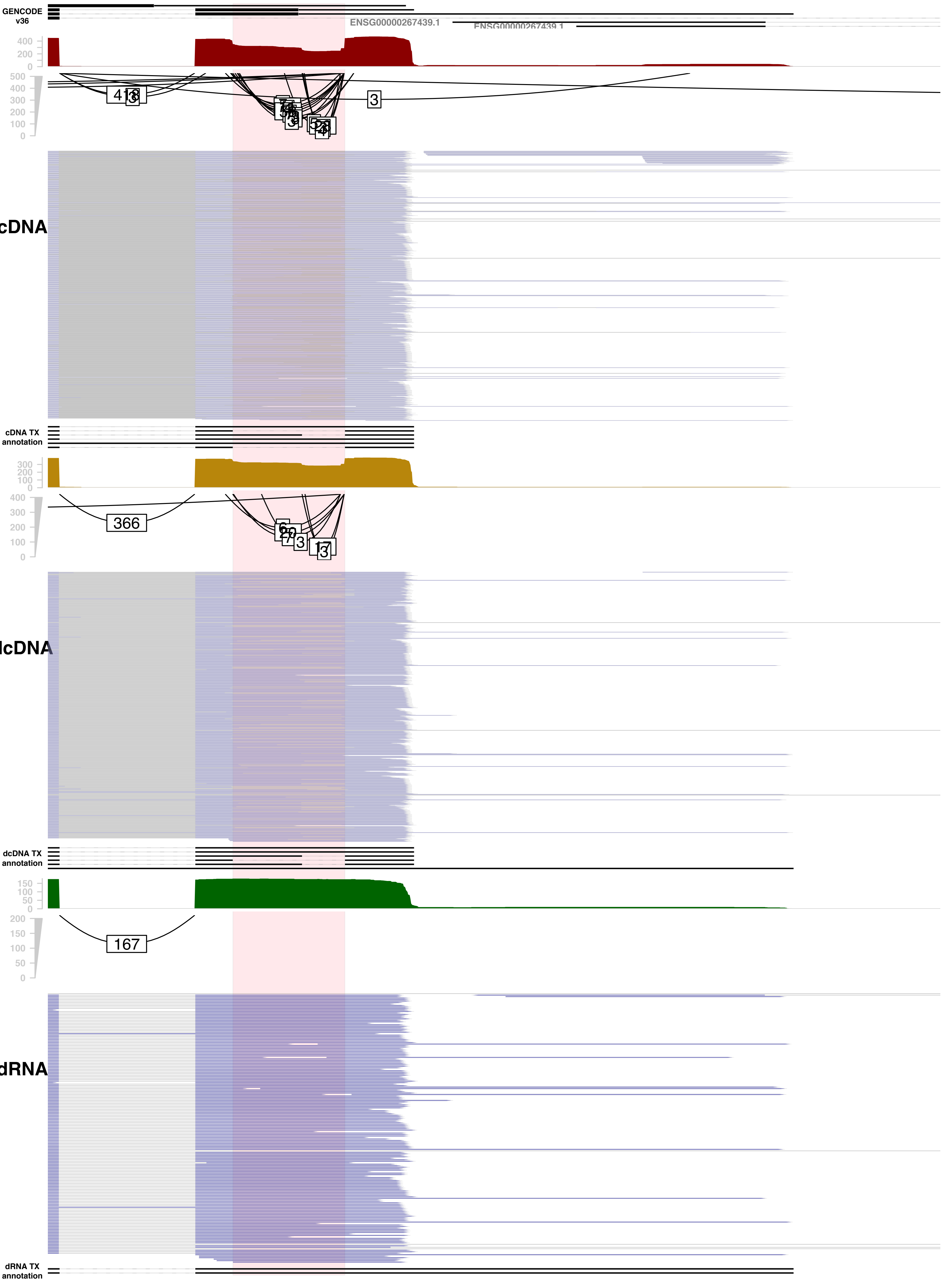

chr19:35746853−35746910:+ PSENEN,AC002398.9
Cell line: Hct116

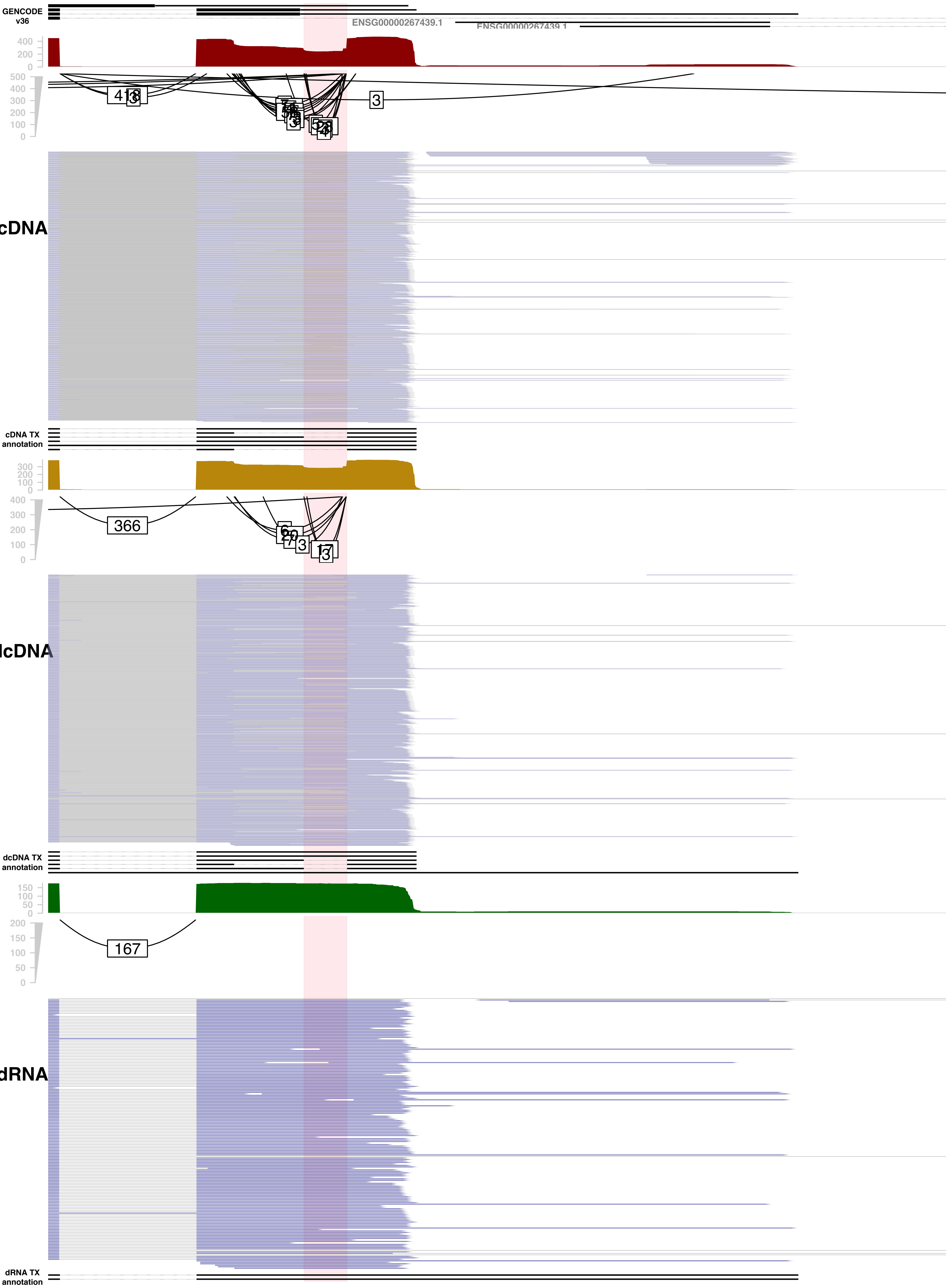

chr20:58891551−58891720:+ GNAS

Cell line: Hct116

Direct repeat sequence: CCGCC

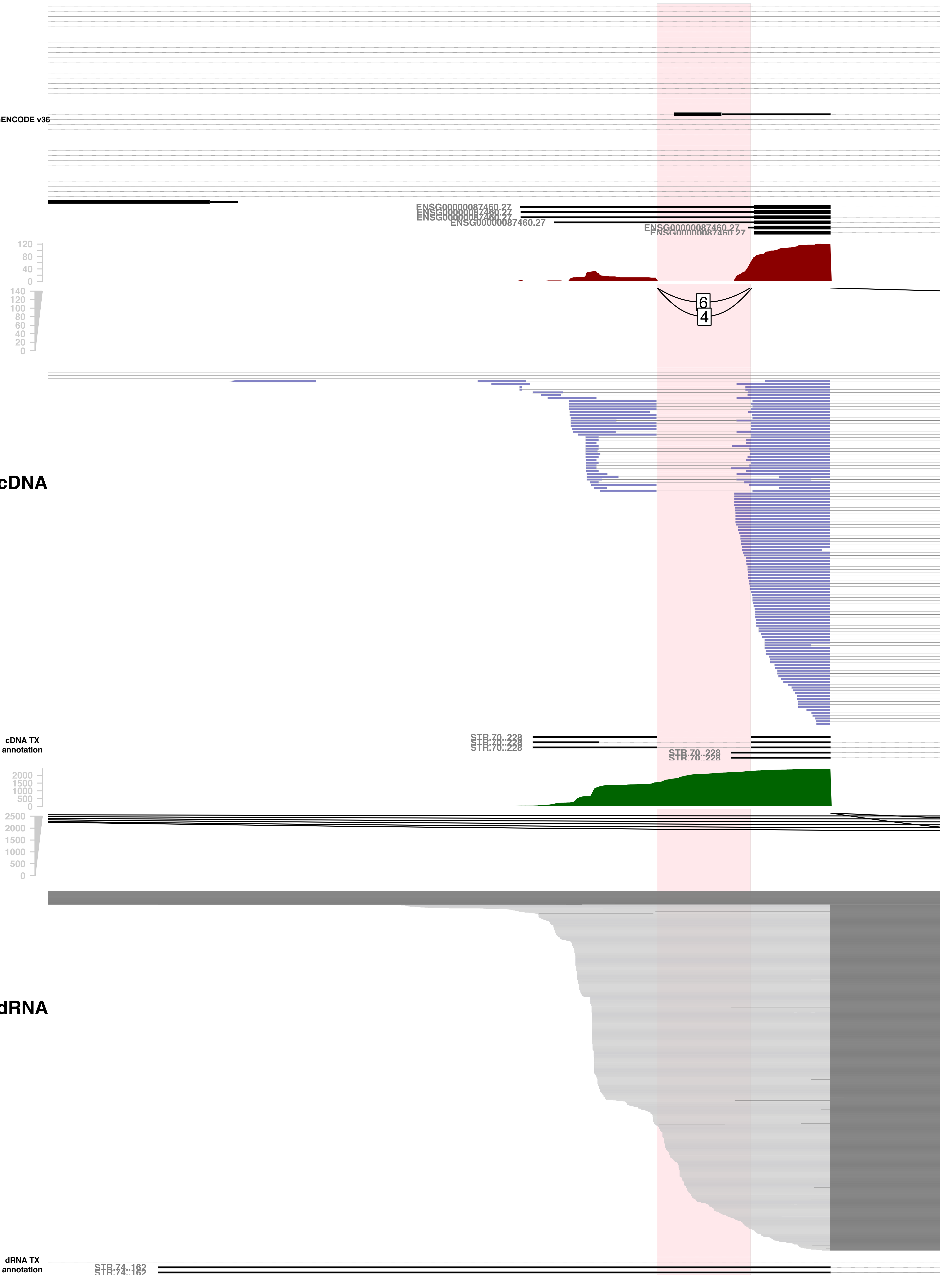

chr22:37807089−37807354:+ H1F0
Cell line: Hct116

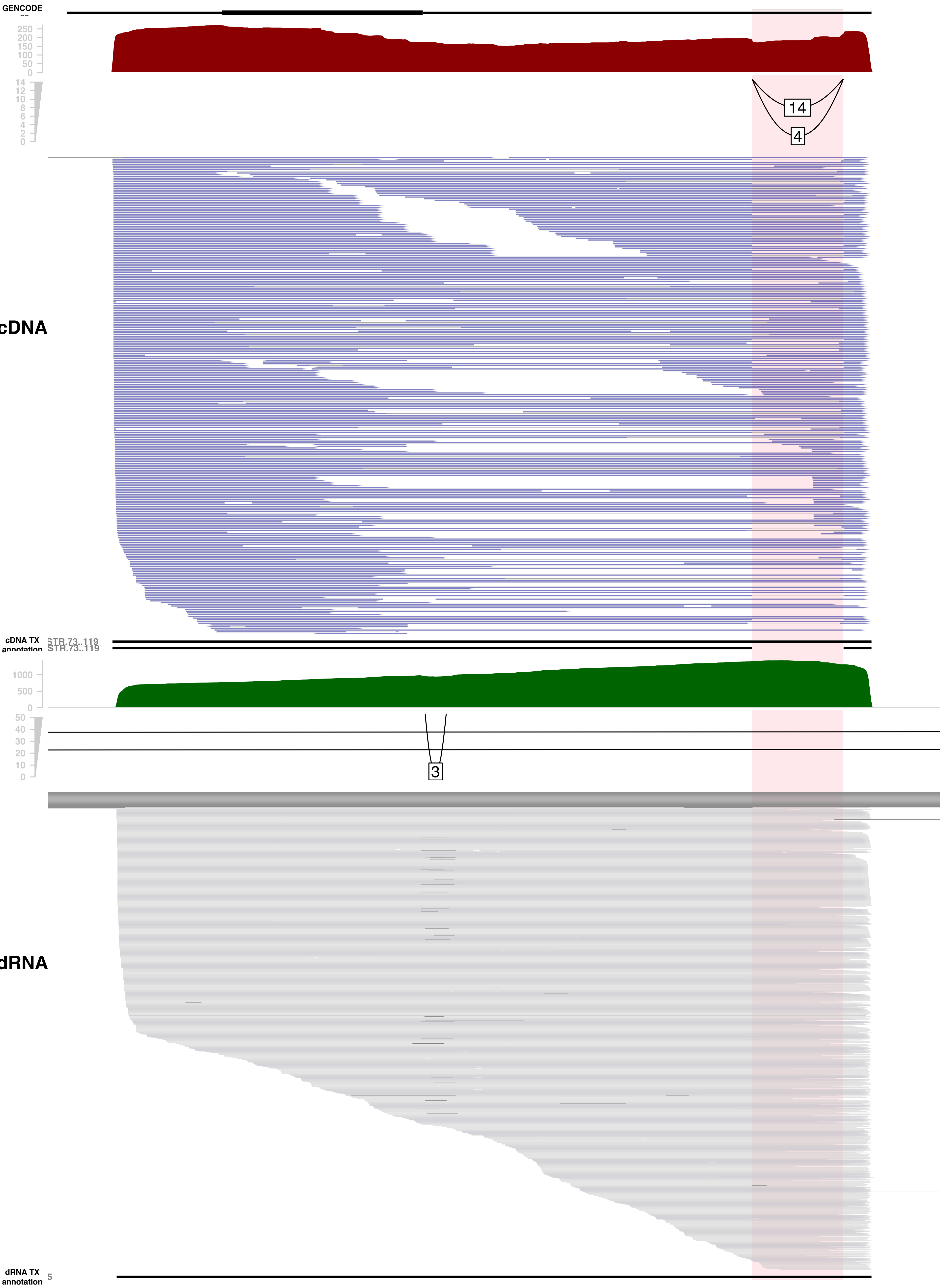

chr16:69167124−69168881:+ UTP4

Cell line: Hct116

Direct repeat sequence: ATGA

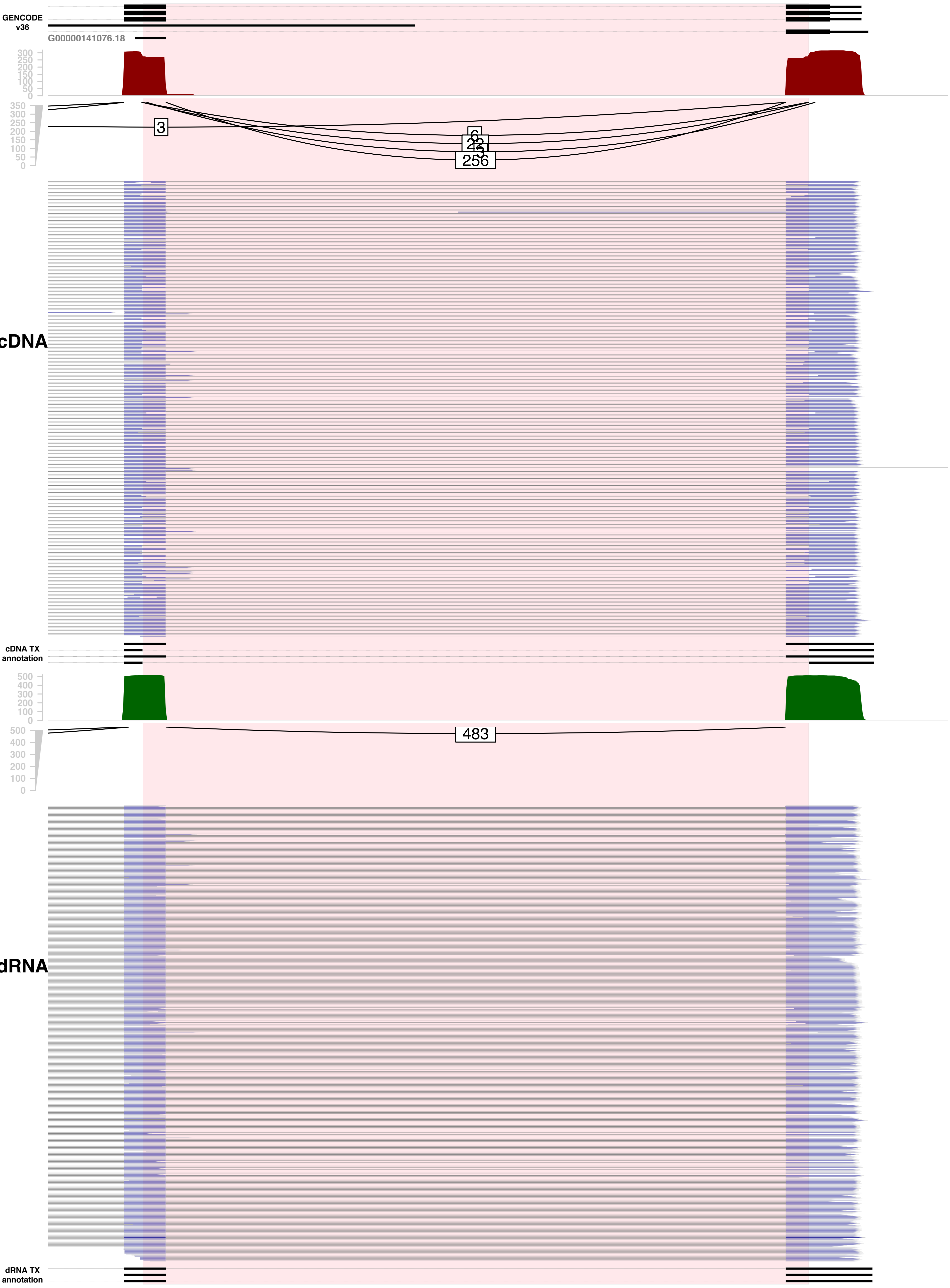

chr1:28335286−28335574:+ MED18
Cell line: Hct116
Direct repeat sequence: CCCA

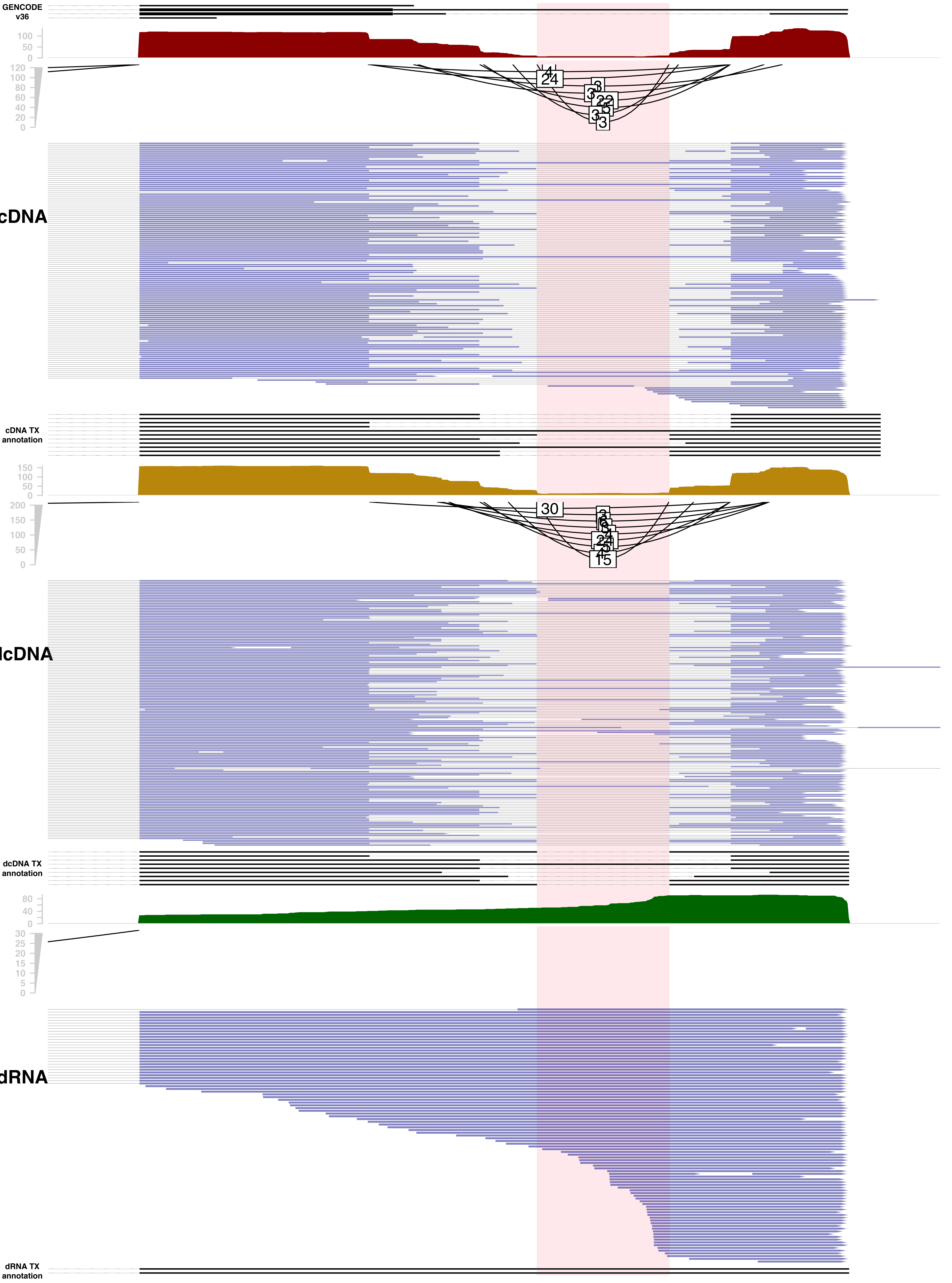

chr1:28334920−28335708:+ MED18
Cell line: Hct116
Direct repeat sequence: CAGCTA

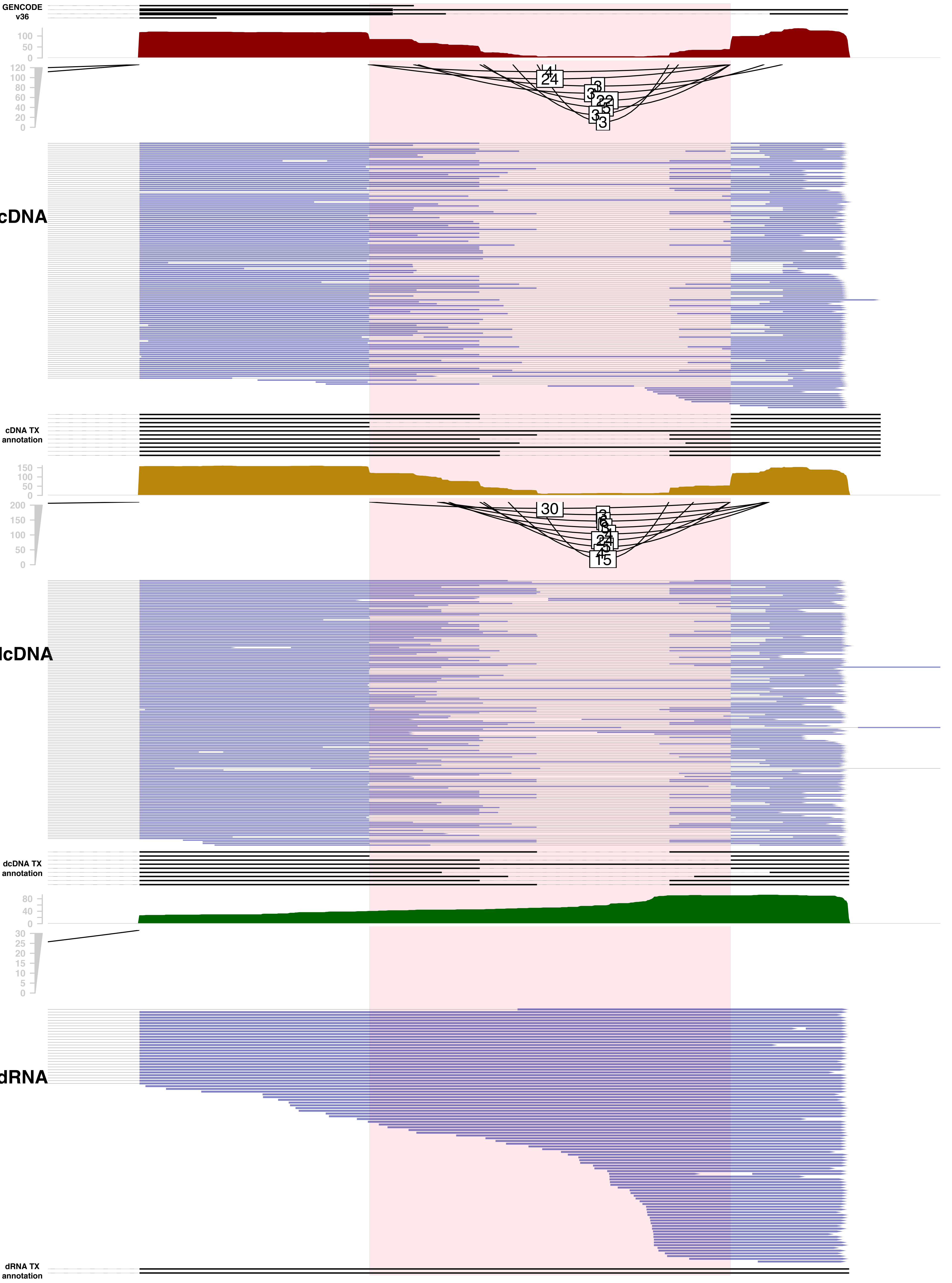

chr2:207754757−207754935:+ CCNYL1
Cell line: Hct116
Direct repeat sequence: CAGC

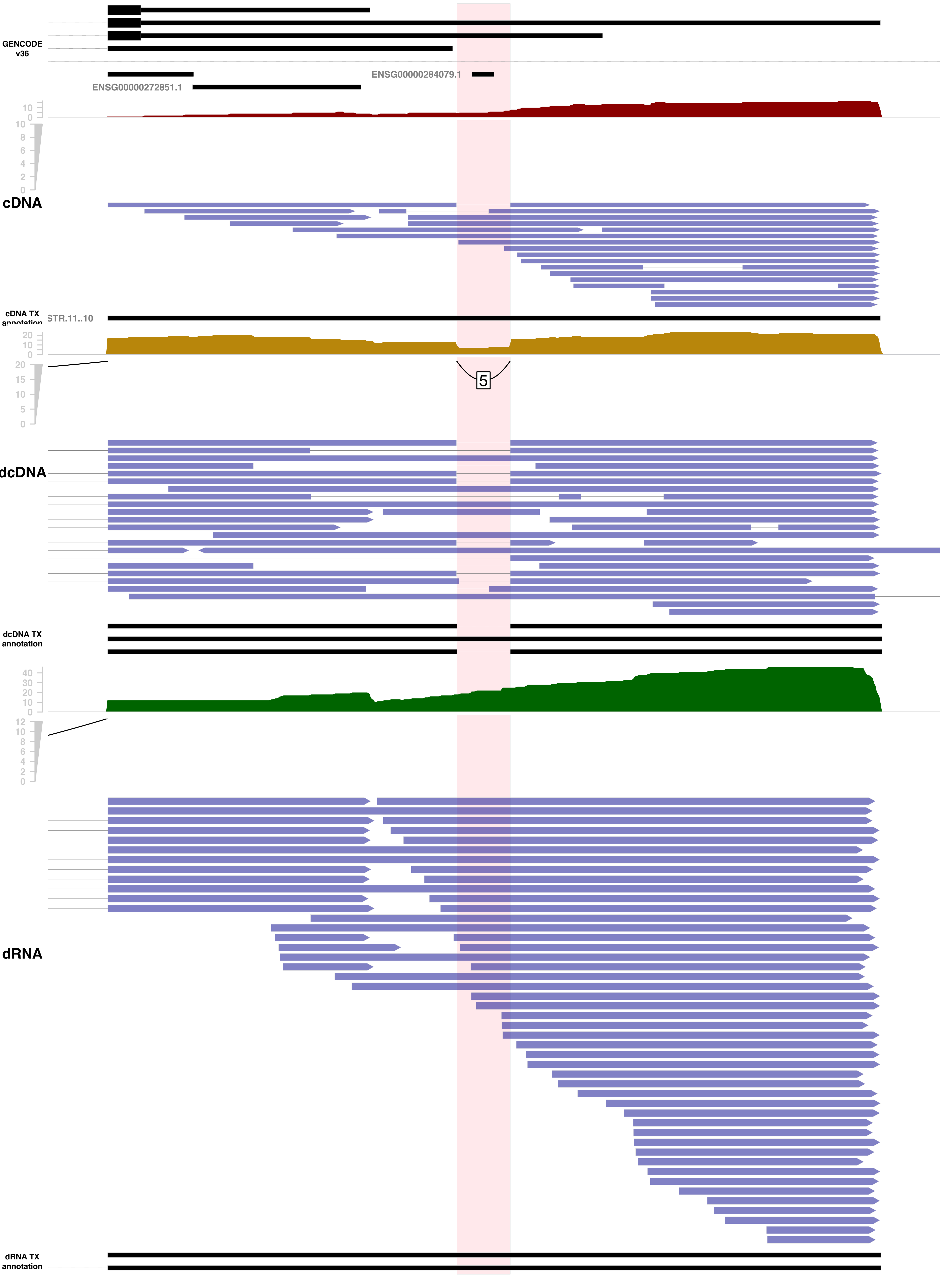

chr4:176331078−176331197:+ SPCS3

Cell line: Hct116

Direct repeat sequence: CAGC

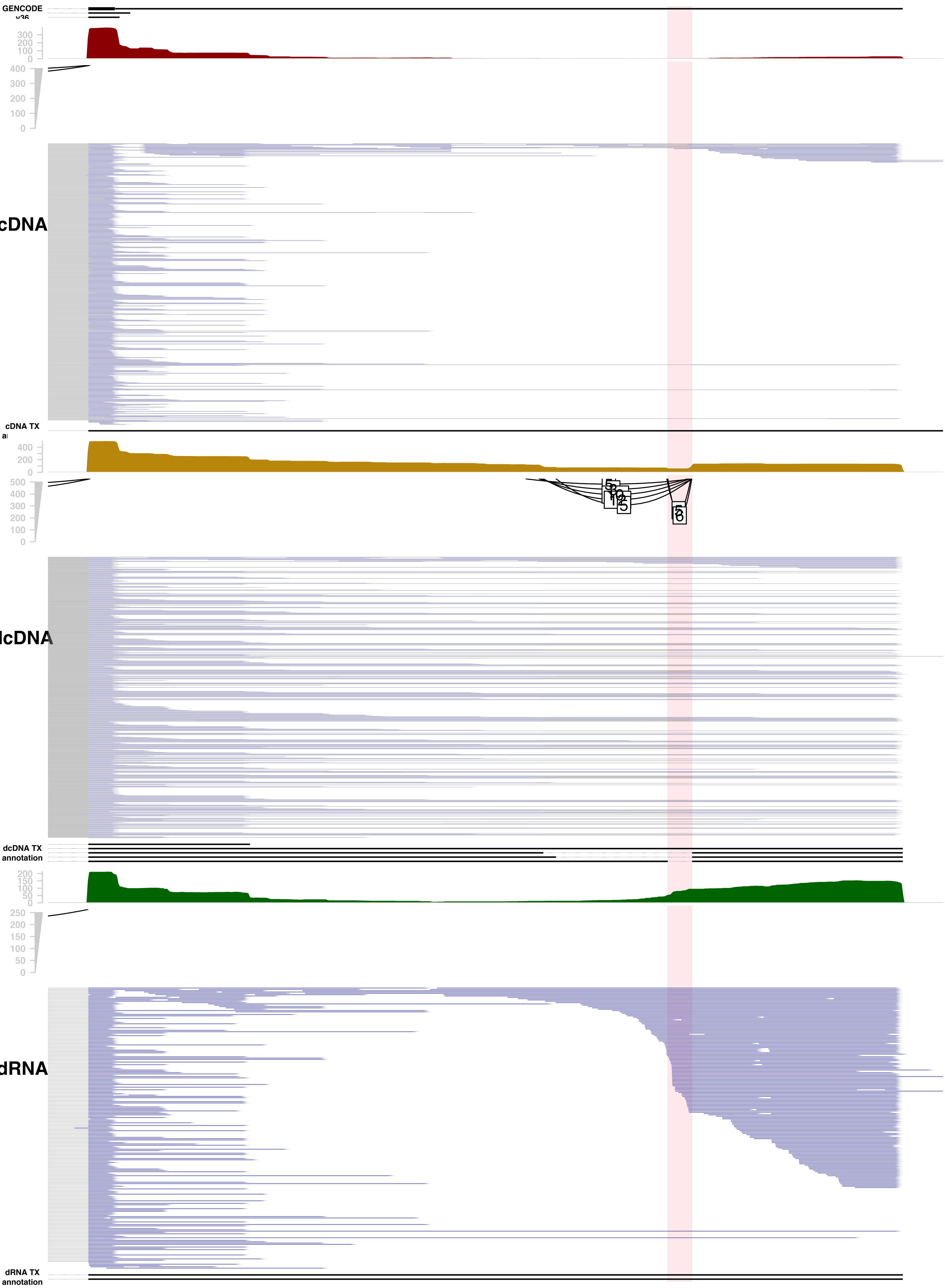

chr5:126625543−126625746:+ PHAX

Cell line: Hct116

Direct repeat sequence: GCTG

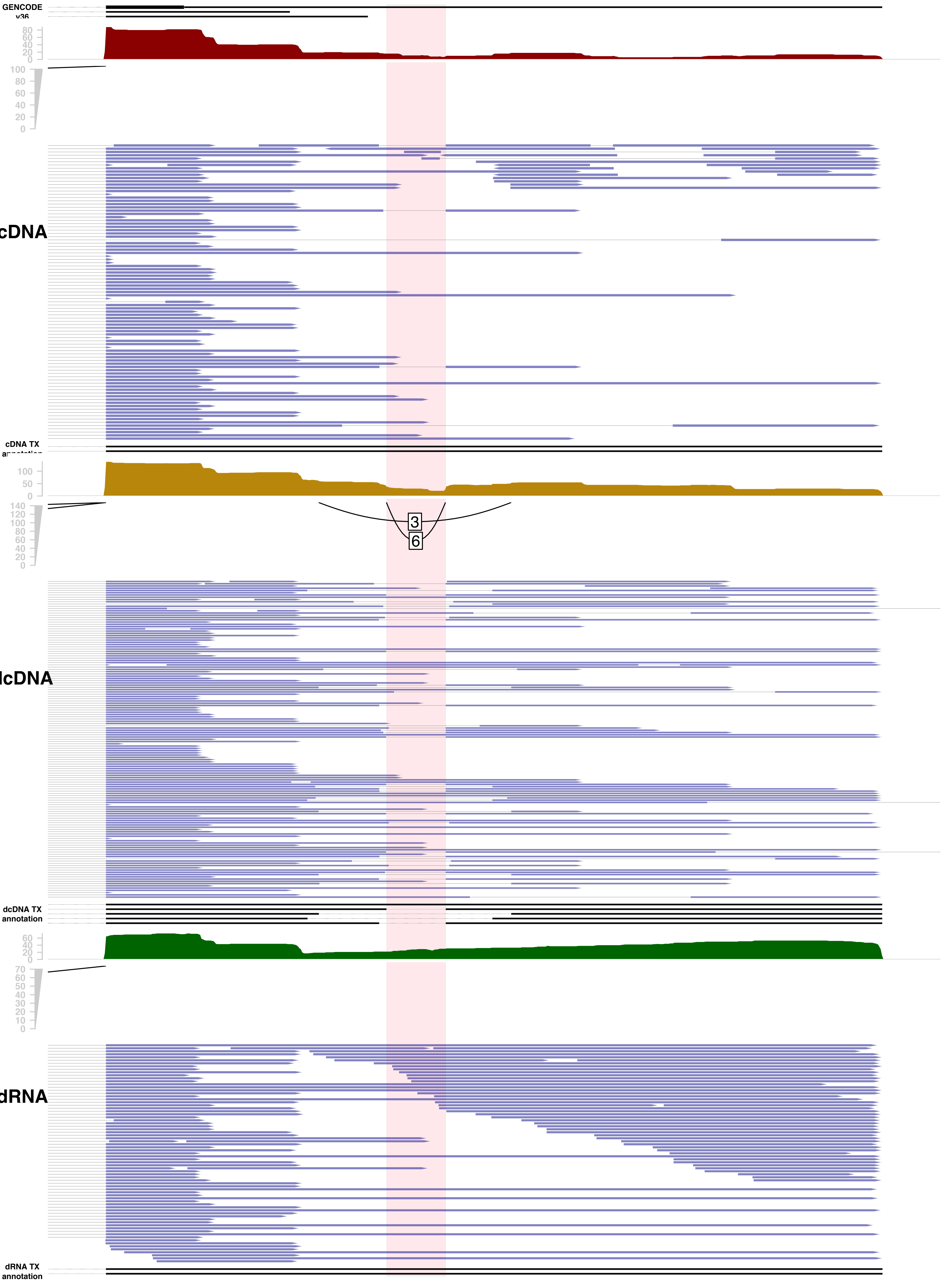

chr6:42887275−42888373:+ RPL7L1

Cell line: Hct116

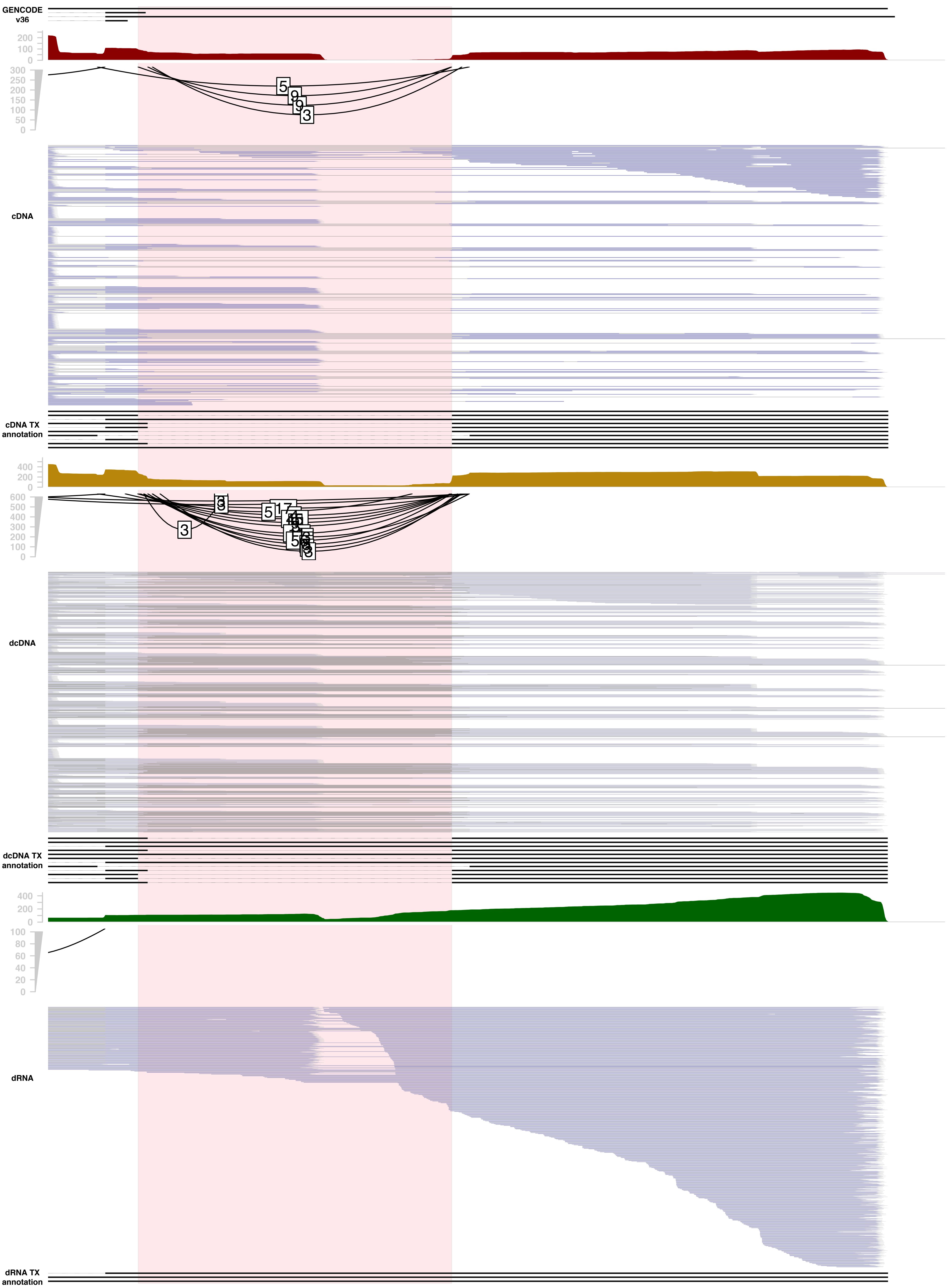

chr6:170584411−170584491:− PDCD2
Cell line: Hct116

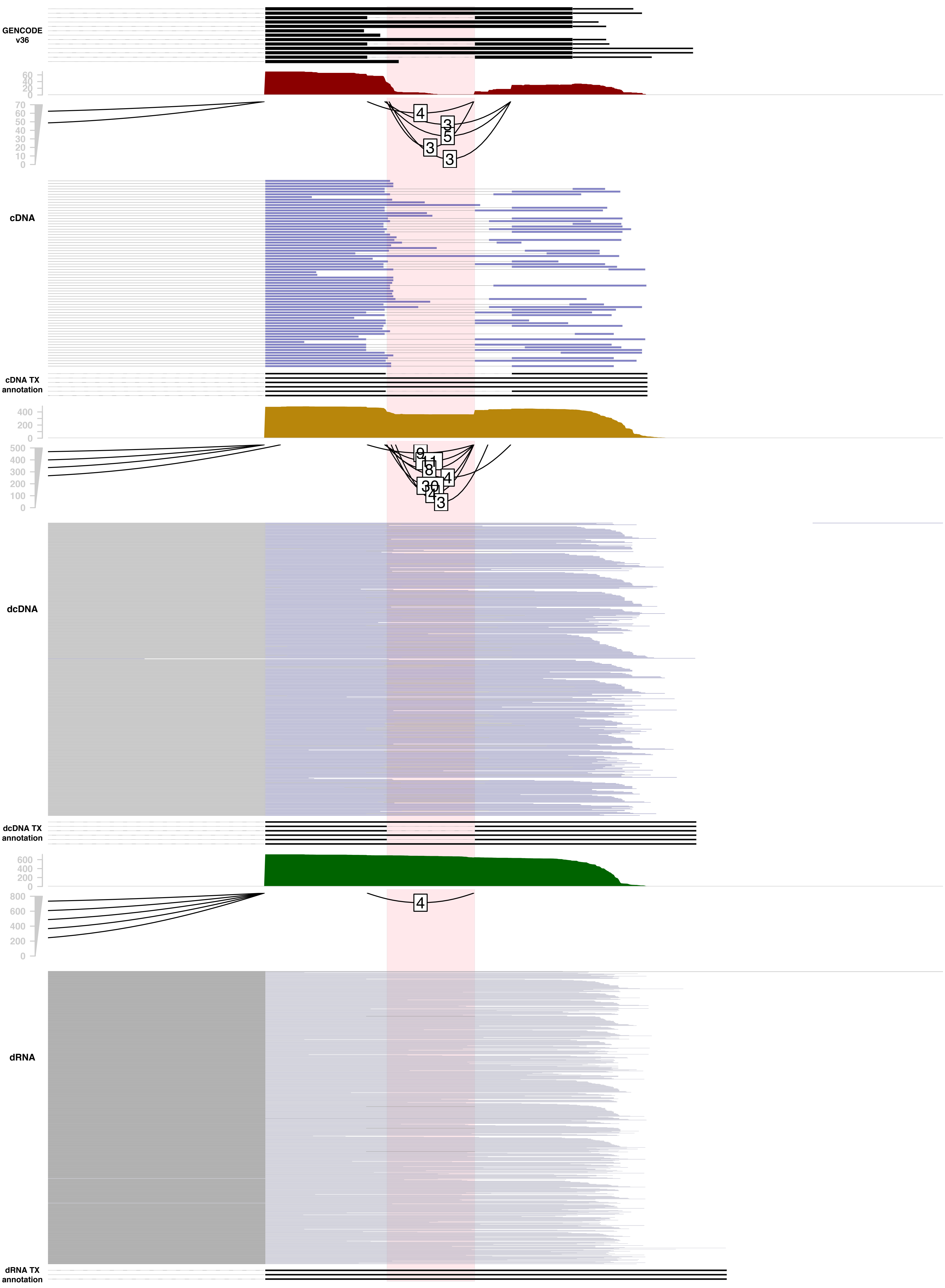

chr8:95245505−95246254:− C8orf37
Cell line: Hct116
Direct repeat sequence: CAGC

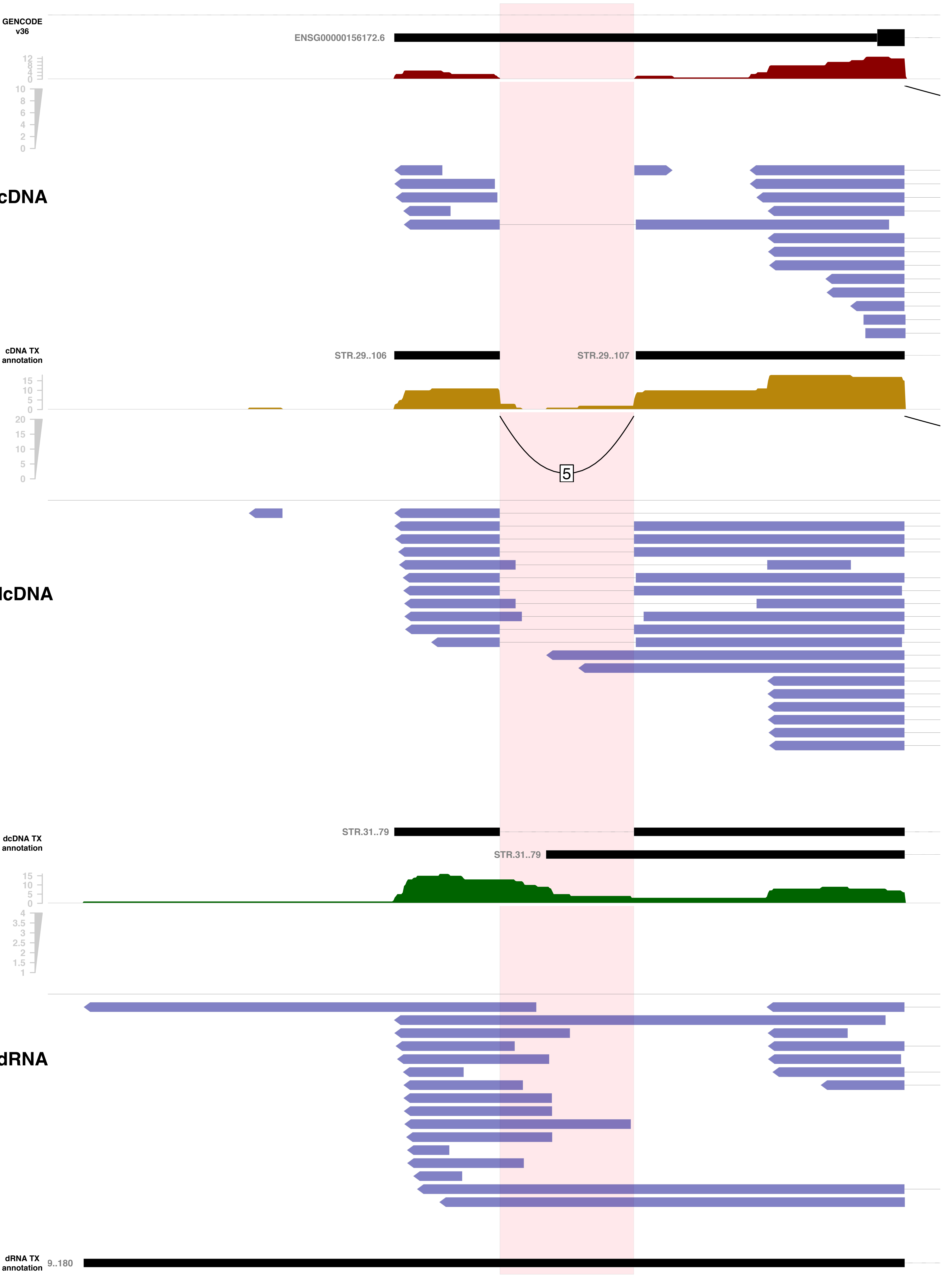

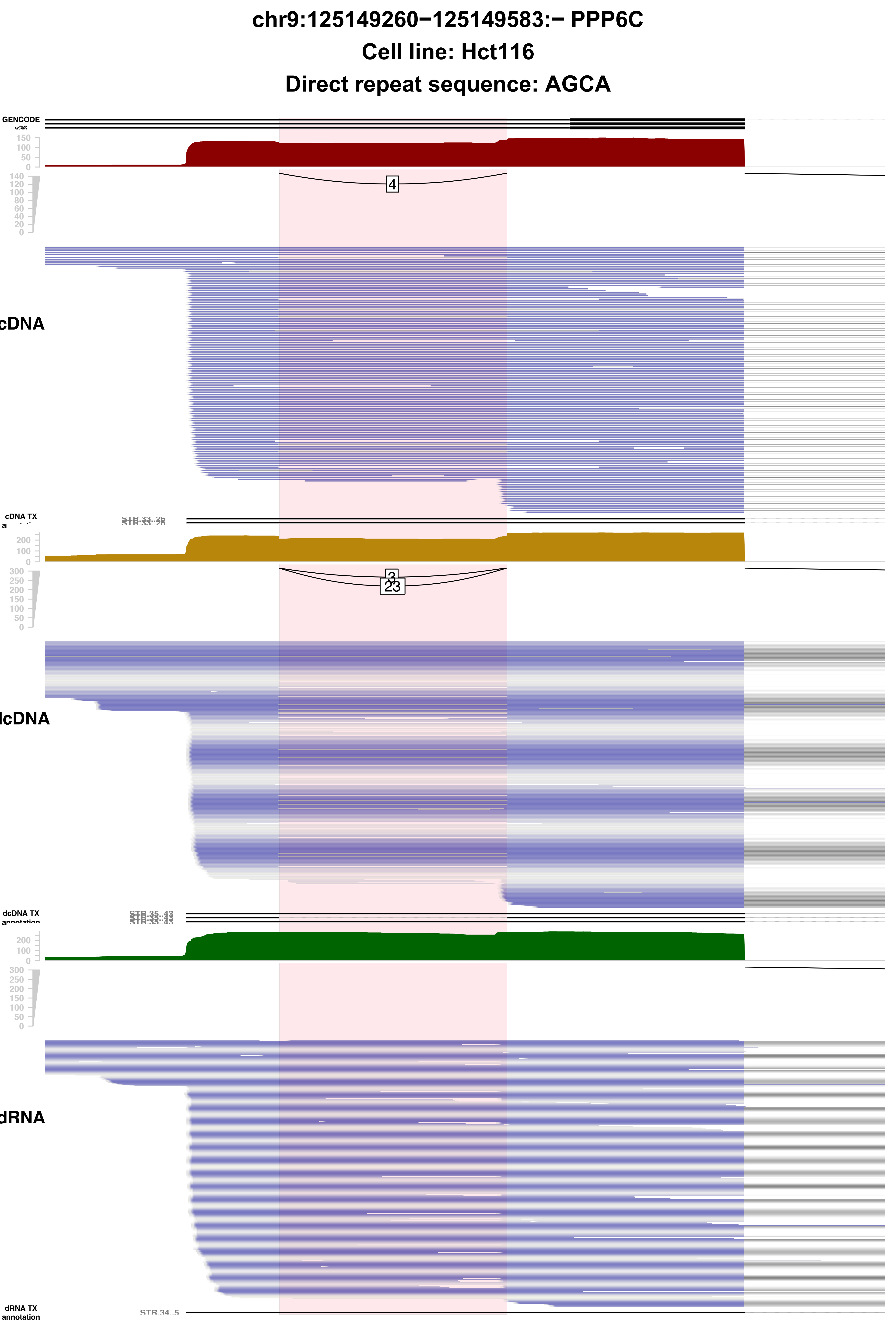
chr9:125149260−125149583:− PPP6C
Cell line: Hct116
Direct repeat sequence: AGCA
GENCODE
150
100
50
0
140
120
100
80
60
40
20
0
4
cDNA
cDNA TX
200
100
0
300
250
200
150
100
50
0
3
23
dcDNA TX
200
100
0
300
250
200
150
100
50
0
dRNA
dRNA TX
annotation

chr11:60842626−60842700:+ CCDC86

Cell line: Hct116

Direct repeat sequence: GGTCAGCA

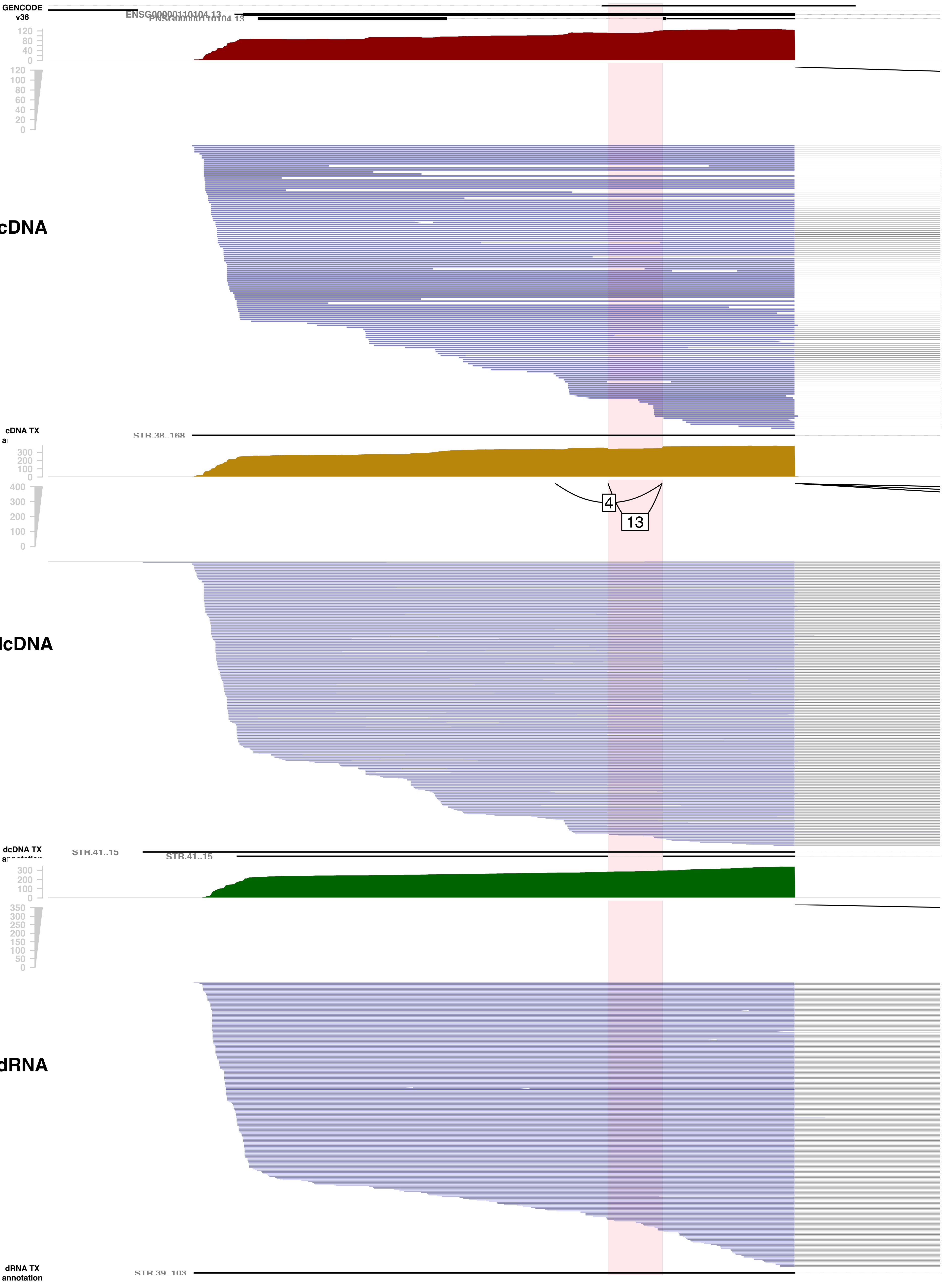

chr12:49351611−49353978:+ DNAJC22

Cell line: Hct116

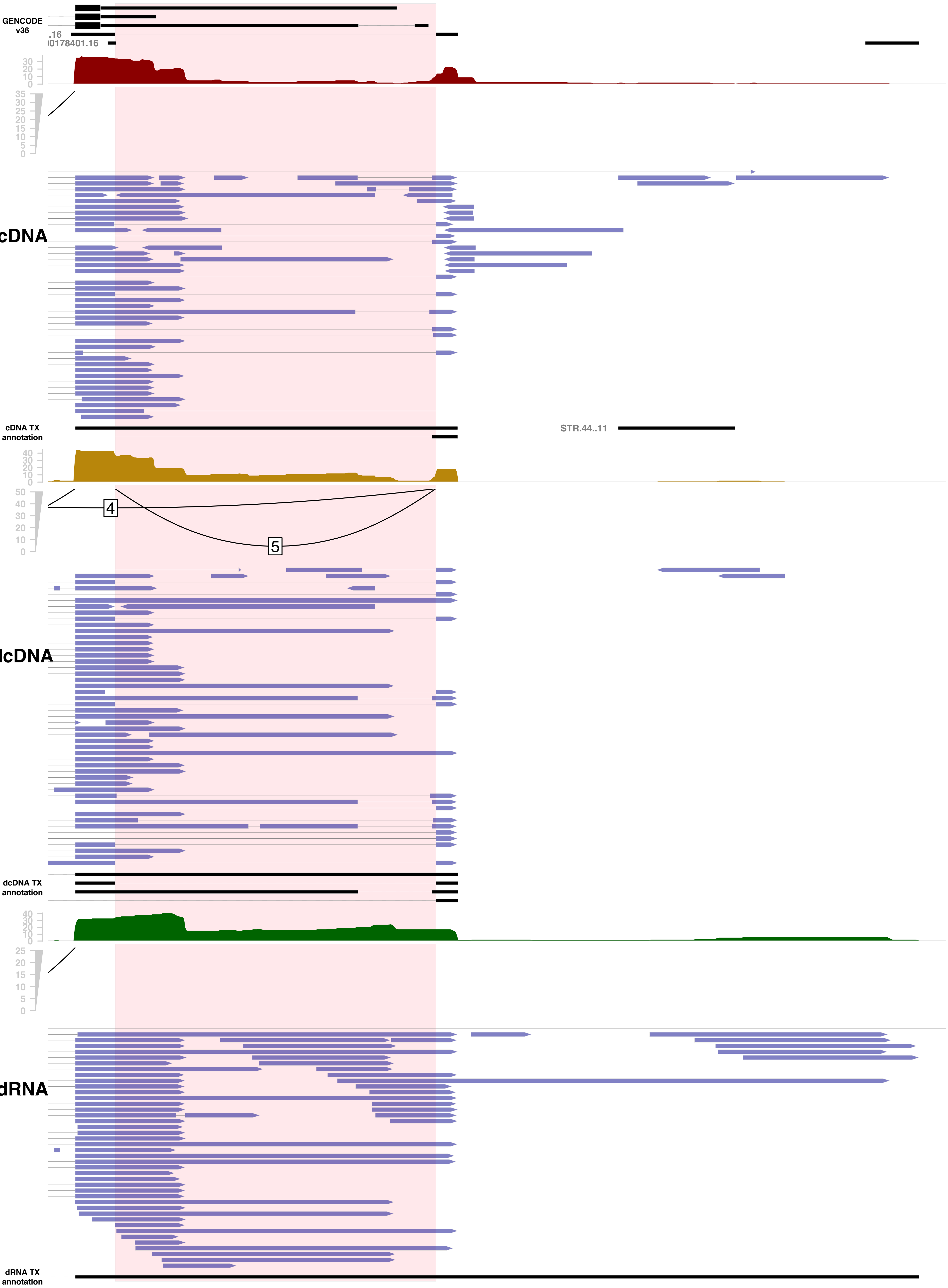

chr12:100626865−100627488:+ GAS2L3
Cell line: Hct116

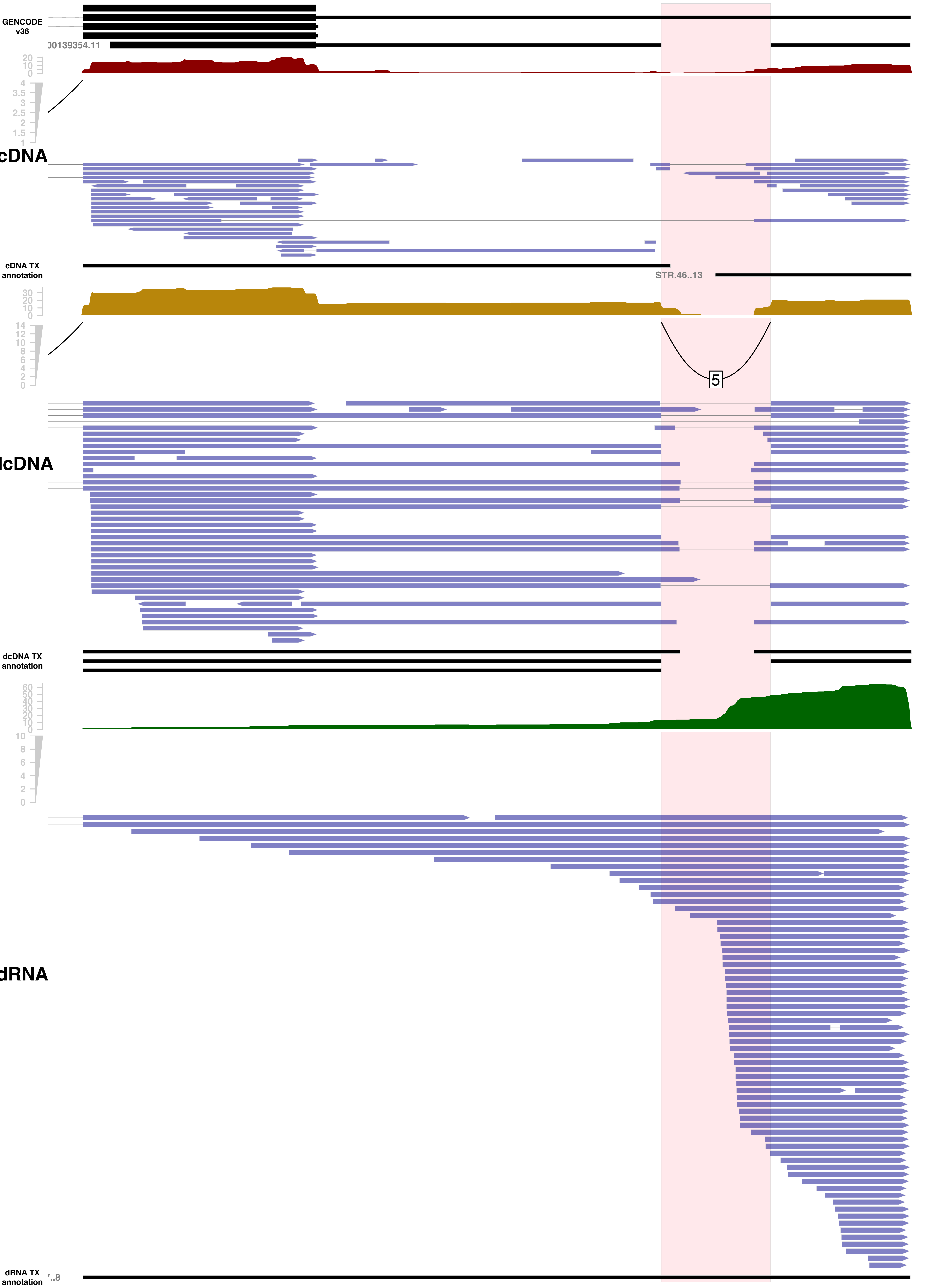

chr12:132721275−132721854:+ PGAM5
Cell line: Hct116
Direct repeat sequence: CTTG

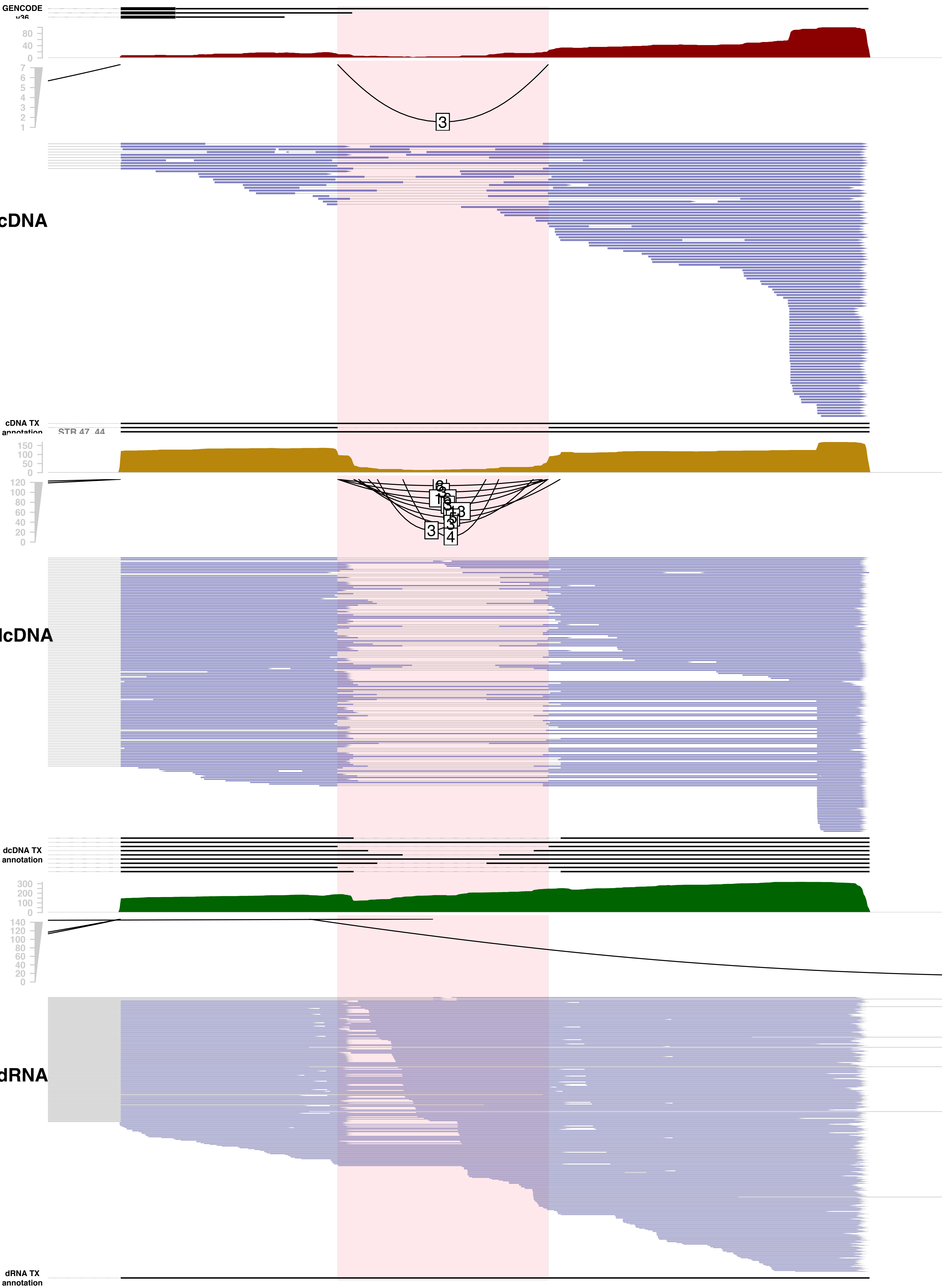

chr13:21374501−21374897:− ZDHHC20

Cell line: Hct116

Direct repeat sequence: CAGC

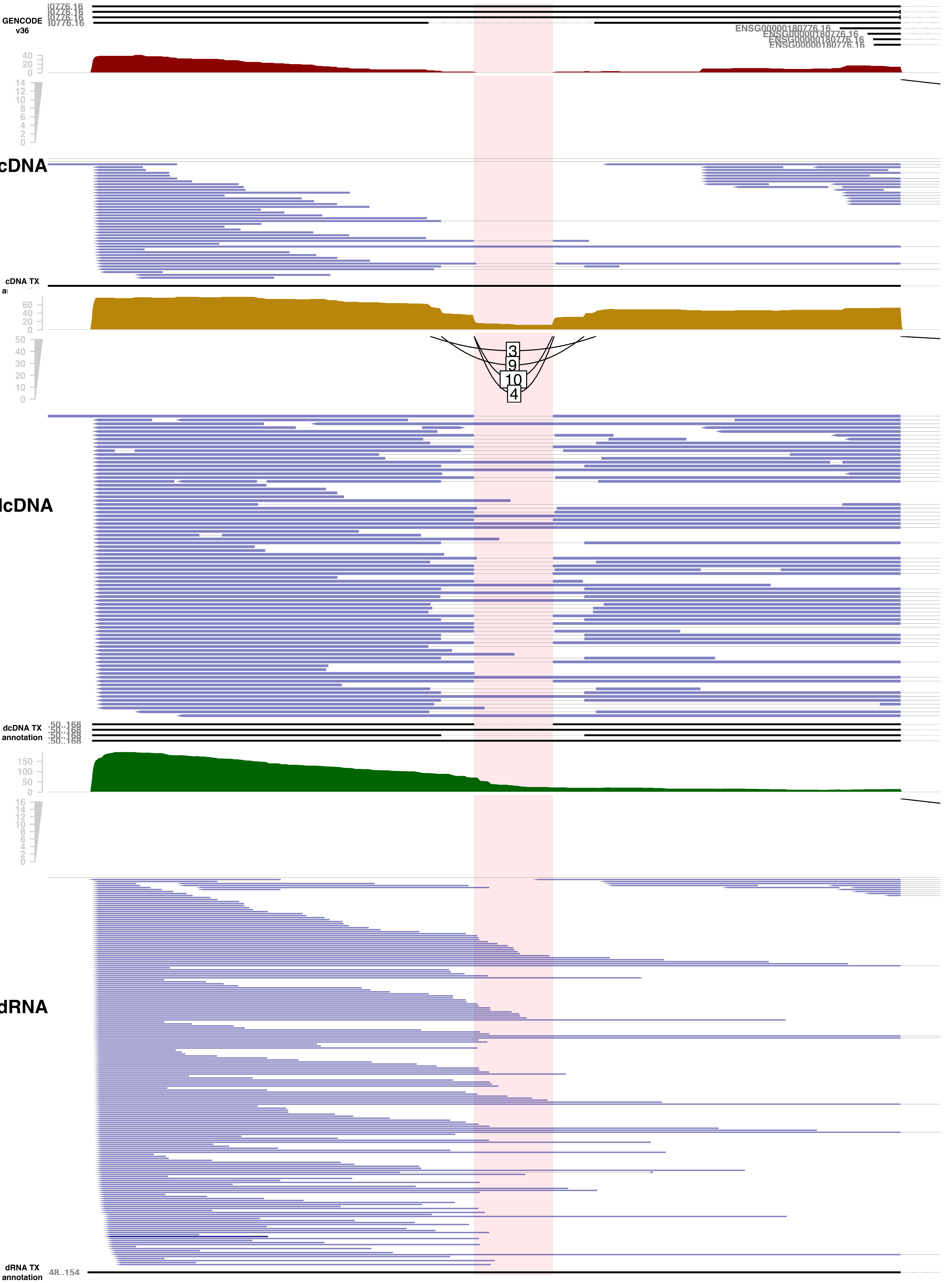

chr14:21460346−21460977:− RAB2B
Cell line: Hct116
Direct repeat sequence: CCTCC

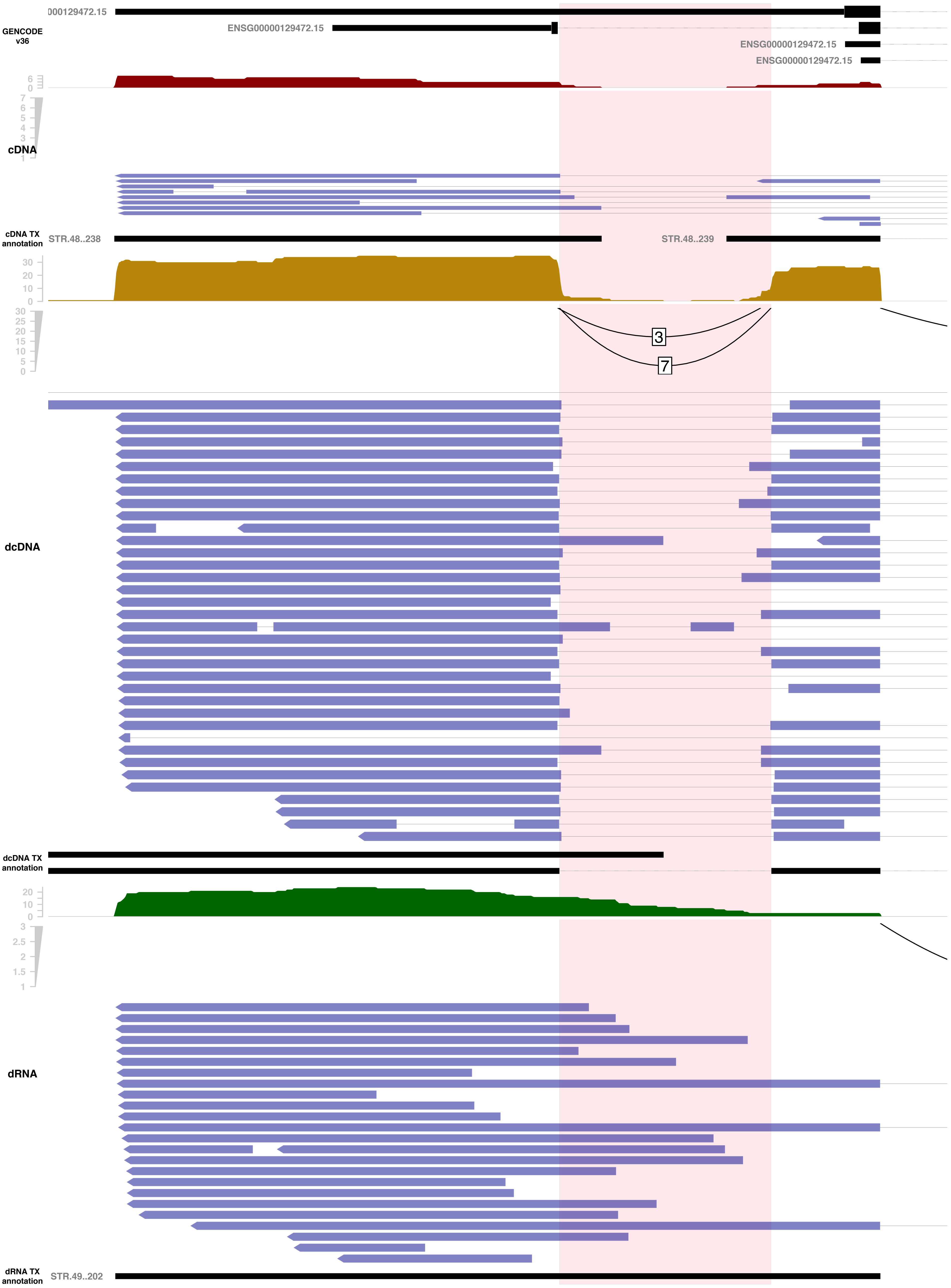

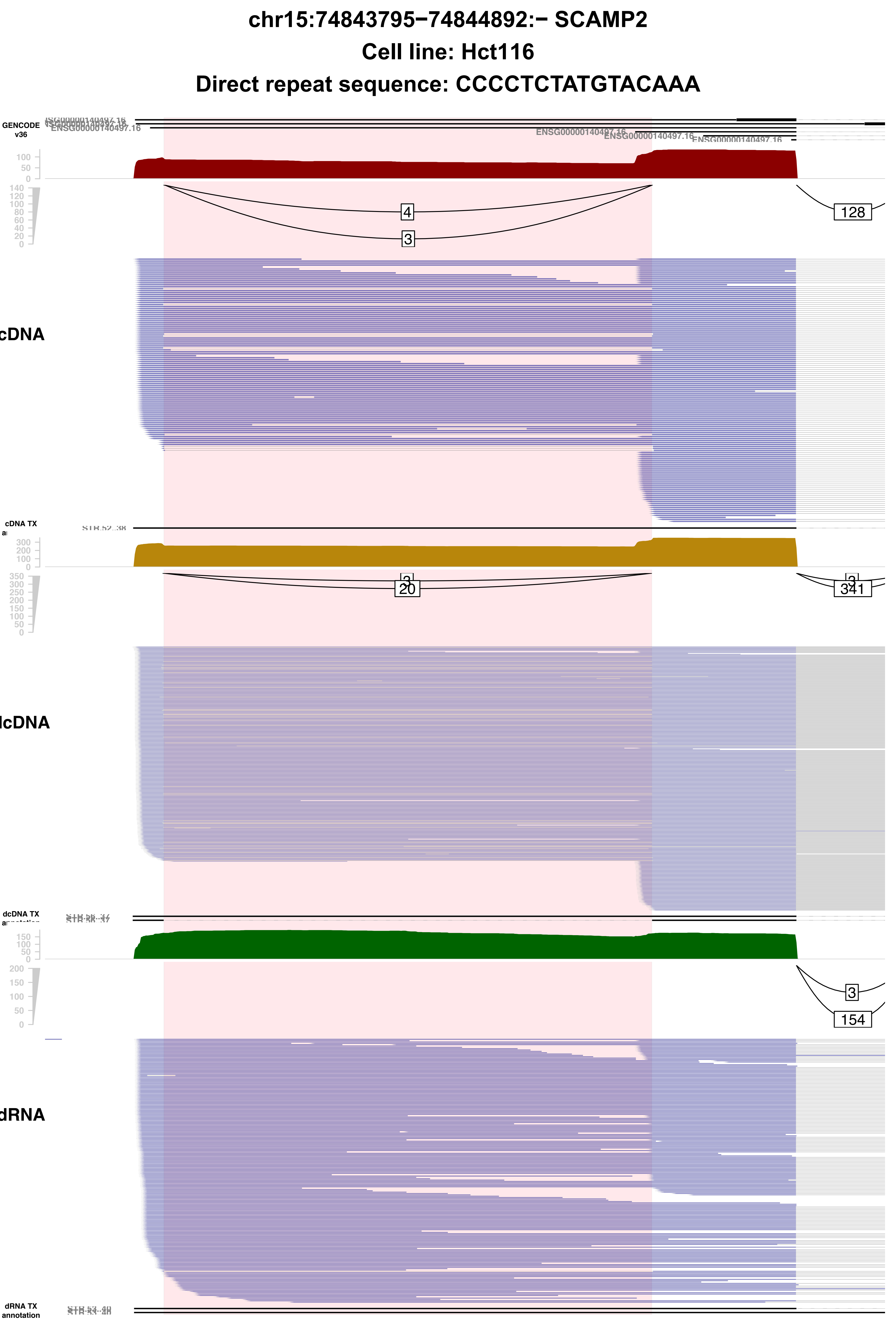
chr15:74843795−74844892:− SCAMP2
Cell line: Hct116
Direct repeat sequence: CCCCTCTATGTACAAA
GENCODE v36
ENSG00000140497.16
cDNA
cDNA TX
dcDNA
dcDNA TX
dRNA
dRNA TX annotation
4
3
128
20
341
154

chr17:3663567−3663637:− TAX1BP3,P2RX5−TAX1BP3
Cell line: Hct116

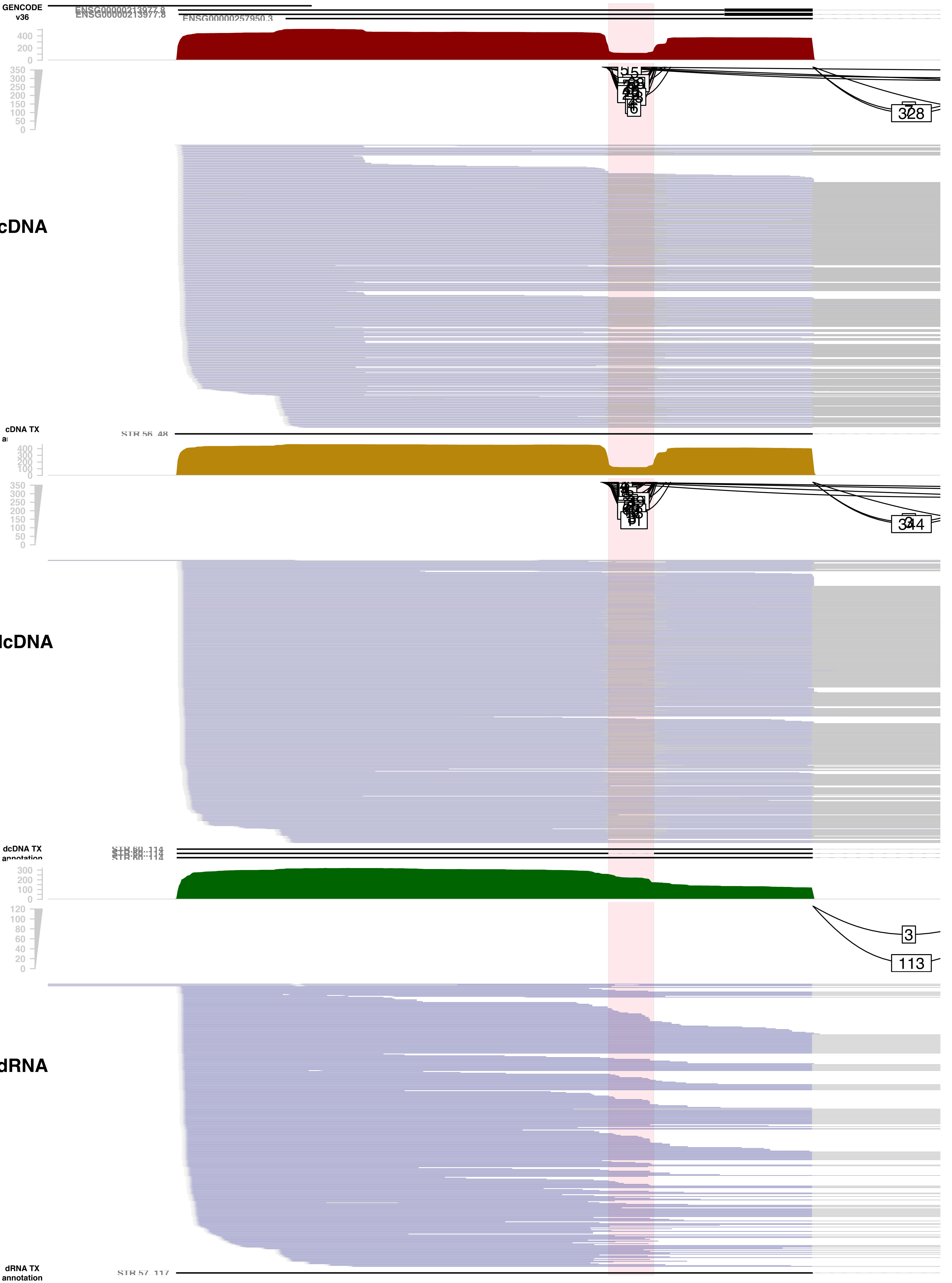

chr1:220057918−220058829:− BPNT1
Cell line: Hct116

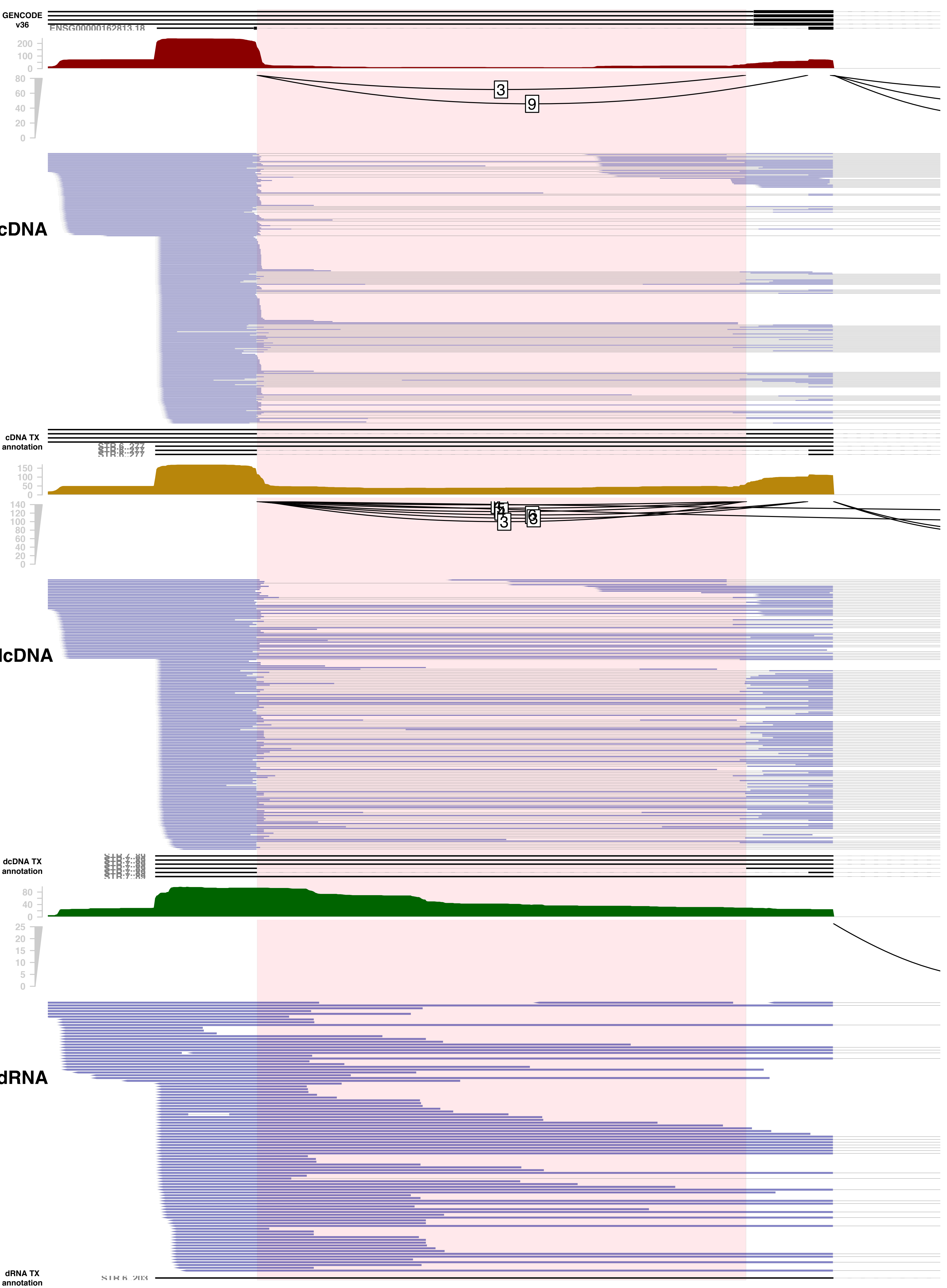

chr17:47118517−47118594:− CDC27
Cell line: Hct116

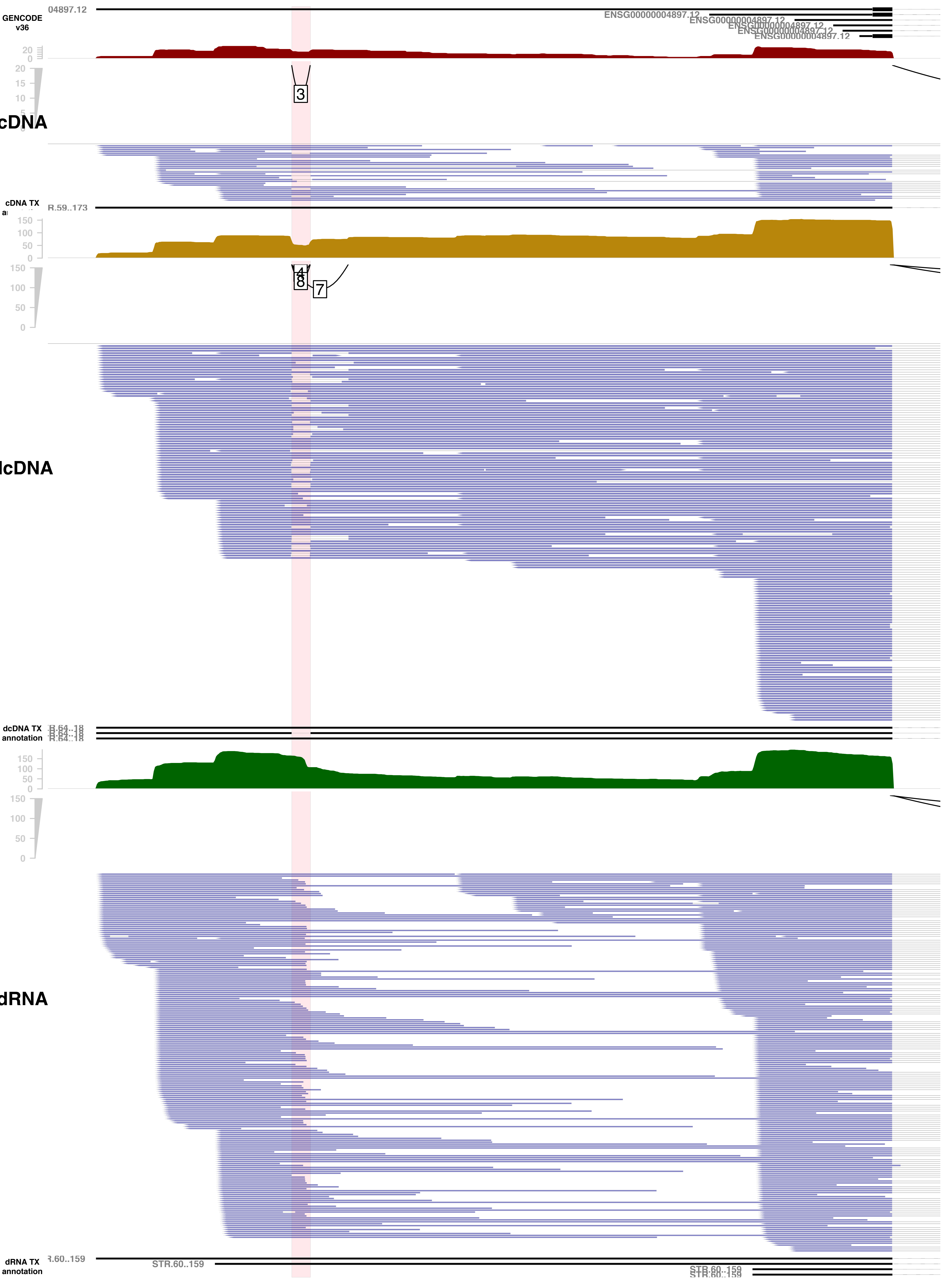

chr17:57680745−57680893:+ MSI2
Cell line: Hct116
Direct repeat sequence: CCTG

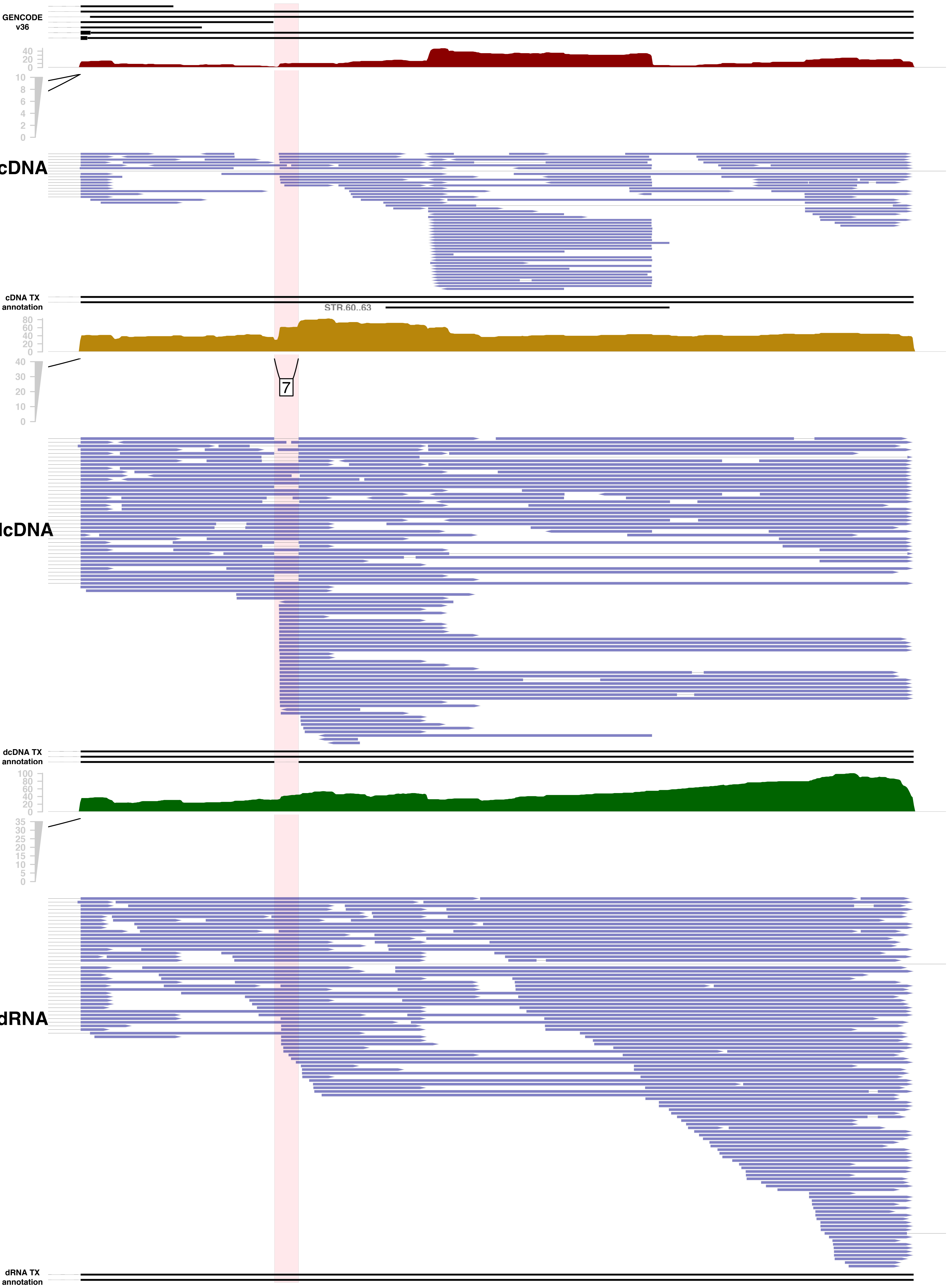

chr20:45883263−45884986:+ ZSWIM1

Cell line: Hct116

Direct repeat sequence: CTCCAGCC

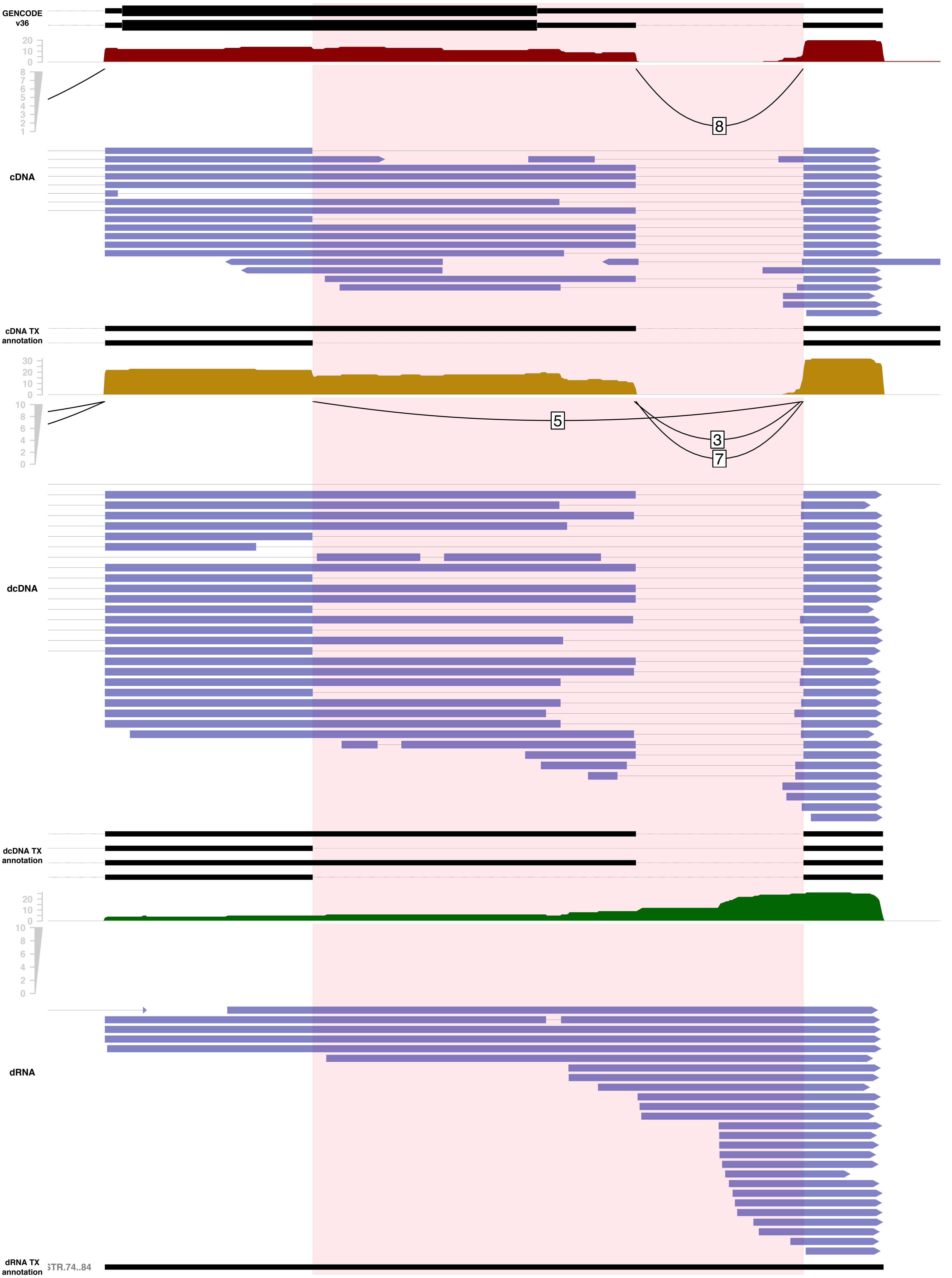

chr1:8012958−8013358:− ERRFI1

Cell line: HepG2

Direct repeat sequence: GGGAAGC

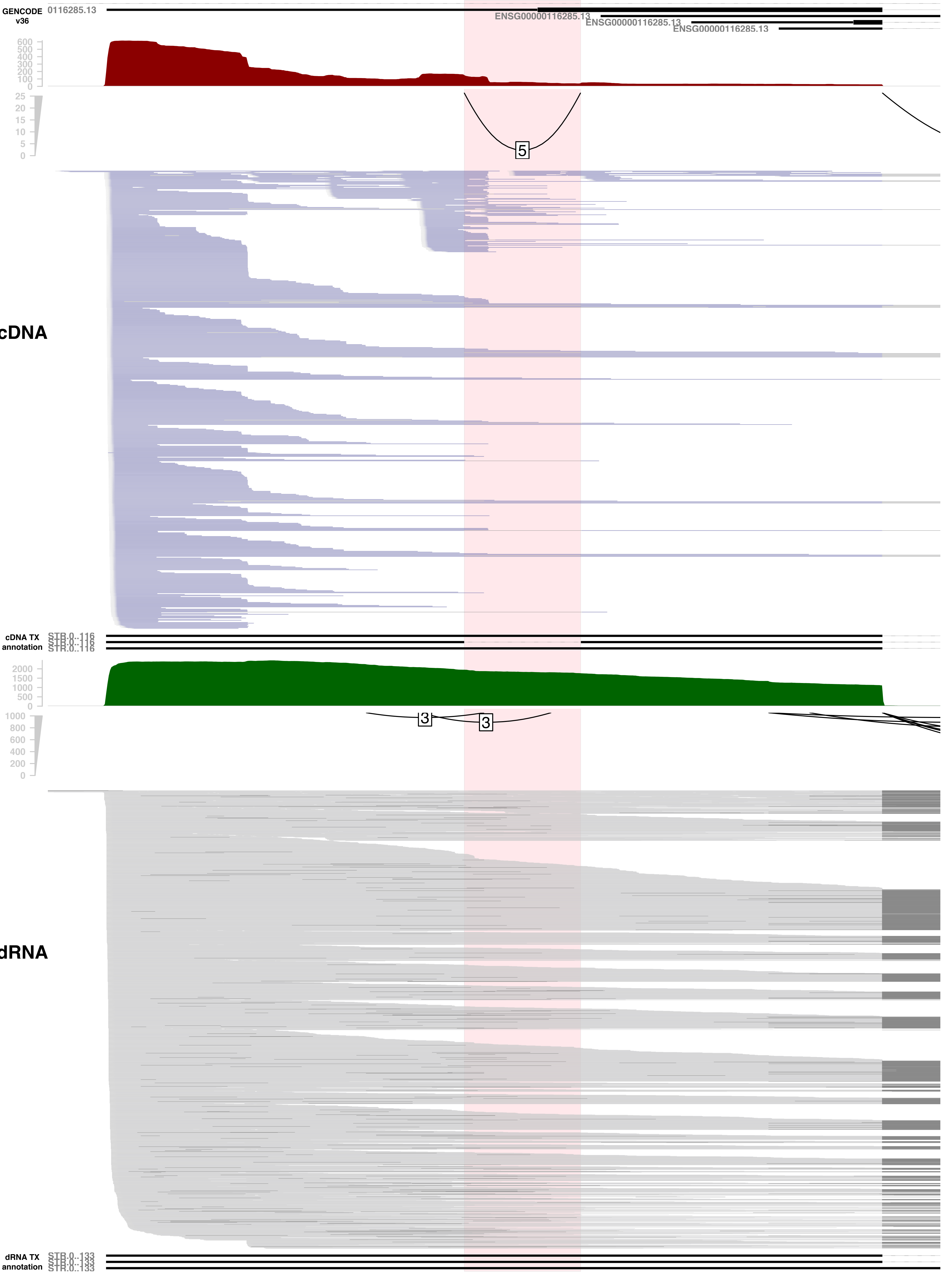

chrM:8766−9064:+
Cell line: HepG2

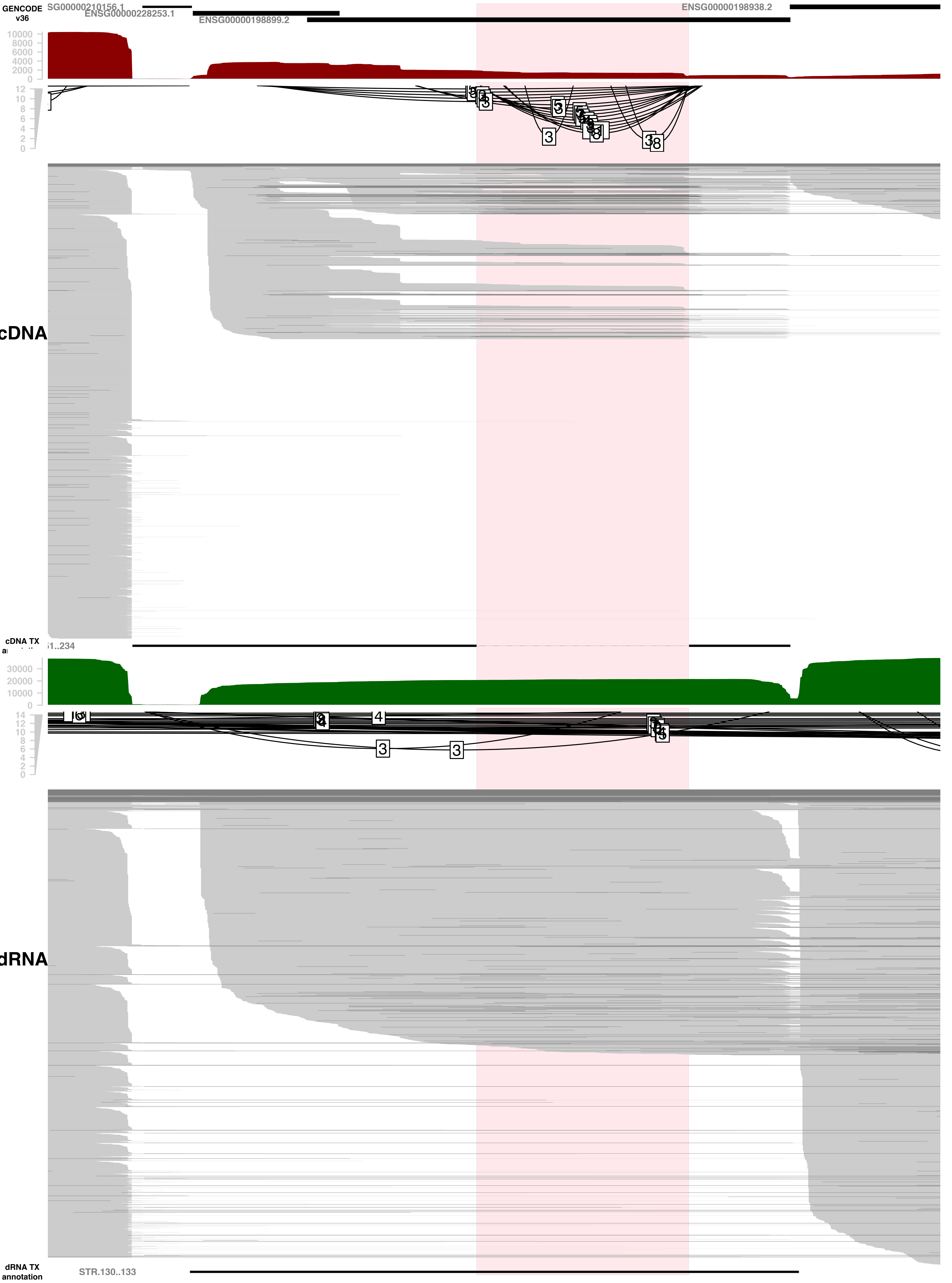

chr5:163437659−163437758:+ CCNG1
Cell line: HepG2

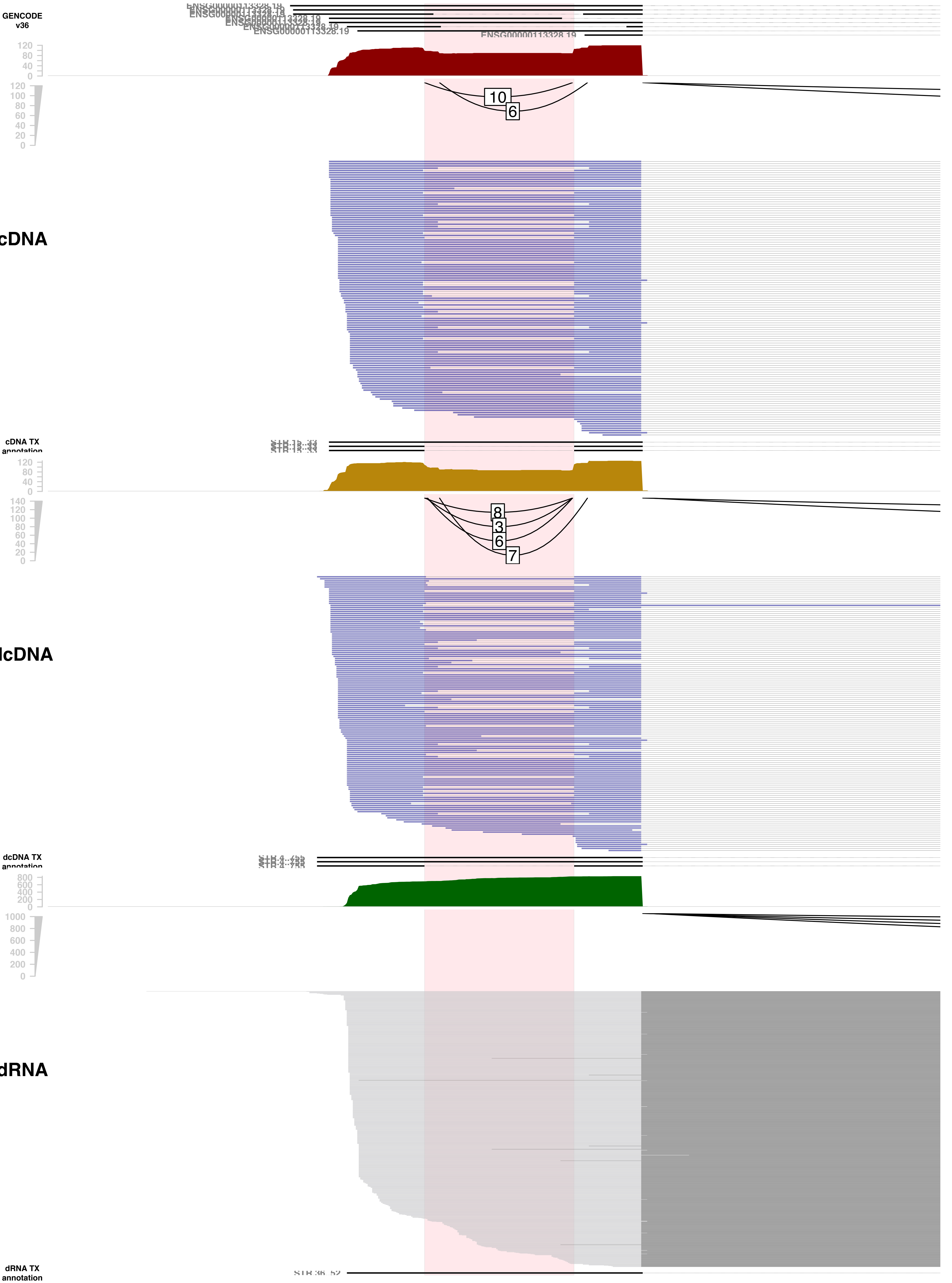

chr12:75501816−75501921:+ GLIPR1
Cell line: HepG2

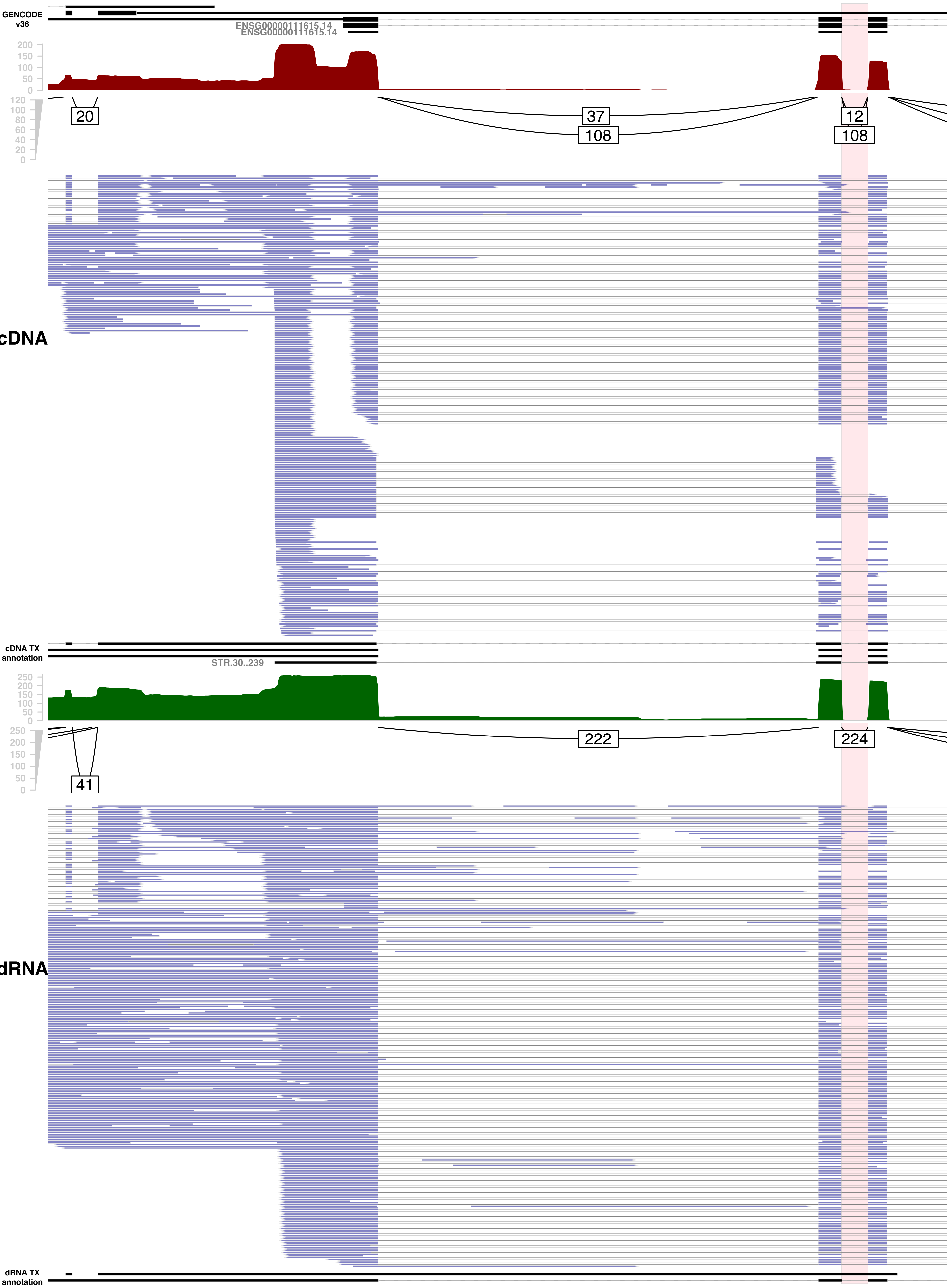

chr14:31447249−31447389:− RP11−176H8.1,DTD2

Cell line: HepG2

Direct repeat sequence: CAGCAC

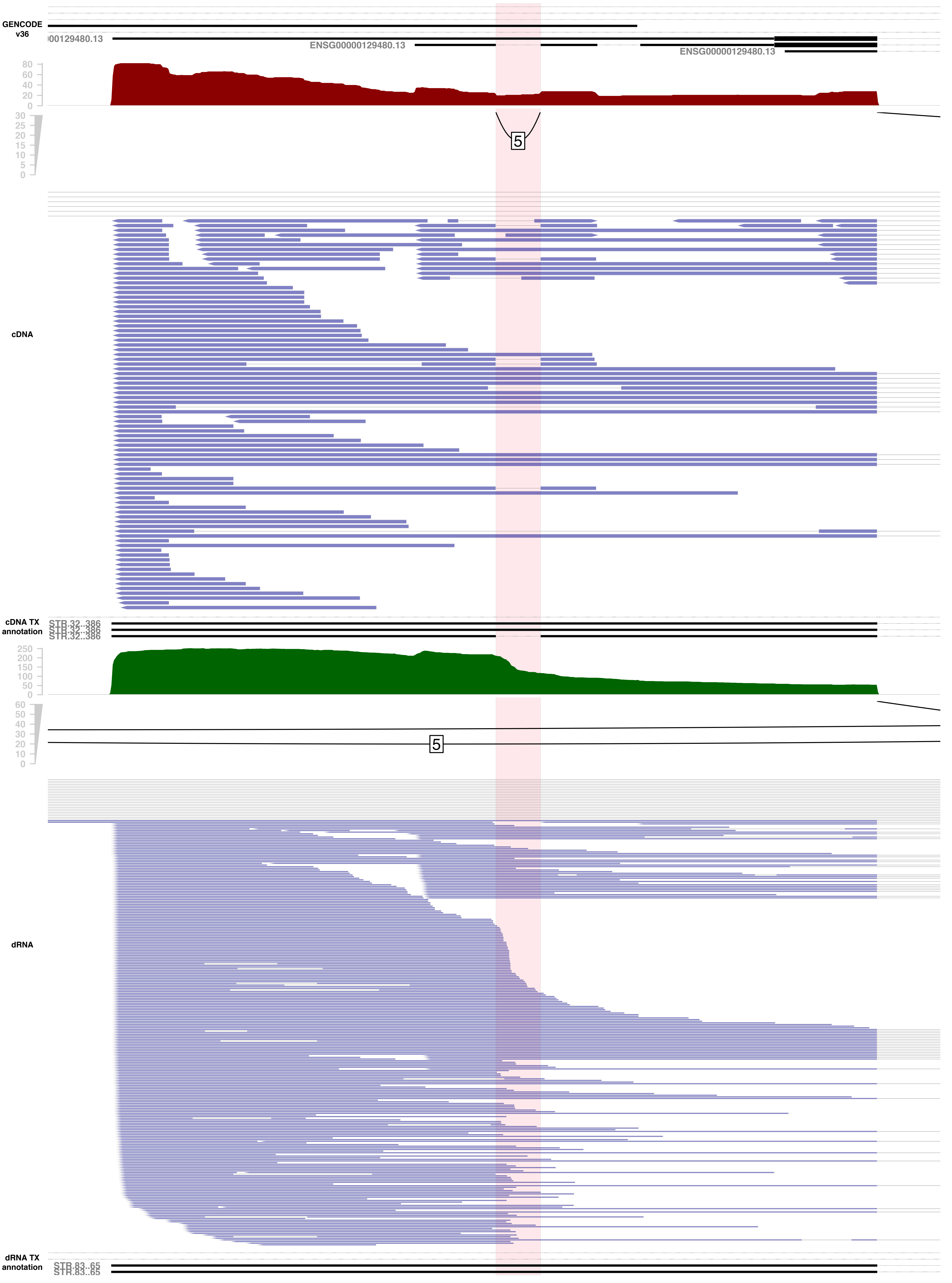

chr17:3663563−3663657:− TAX1BP3,P2RX5−TAX1BP3
Cell line: HepG2
Direct repeat sequence: GACCG

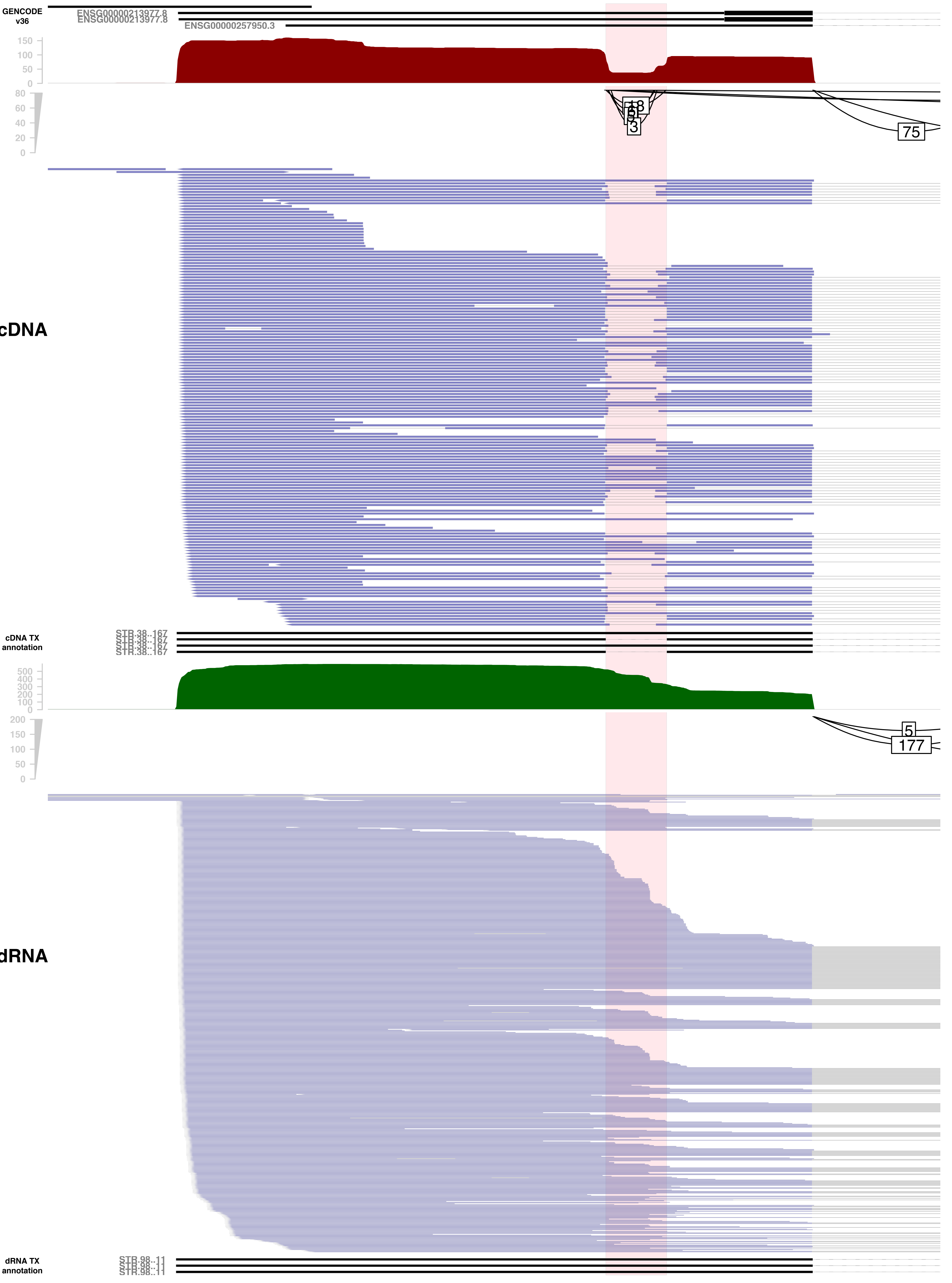

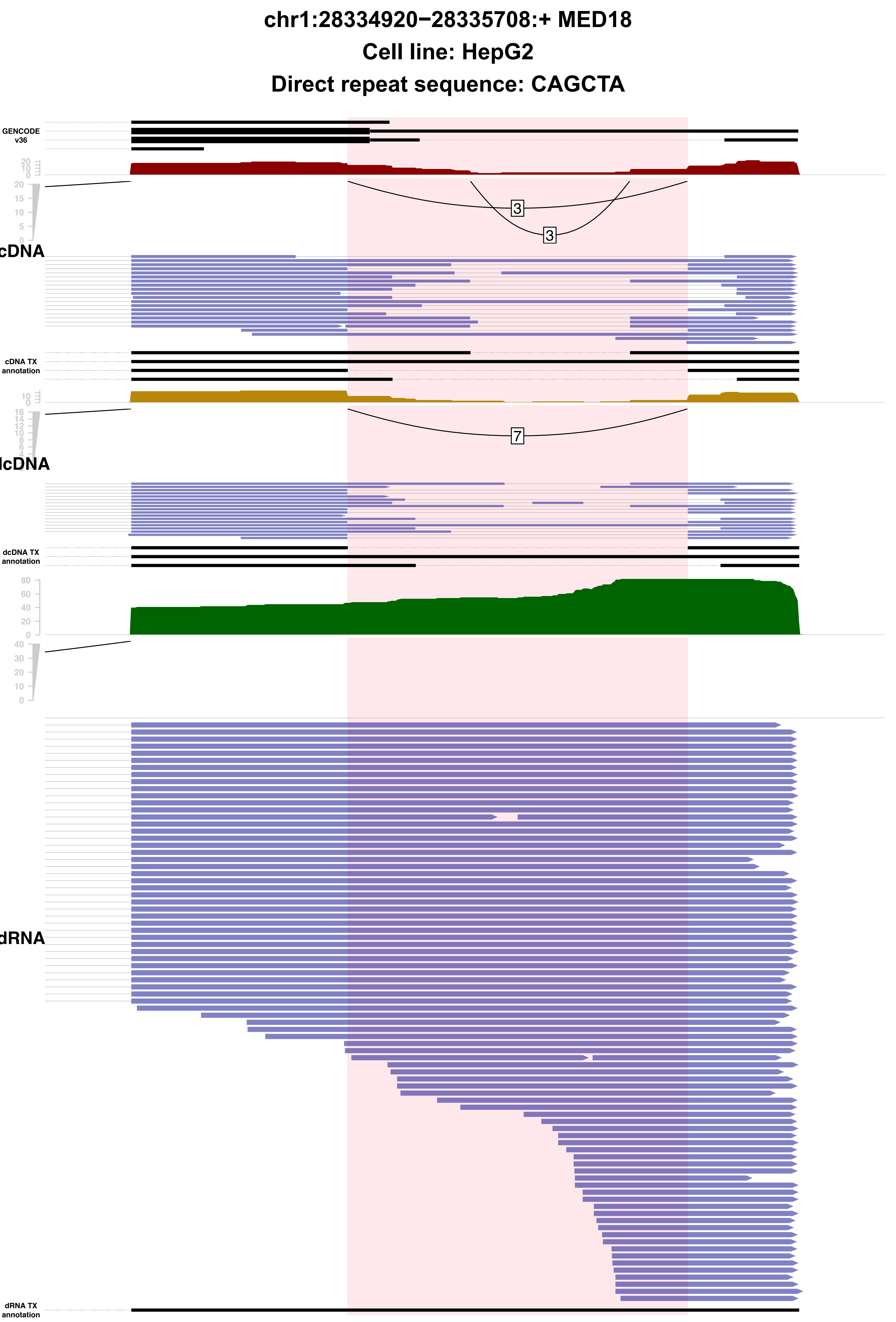

chr1:28334920−28335708:+ MED18
Cell line: HepG2
Direct repeat sequence: CAGCTA
GENCODE v36
cDNA
cDNA TX annotation
dcDNA
dcDNA TX annotation
dRNA
dRNA TX annotation
3
3
7

chr1:28334920−28335708:+ MED18
Cell line: K562
Direct repeat sequence: CAGCTA

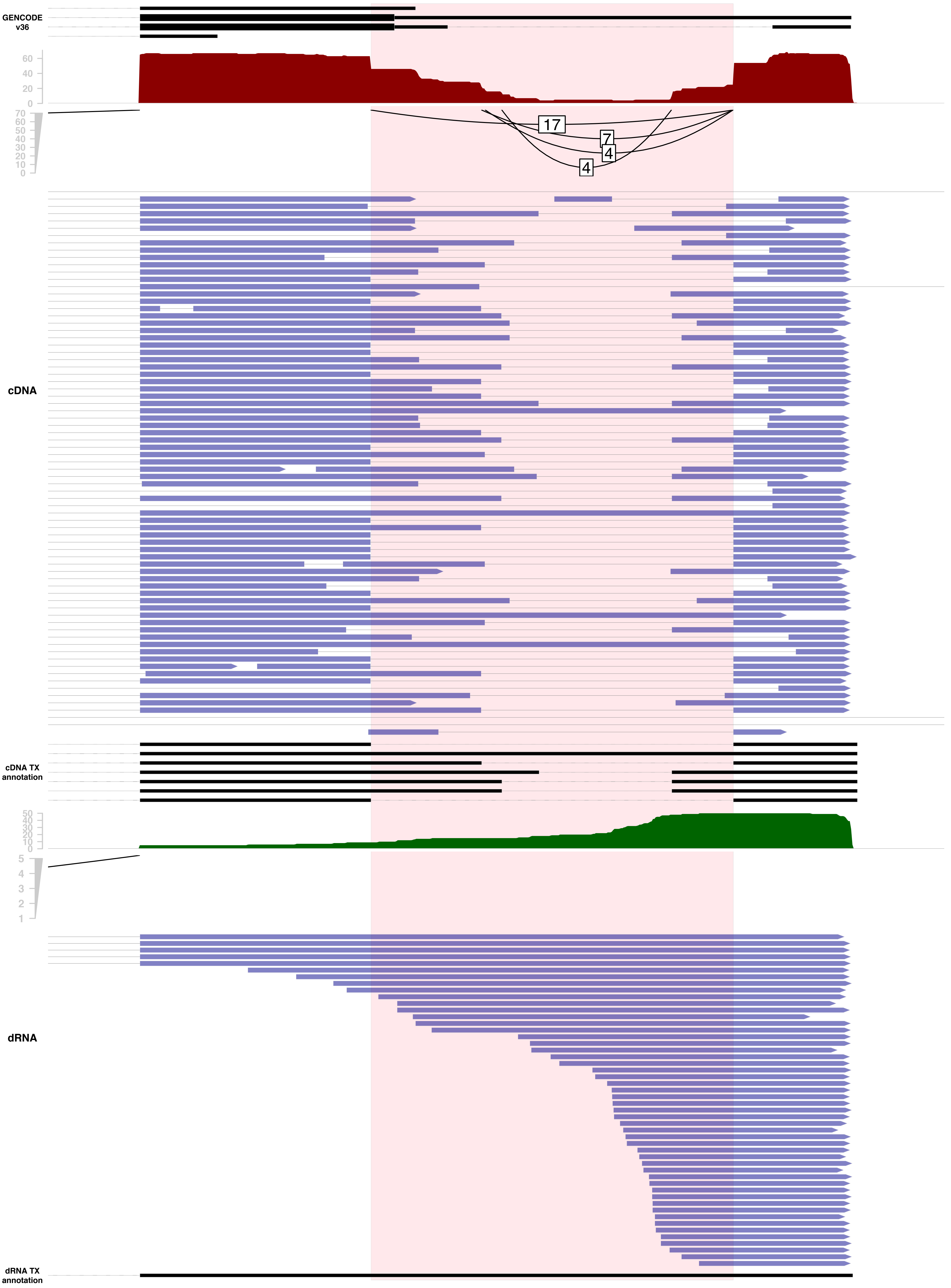

chr1:28335161−28335708:+ MED18
Cell line: K562
Direct repeat sequence: CAGC

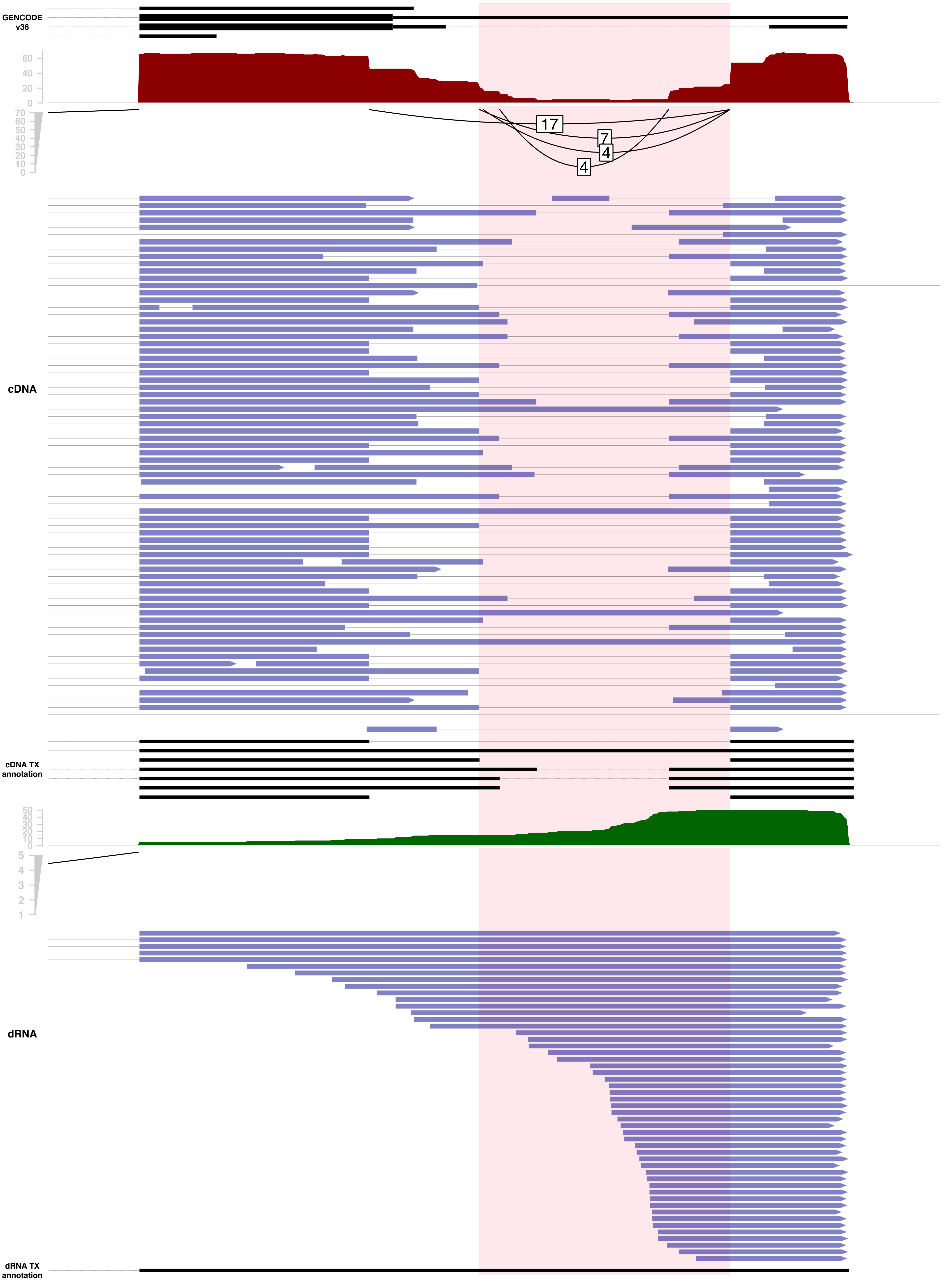

chr5:163437659−163437758:+ CCNG1

Cell line: K562

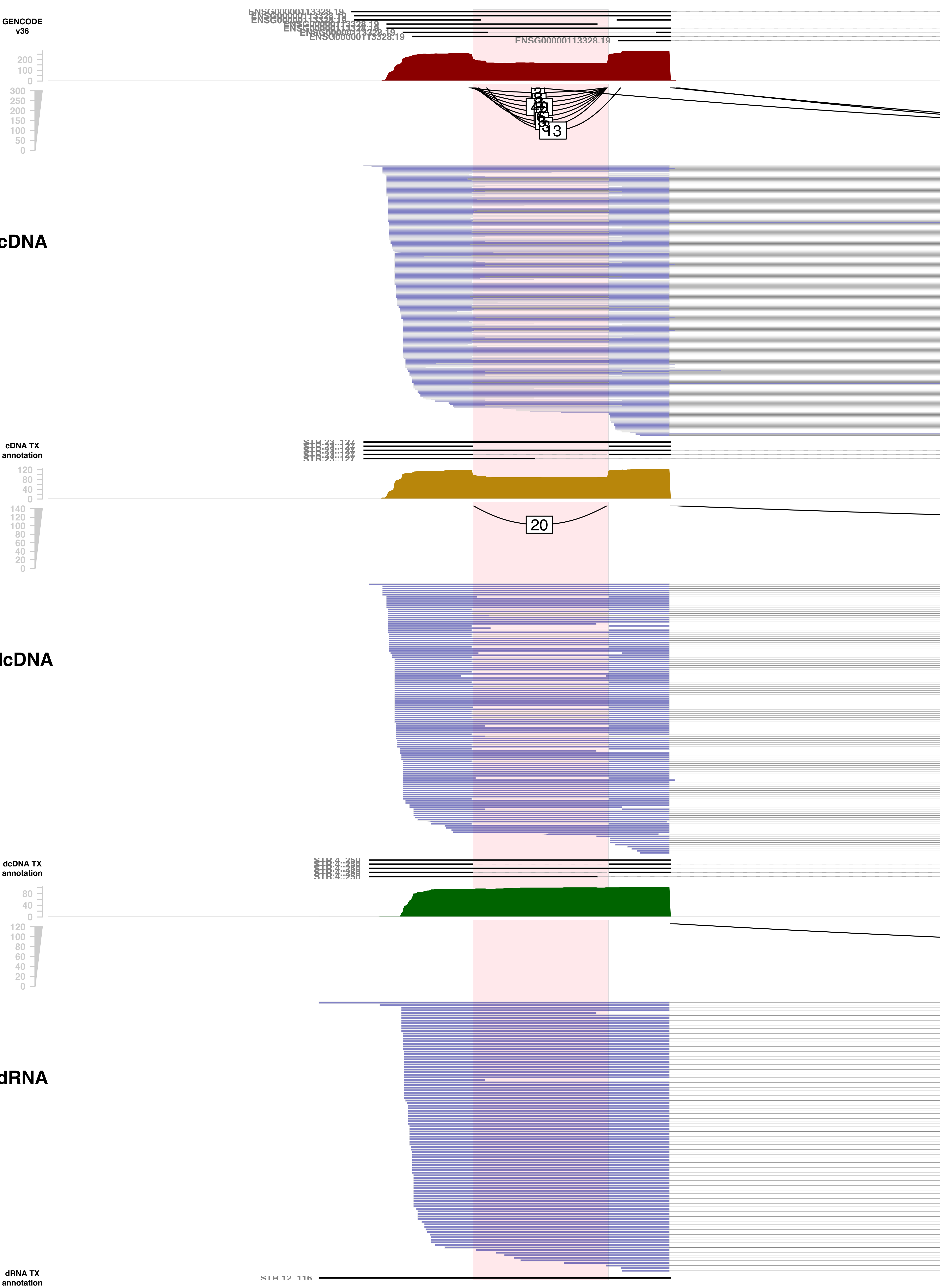

chr6:170584411−170584491:− PDCD2
Cell line: K562

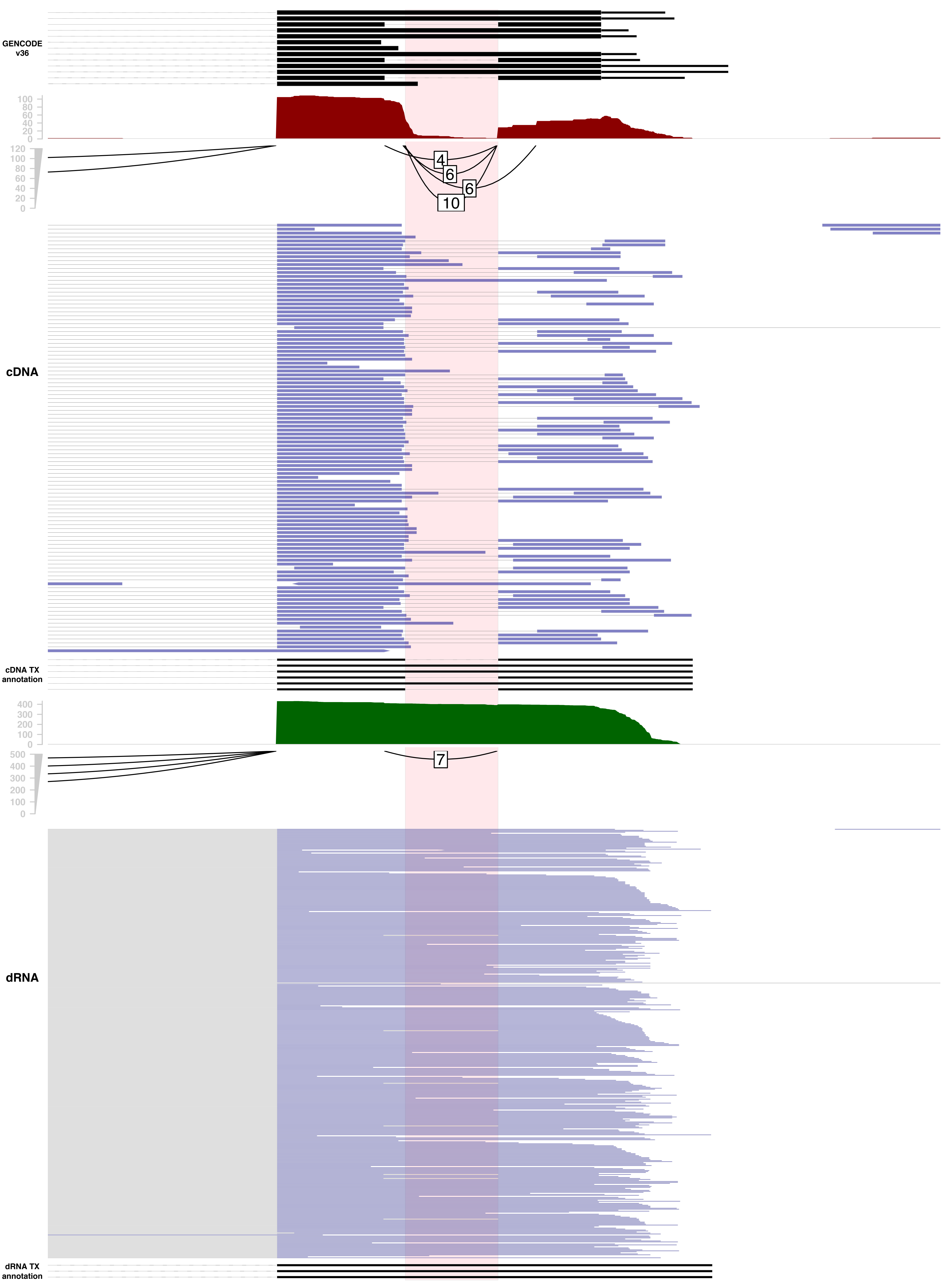

chr8:47976979−47977736:+ MCM4
Cell line: K562

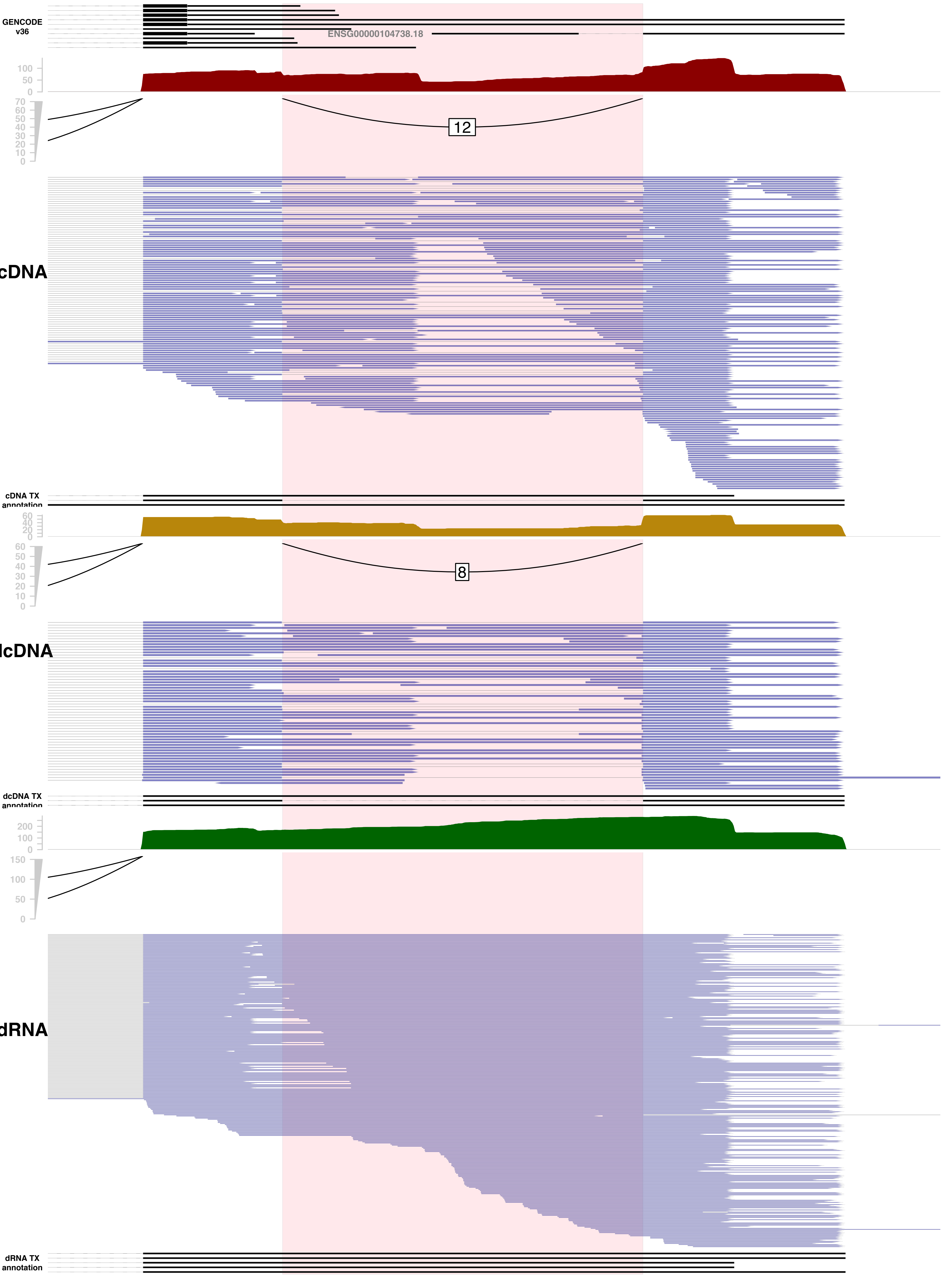

chr9:127908169−127908317:− ST6GALNAC4
Cell line: K562
Direct repeat sequence: CTCCAC

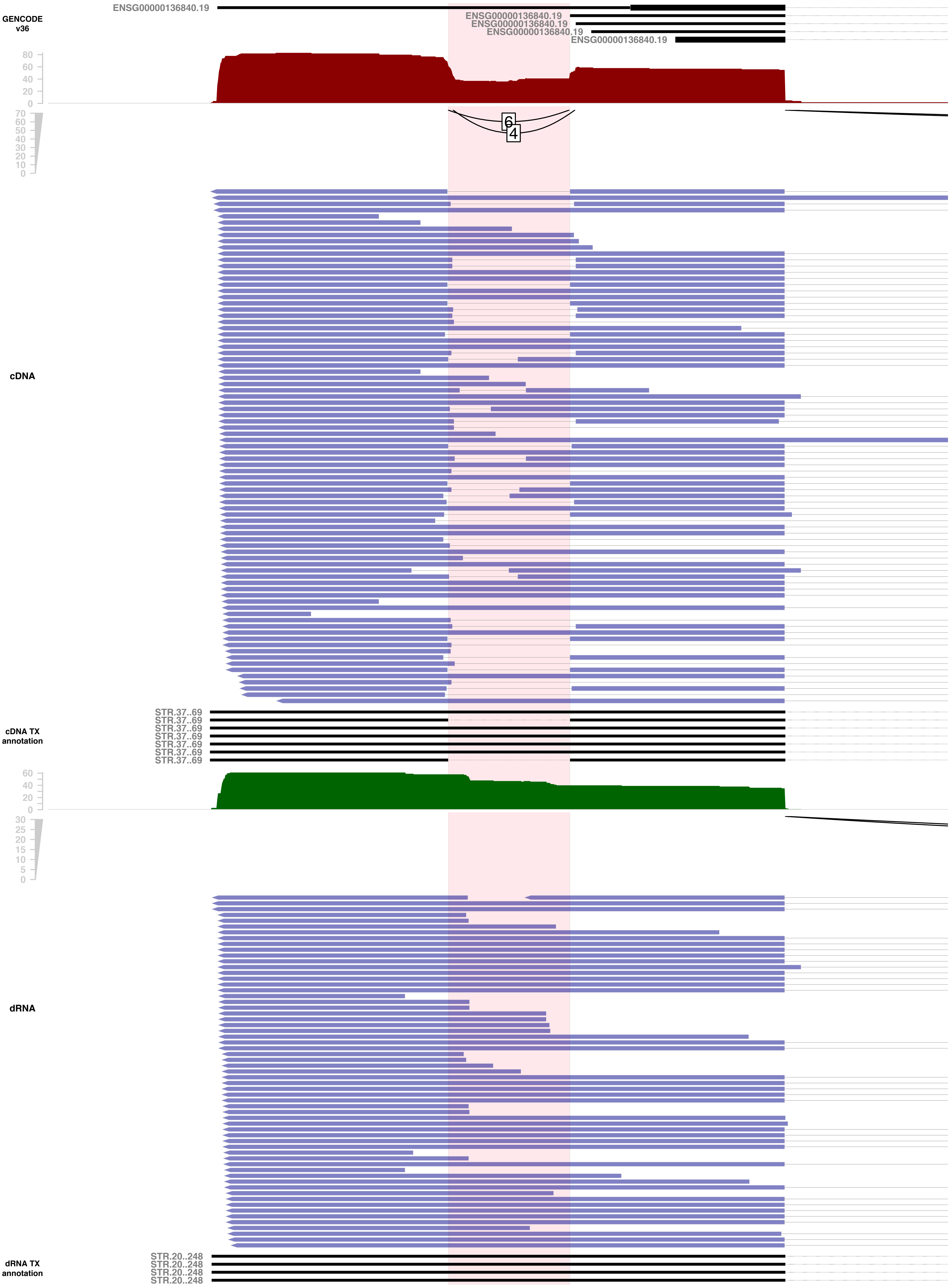

chr11:6704862−6705515:+
Cell line: K562

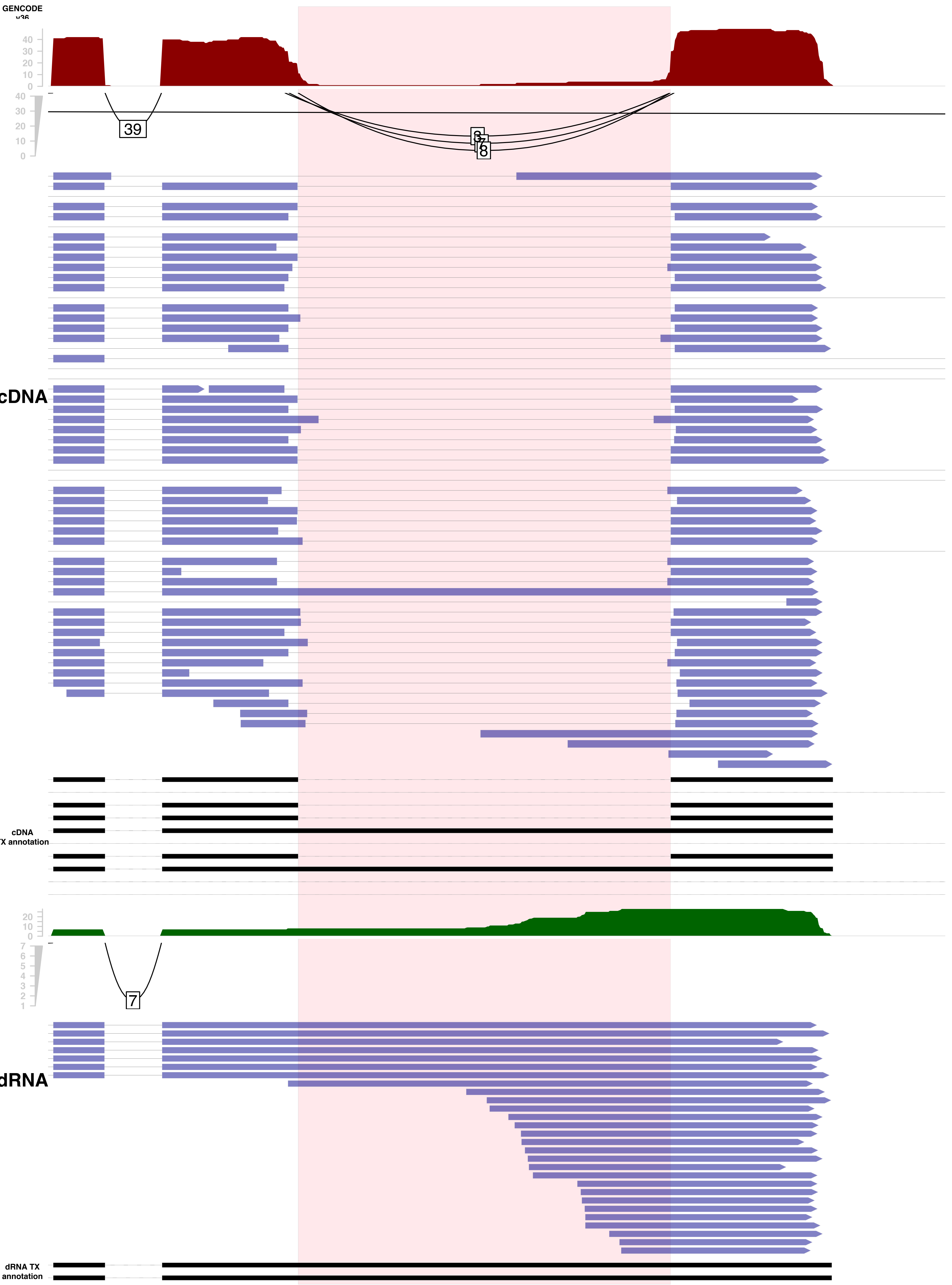

chr12:62965697−62965963:+ RPL14P1
Cell line: K562
Direct repeat sequence: CAAGAA

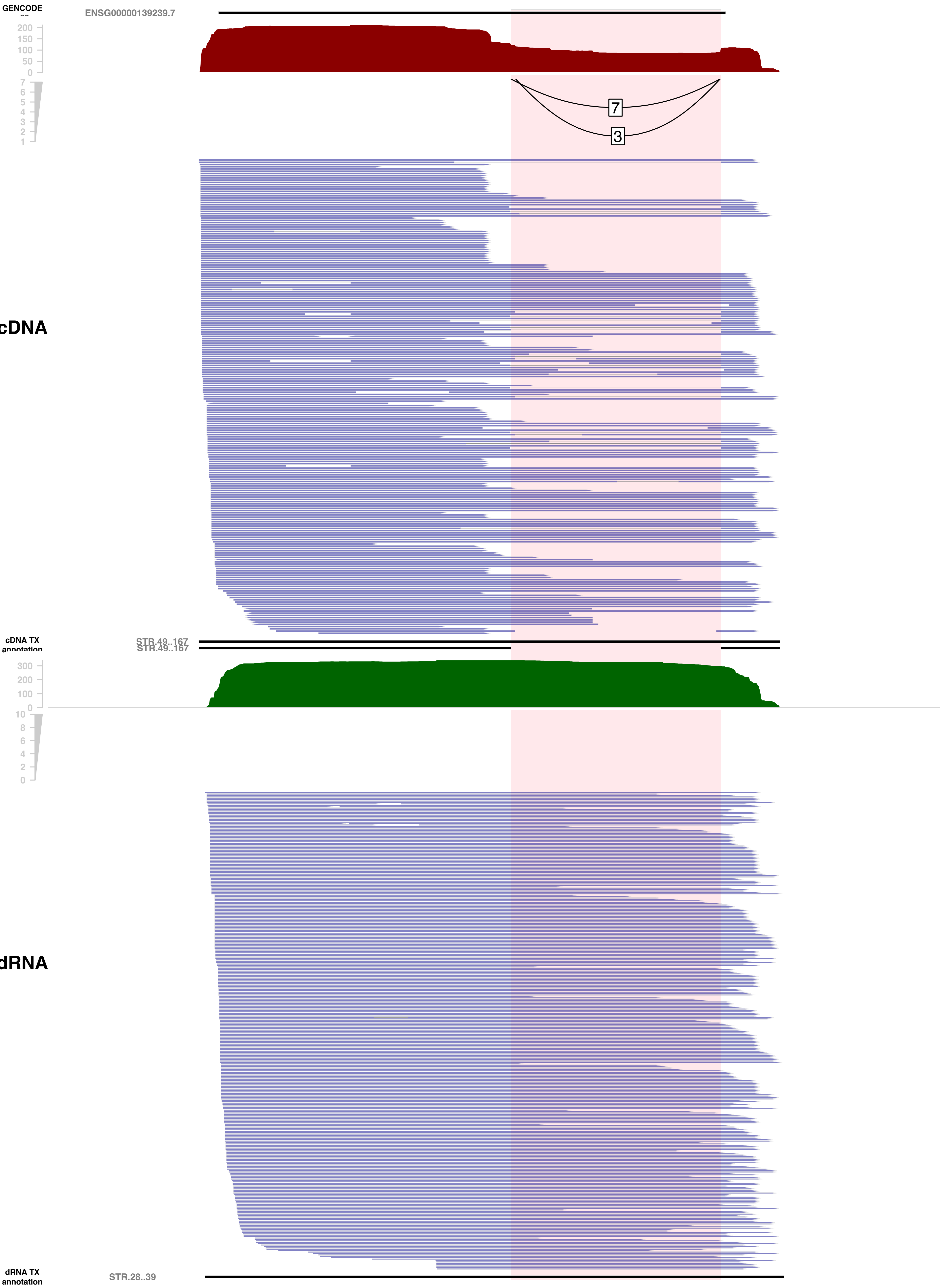

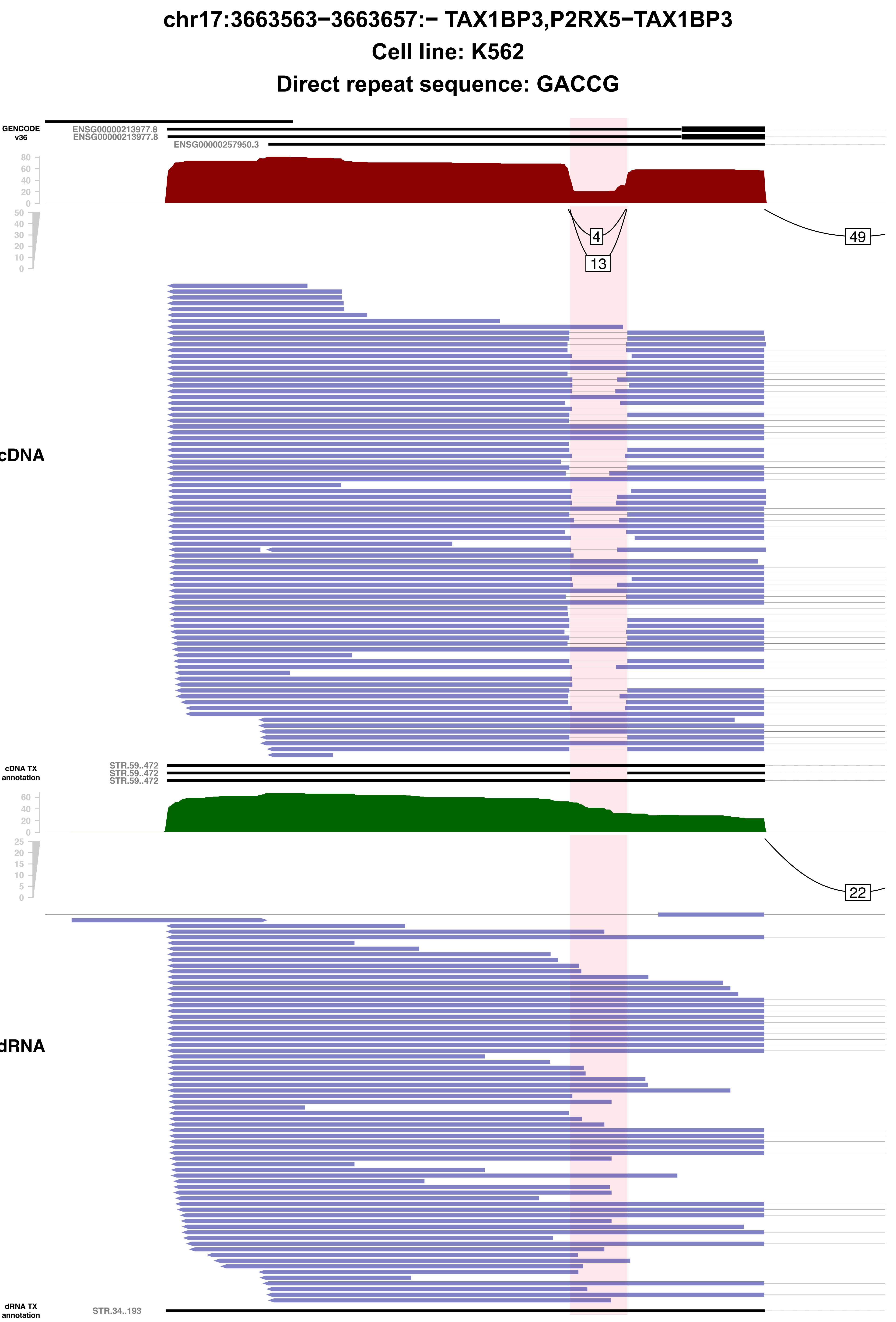
chr17:3663563−3663657:− TAX1BP3,P2RX5−TAX1BP3
Cell line: K562
Direct repeat sequence: GACCG
GENCODE v36
ENSG00000213977.8
ENSG00000213977.8
ENSG00000257950.3
4
13
49
cDNA
cDNA TX annotation
STR.59..472
STR.59..472
STR.59..472
22
dRNA
dRNA TX annotation
STR.34..193

chr19:35746759−35746910:+ PSENEN,AC002398.9
Cell line: K562
Direct repeat sequence: CCTG

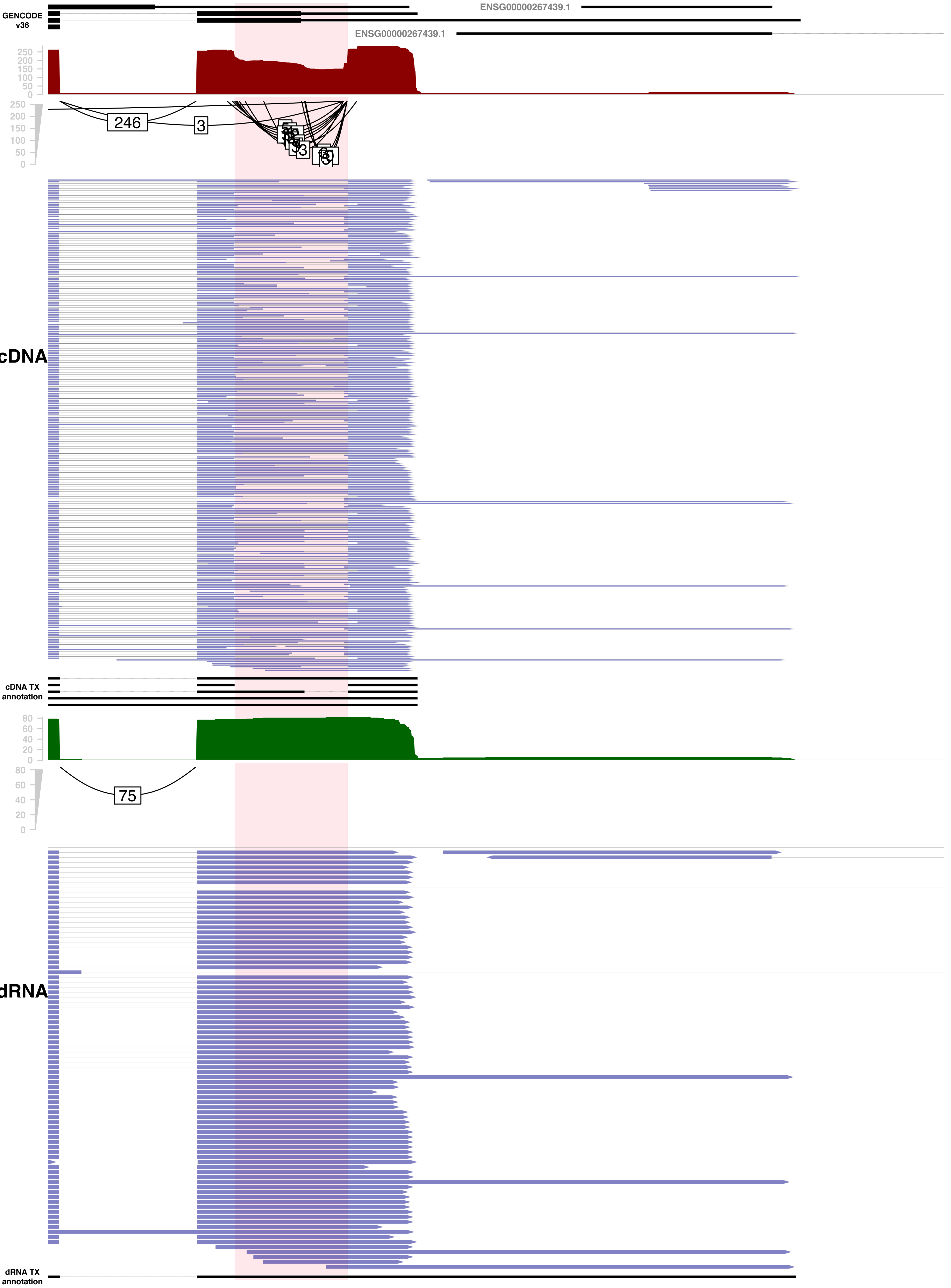

chr19:35746853−35746910:+ PSENEN,AC002398.9
Cell line: K562

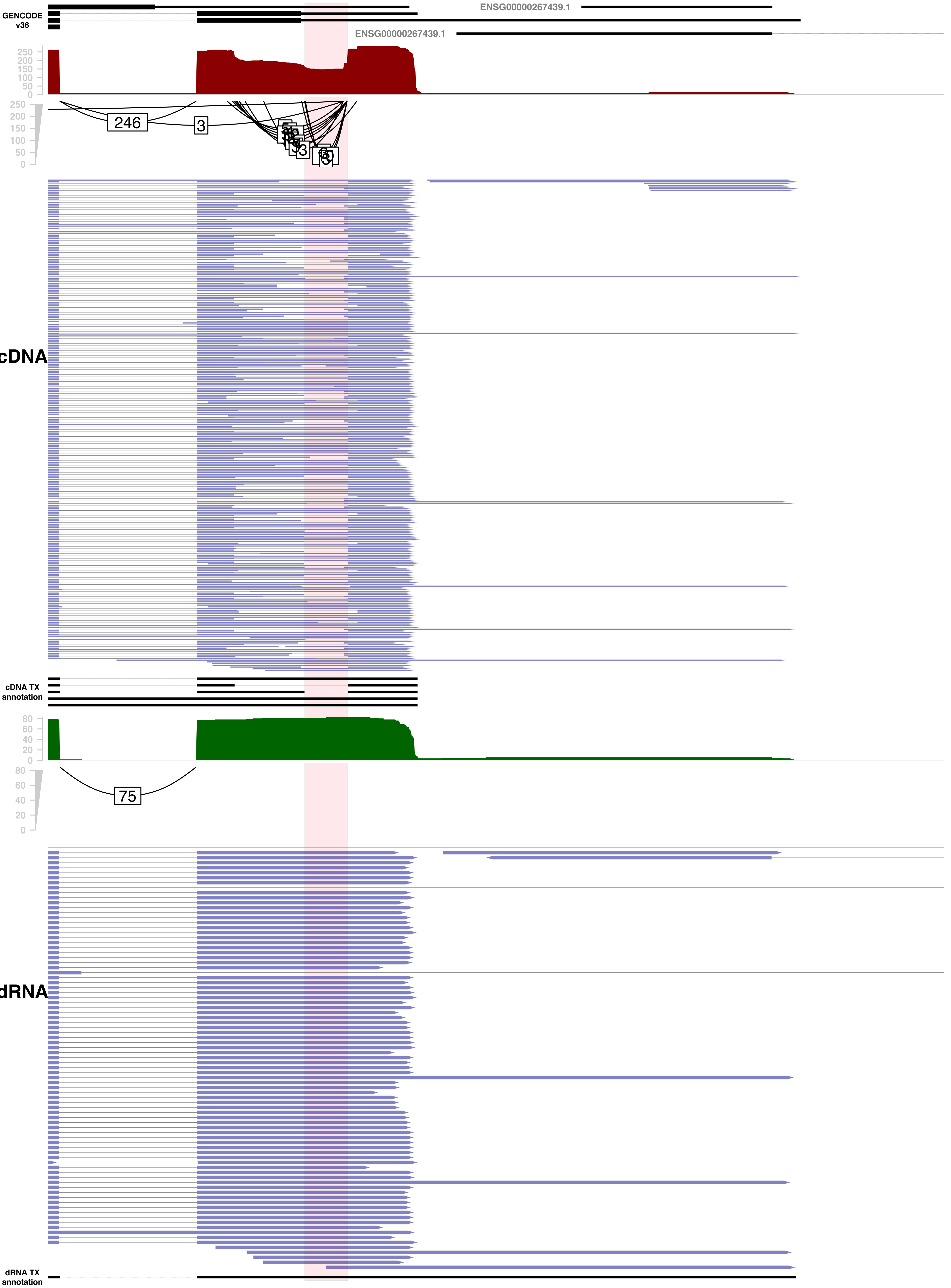

chr20:58891551−58891720:+ GNAS
Cell line: K562
Direct repeat sequence: CCGCC

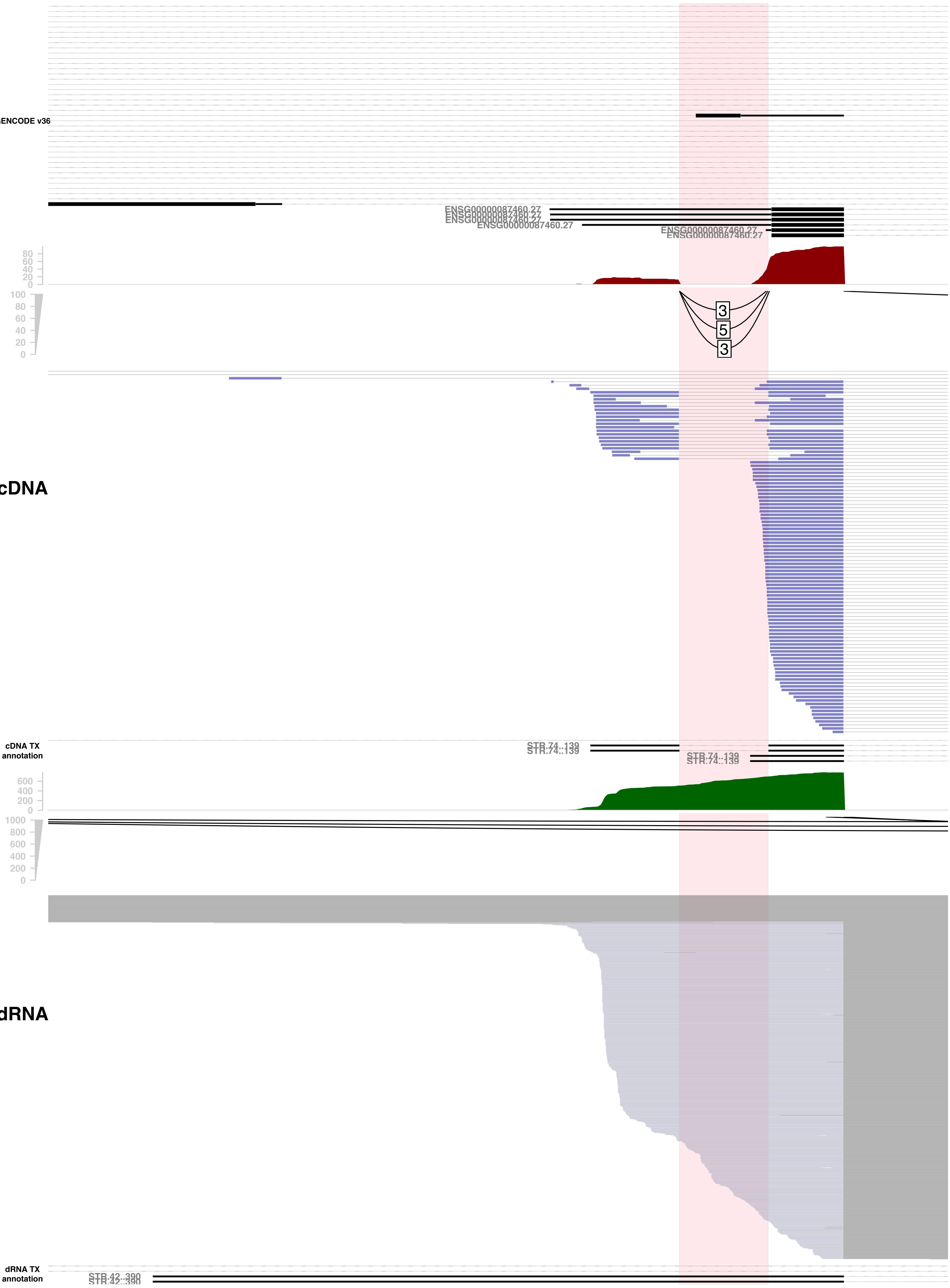

chr4:56459858−56460922:+ PAICS
Cell line: K562

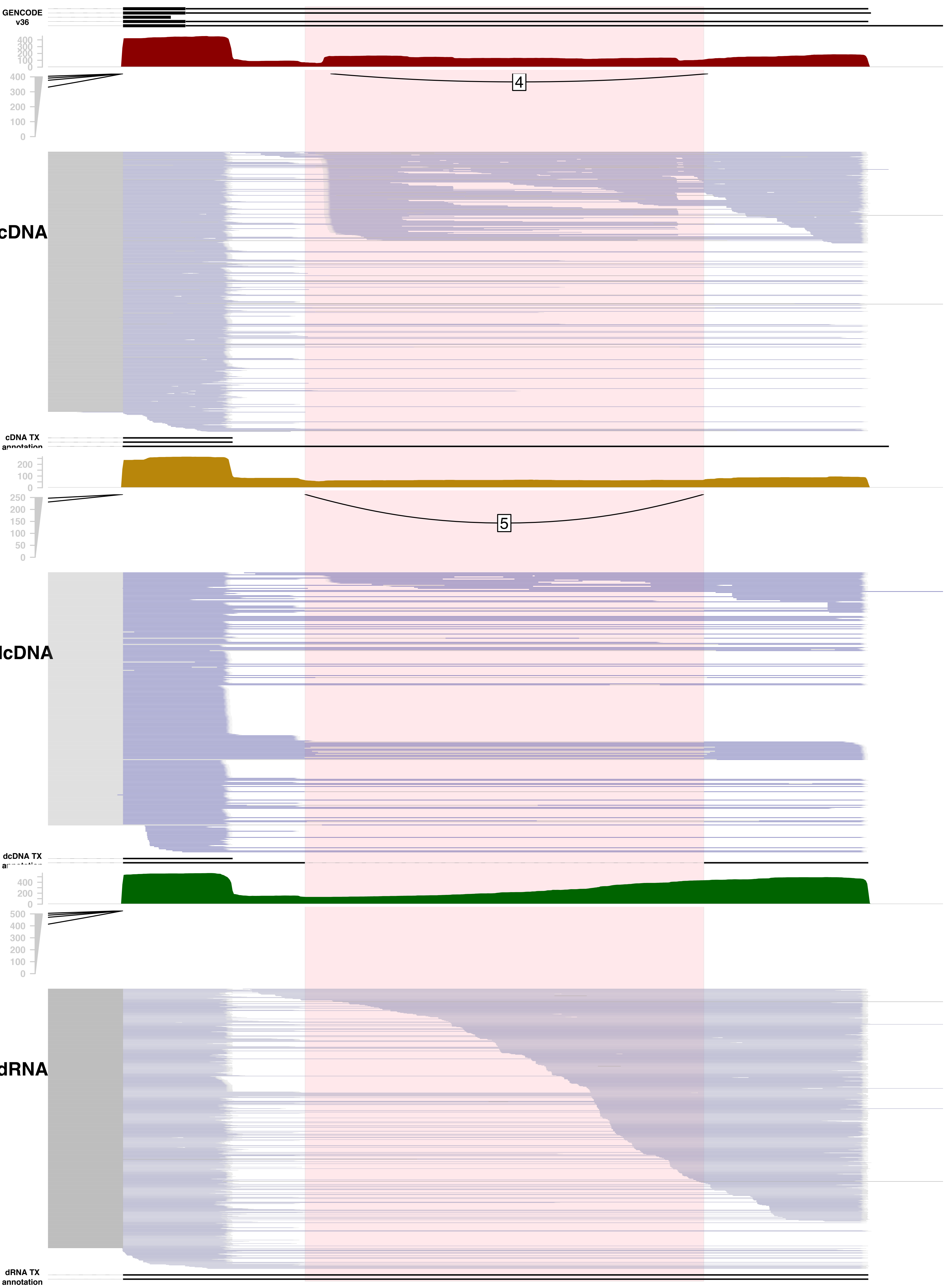

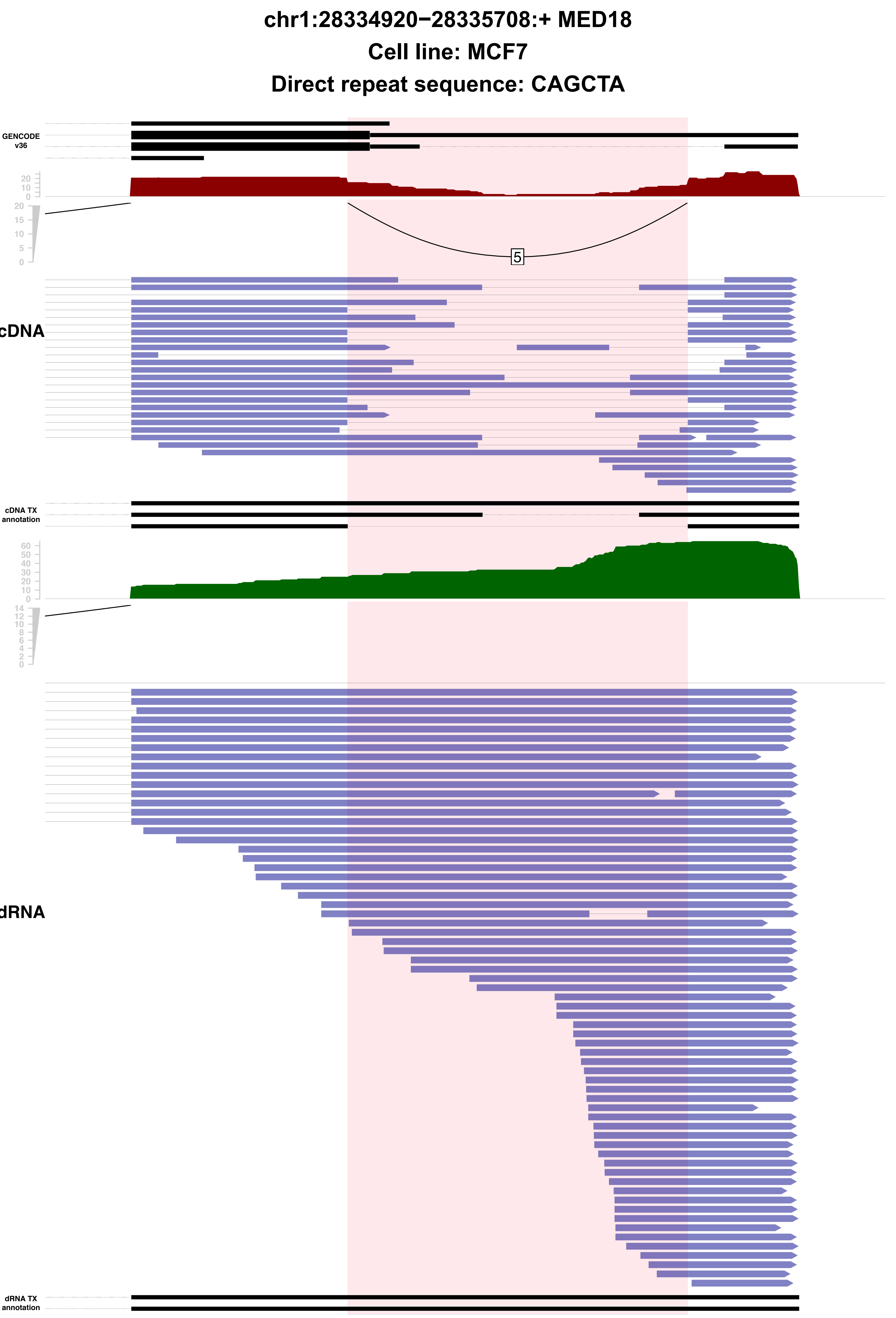
chr1:28334920−28335708:+ MED18
Cell line: MCF7
Direct repeat sequence: CAGCTA
GENCODE v36
cDNA
cDNA TX annotation
dRNA
dRNA TX annotation
5

chr17:3663563−3663657:− TAX1BP3,P2RX5−TAX1BP3
Cell line: MCF7
Direct repeat sequence: GACCG

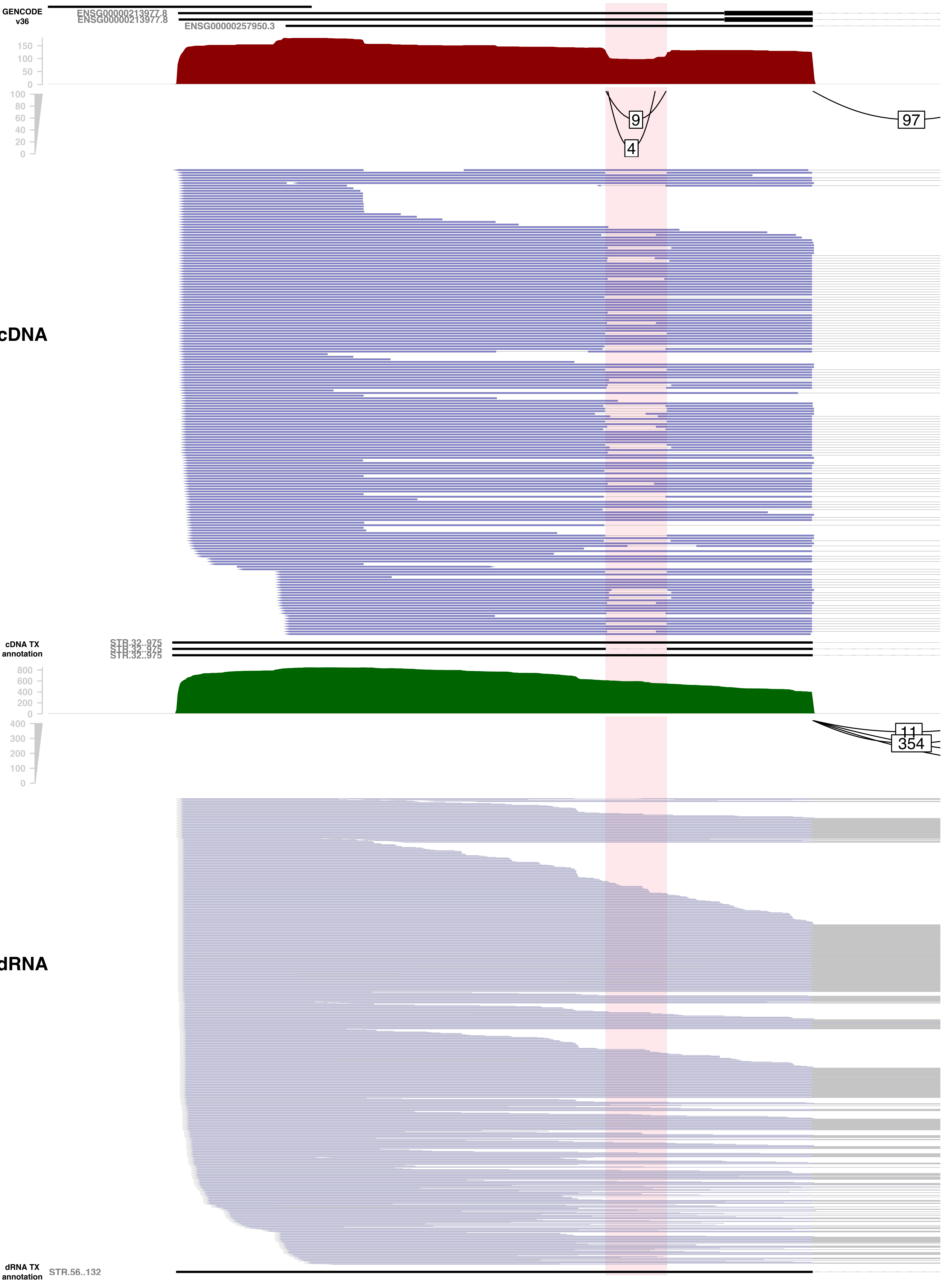

chr19:35746759−35746910:+ PSENEN,AC002398.9

Cell line: MCF7

Direct repeat sequence: CCTG

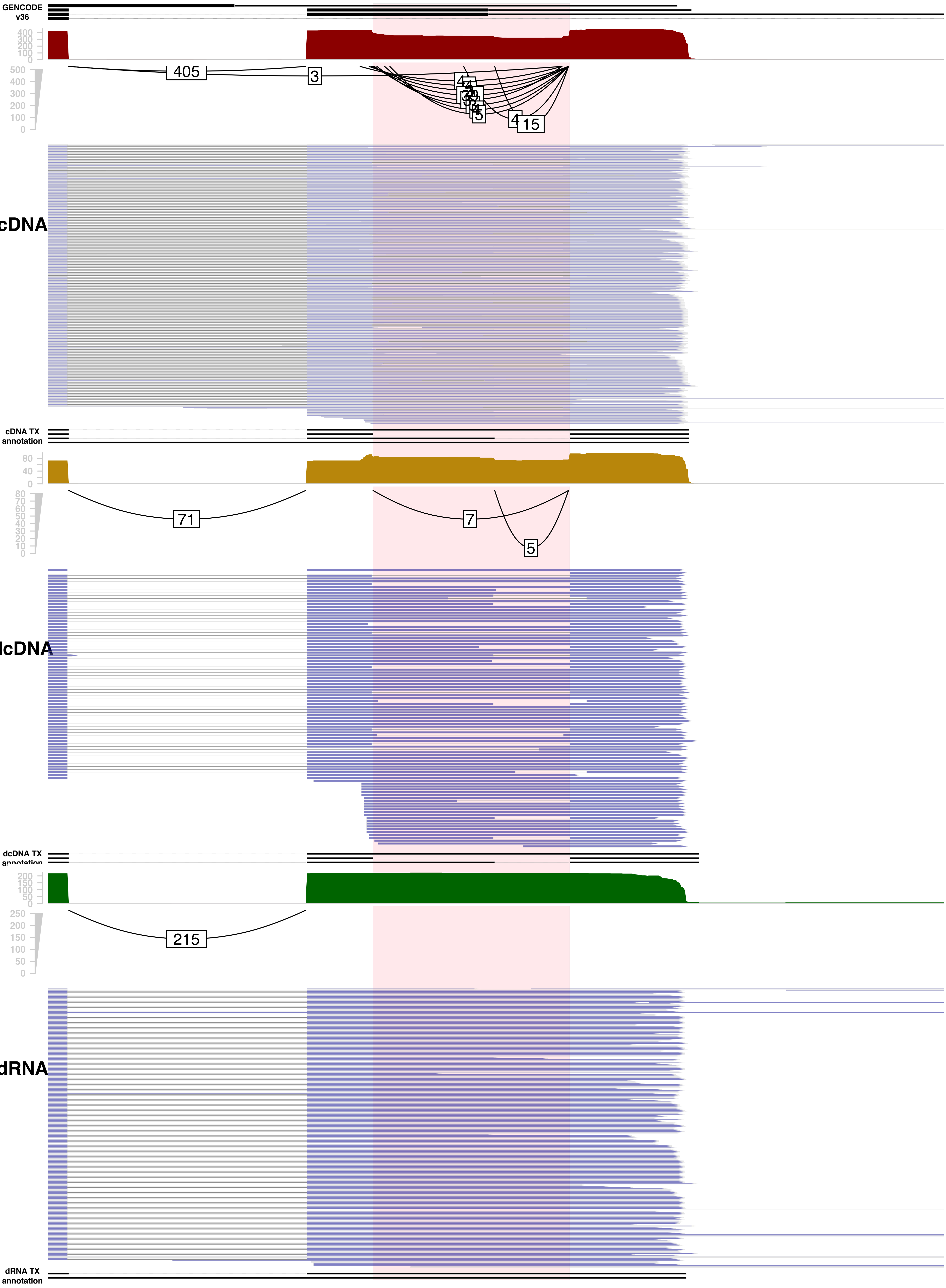

chr19:35746853−35746910:+ PSENEN,AC002398.9
Cell line: MCF7

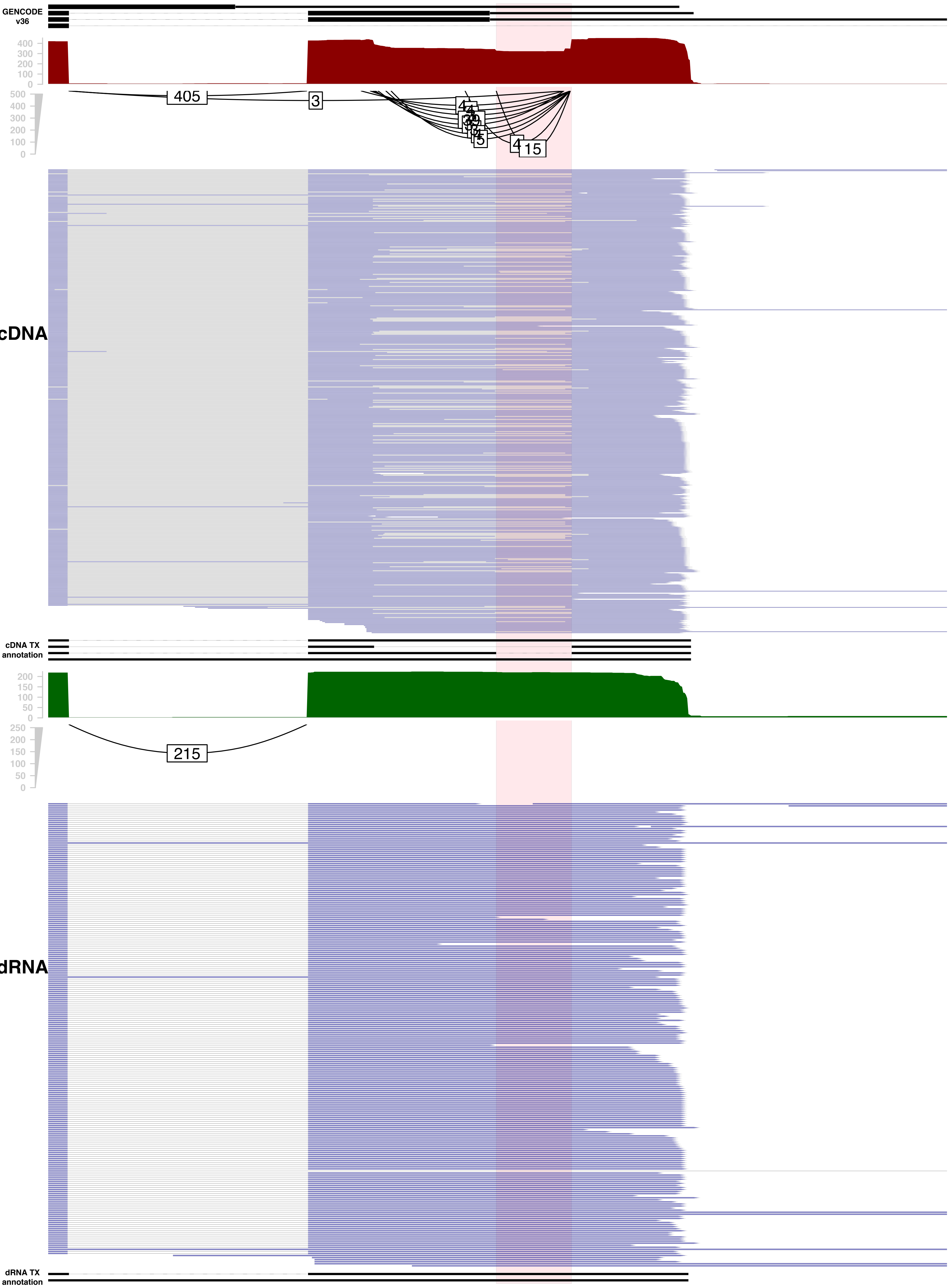

chr20:58891551−58891723:+ GNAS

Cell line: MCF7

Direct repeat sequence: CCGCC

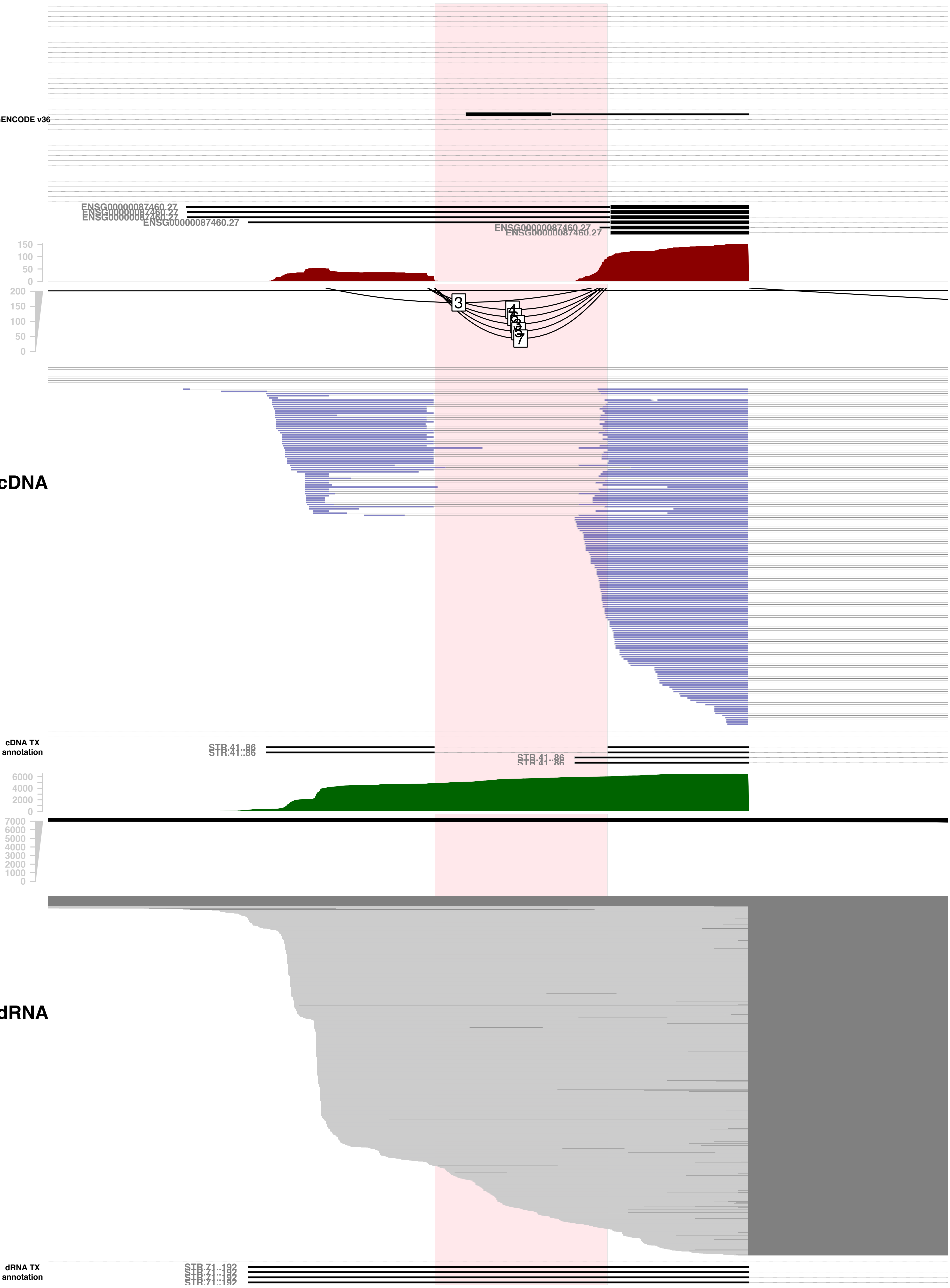

chr22:37807089−37807354:+ H1F0
Cell line: MCF7

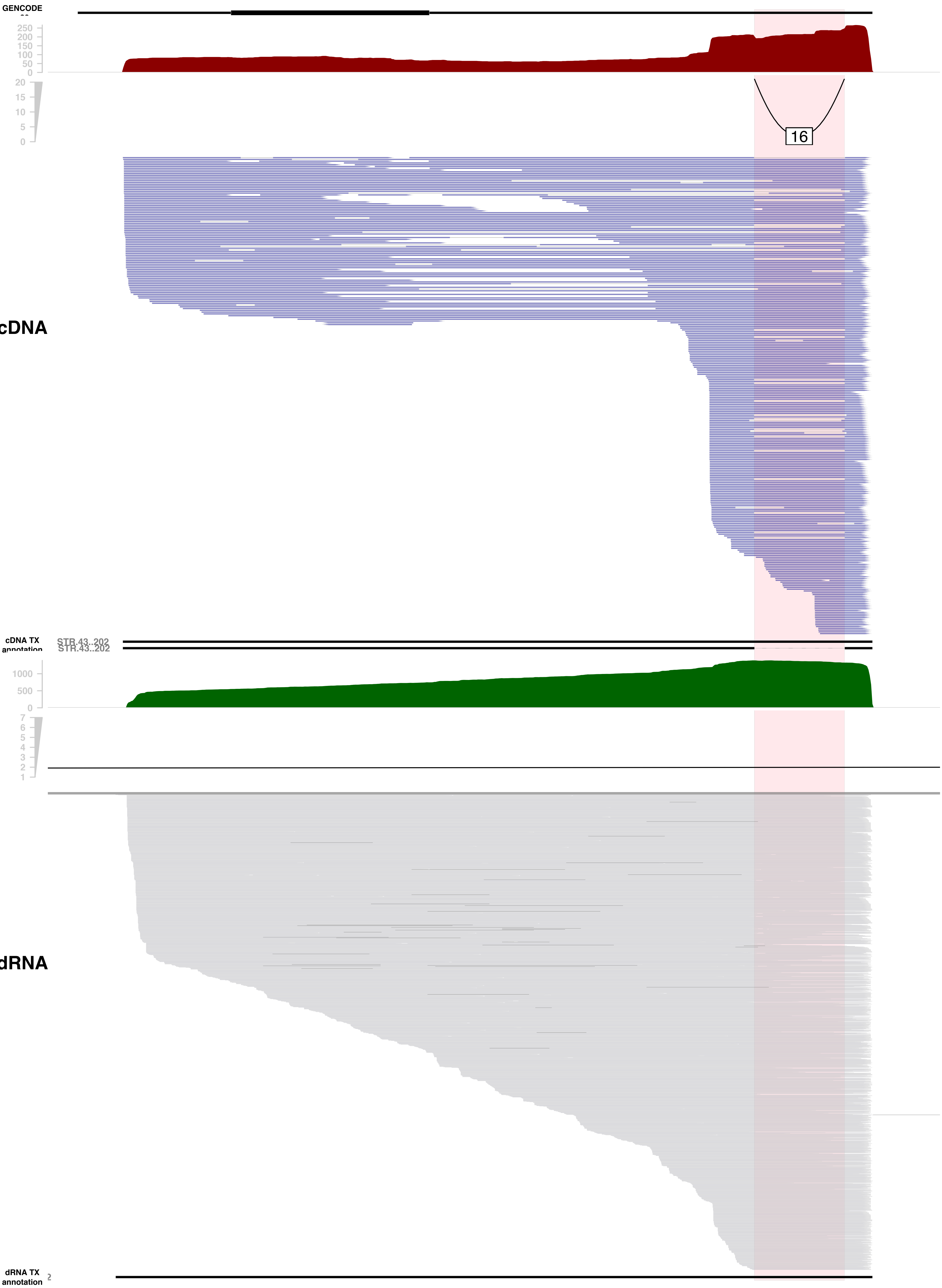

chr5:163437659−163437758:+ CCNG1
Cell line: MCF7

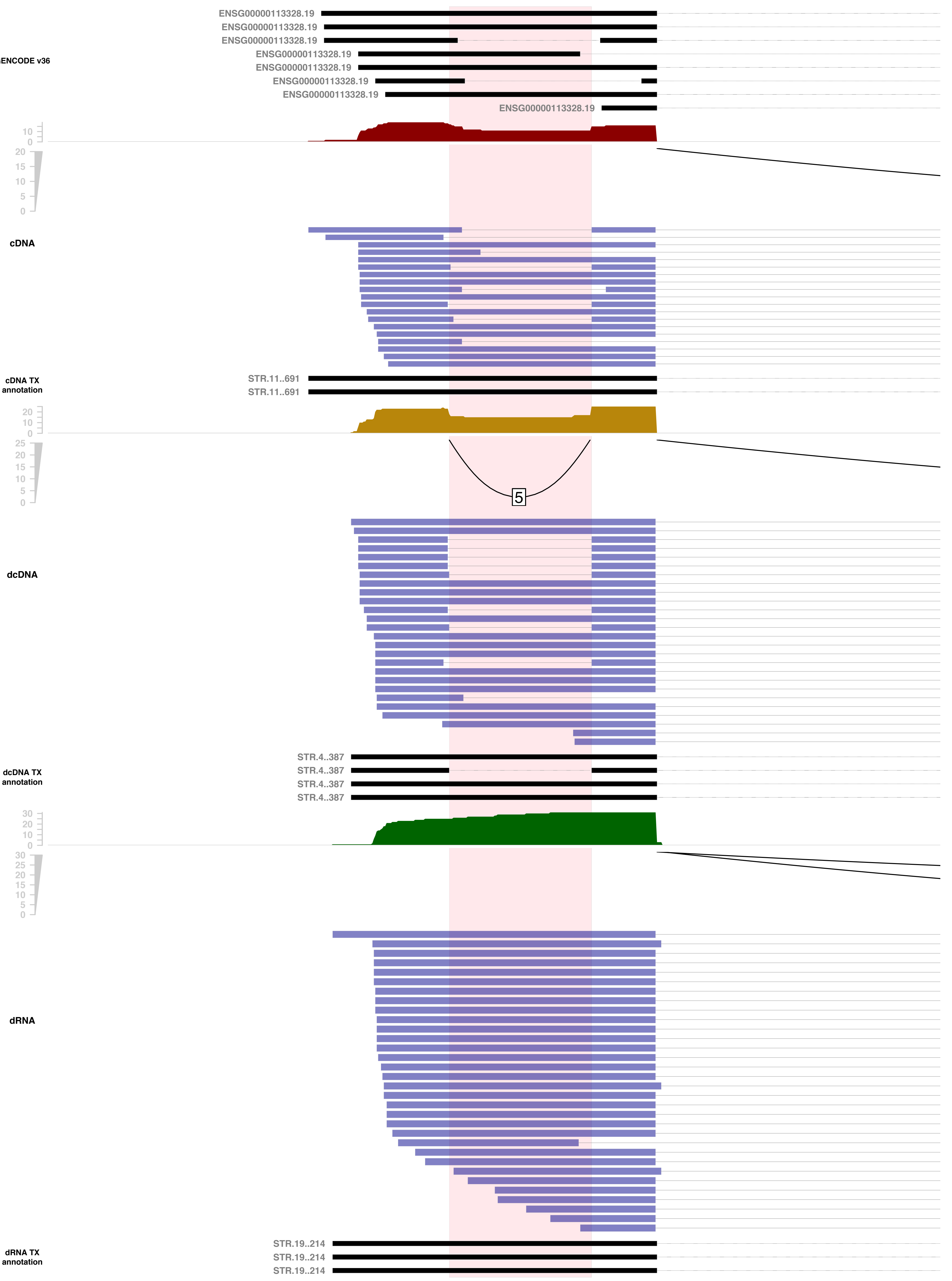

chr1:10457345−10459914:− DFFA

Cell line: NA12878

Direct repeat sequence: AACATAGCAAGACCC

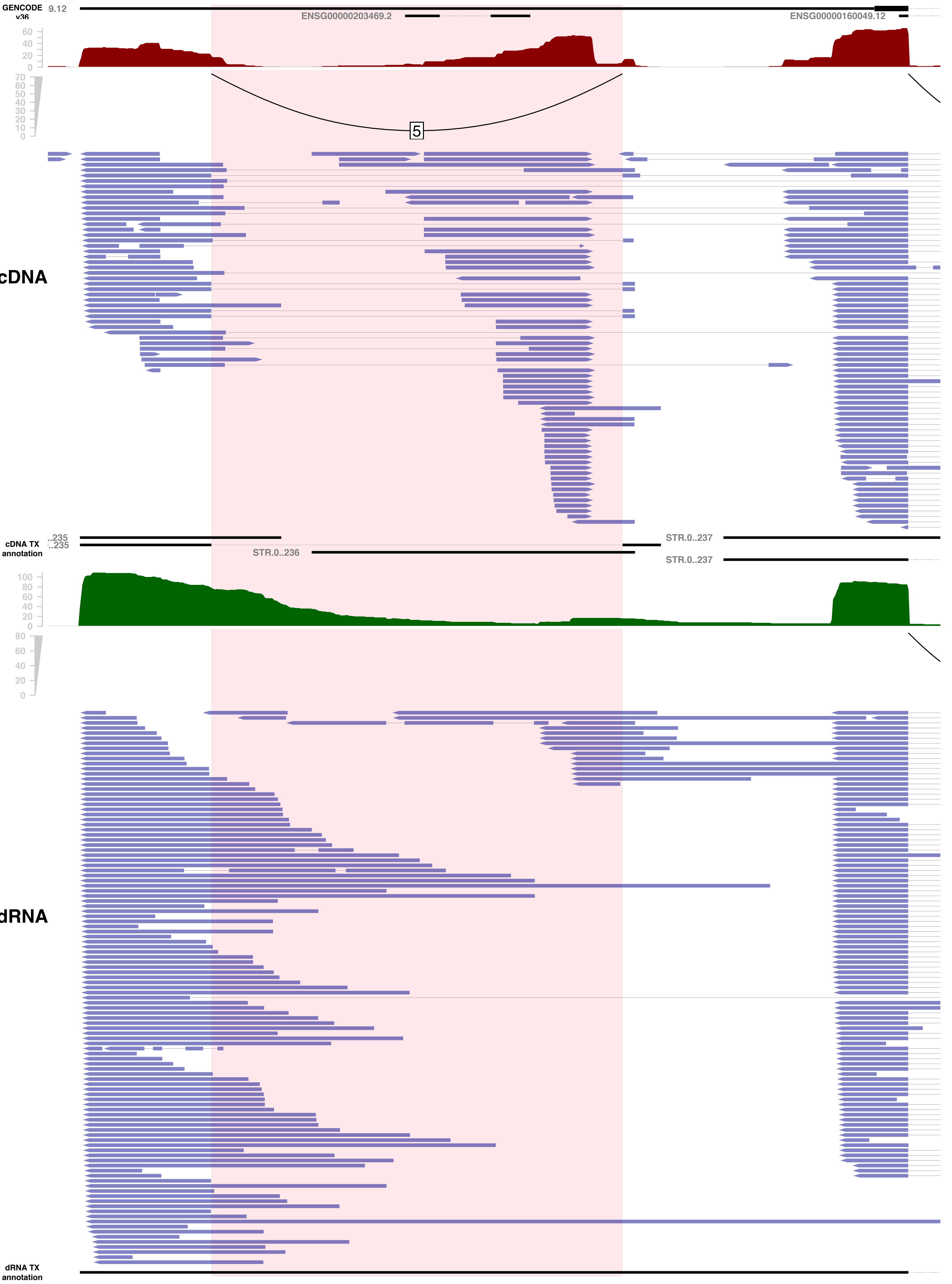

chr19:45703262−45703924:+ QPCTL
Cell line: NA12878
Direct repeat sequence: CAGCC

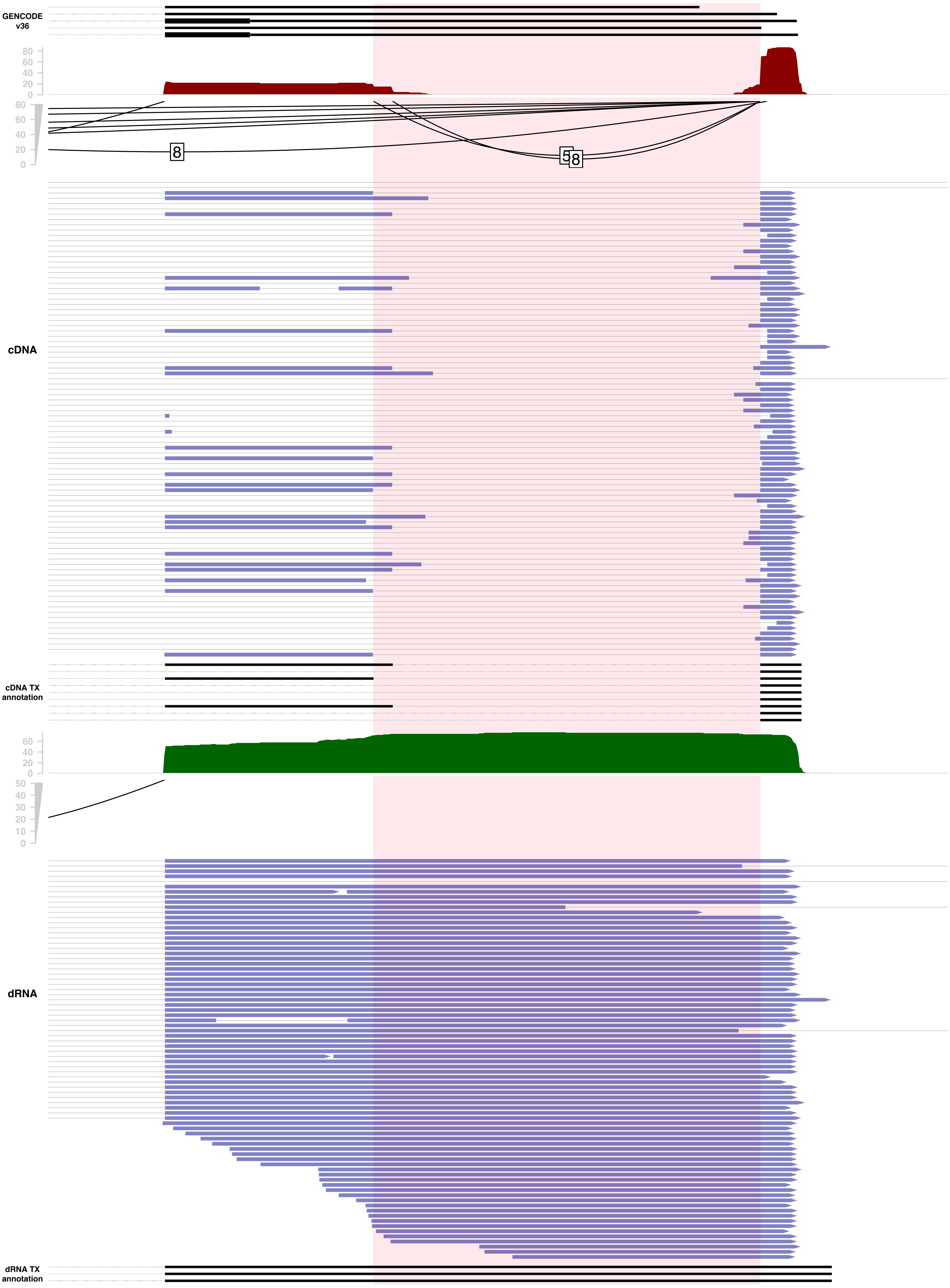

chr20:18487692−18488637:− RBBP9
Cell line: NA12878
Direct repeat sequence: CATG

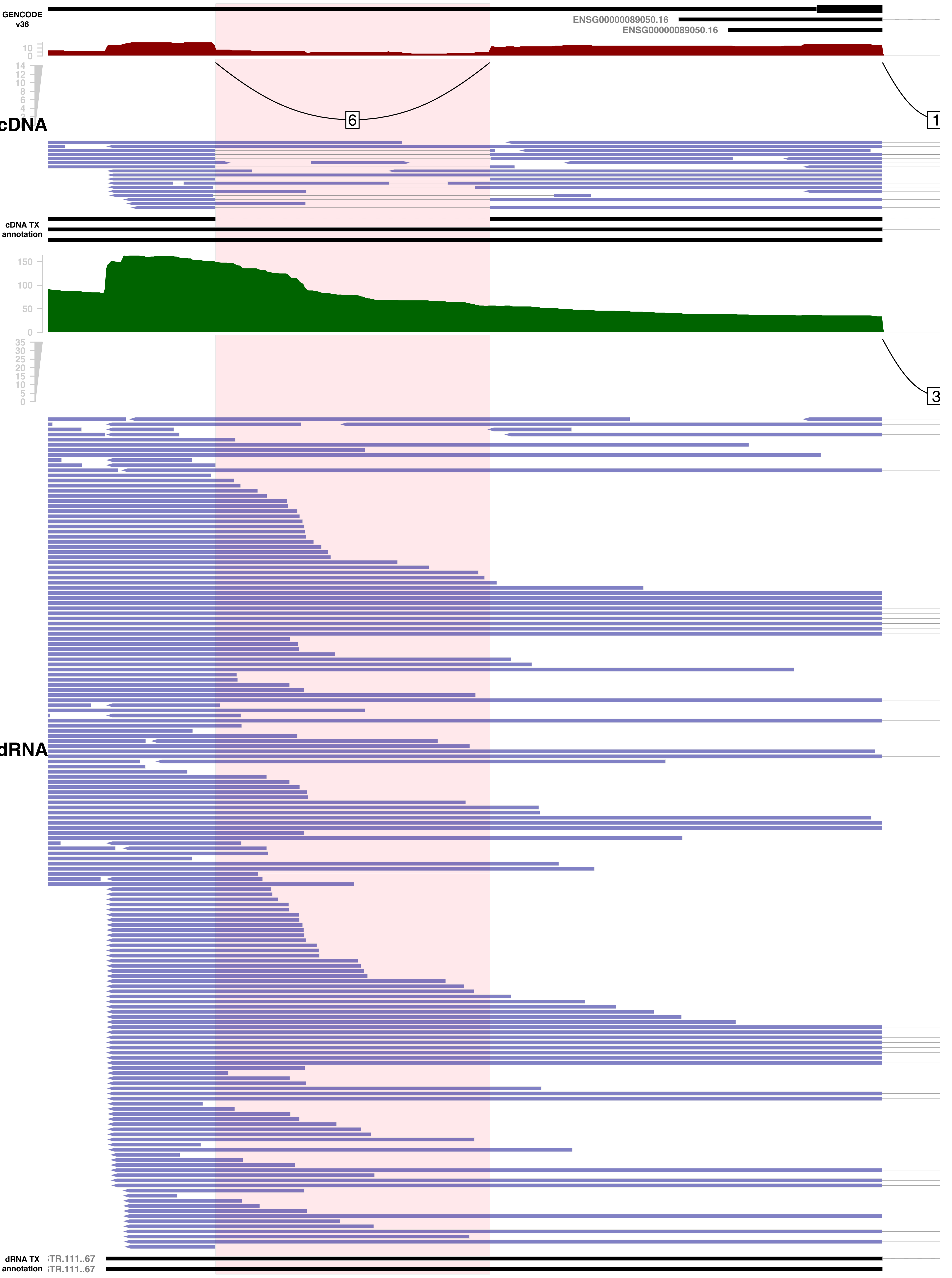

chr2:37202365−37204573:+ CEBPZOS
Cell line: NA12878
Direct repeat sequence: CAGCCA

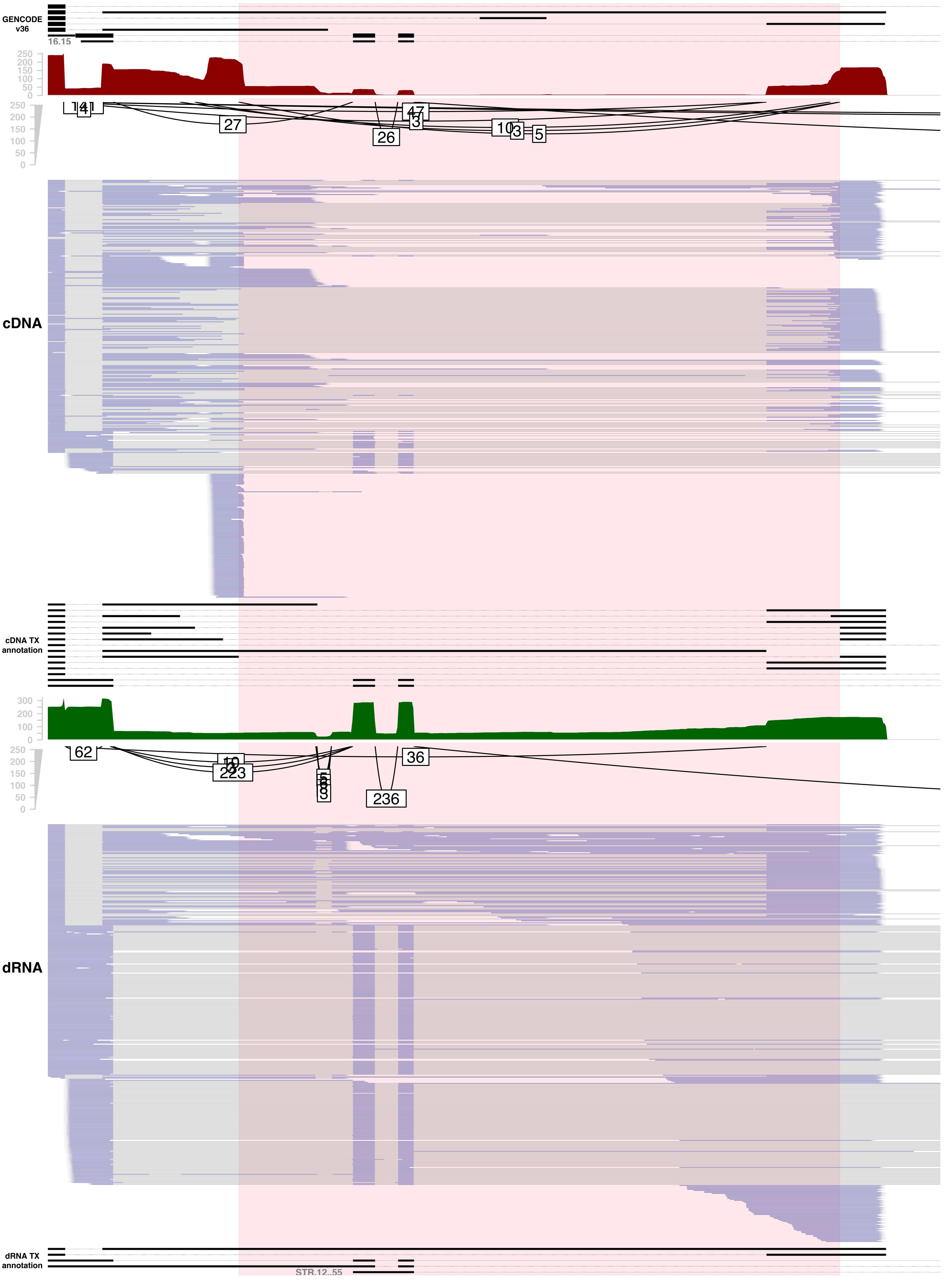

chr2:37202149−37204539:+ CEBPZOS
Cell line: NA12878

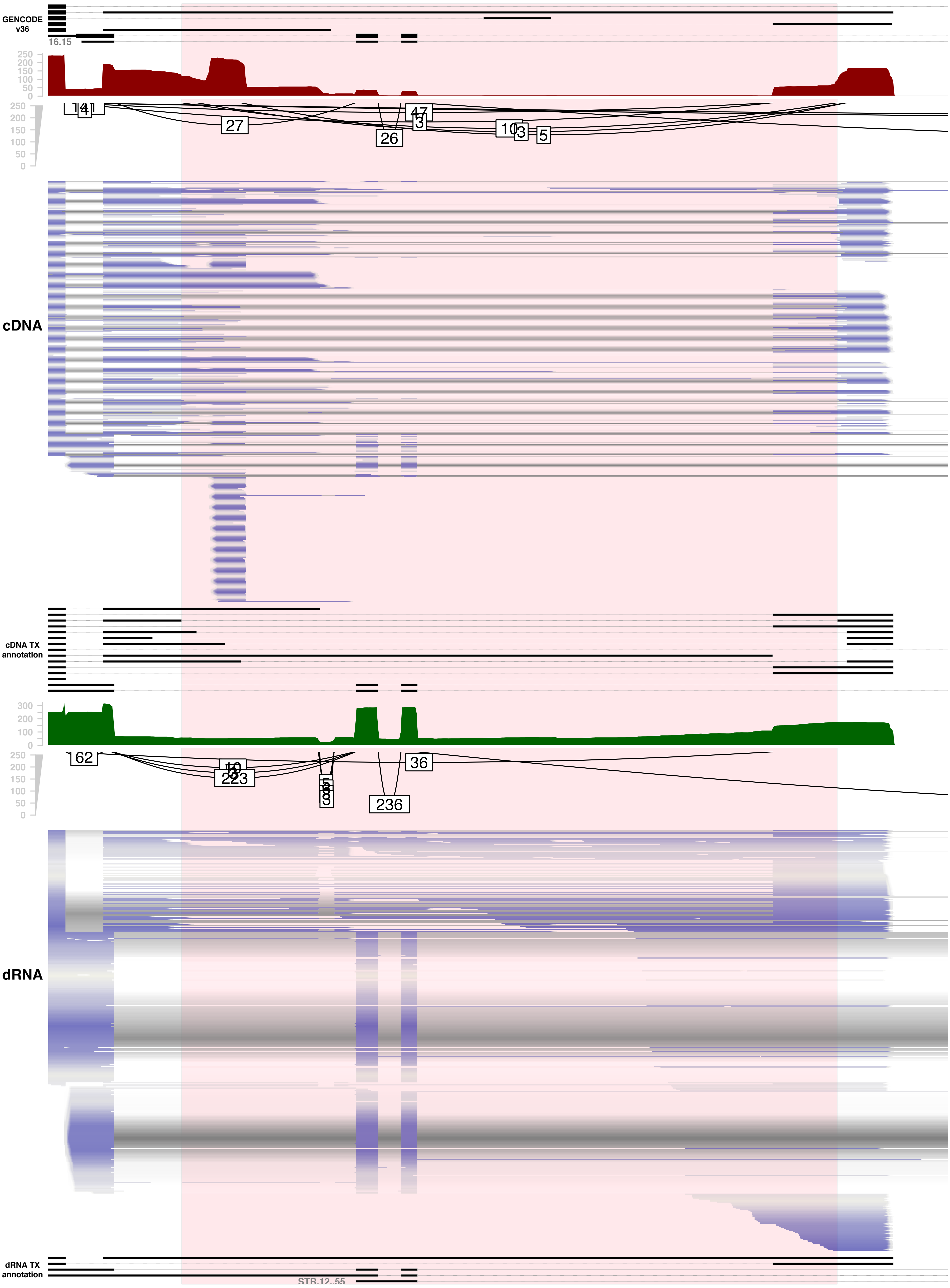

chrX:119583452−119584366:+ UBE2A

Cell line: NA12878

Direct repeat sequence: ATGTA

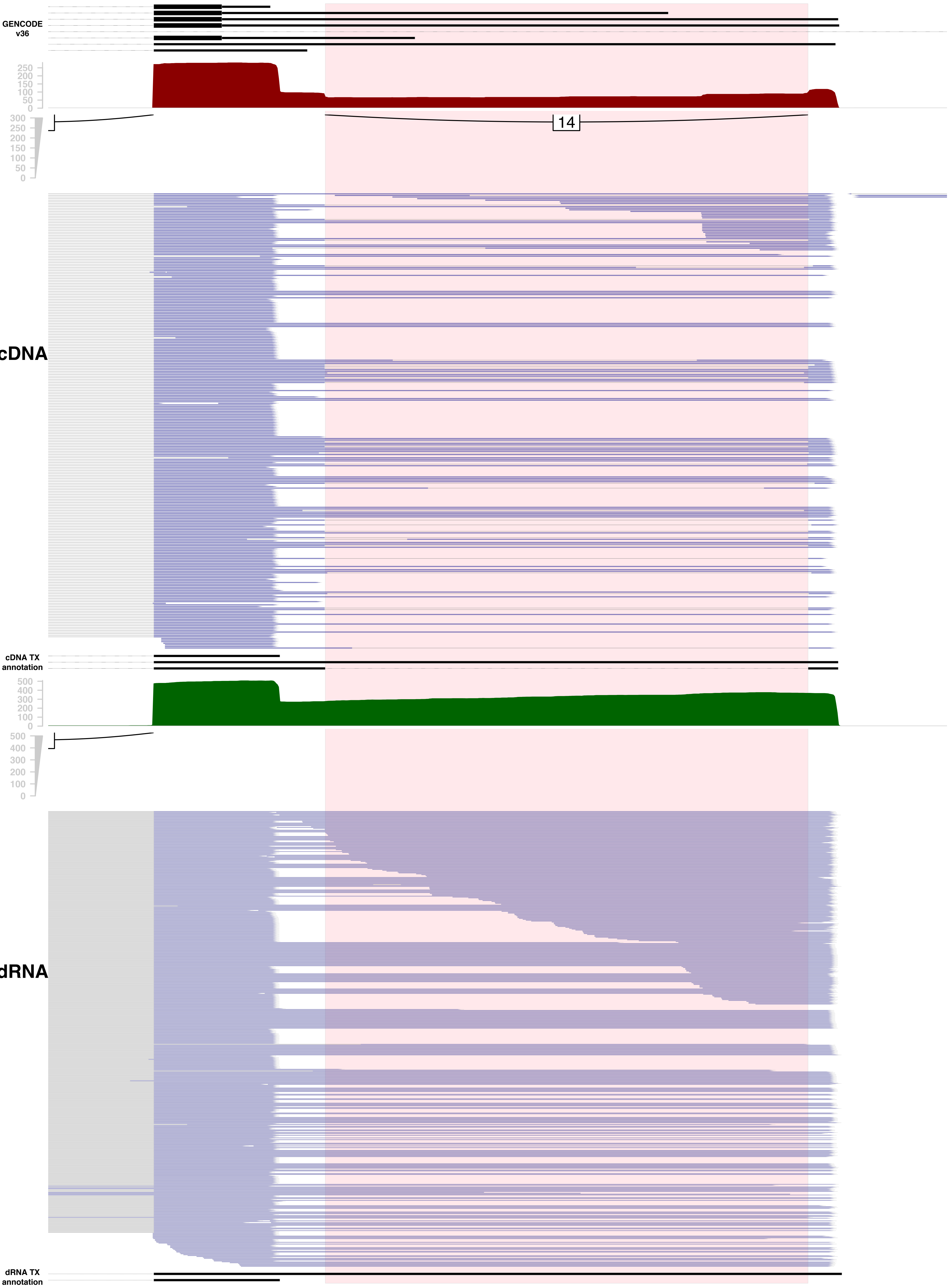

chr2:113204982−113205613:+ PSD4
Cell line: NA12878

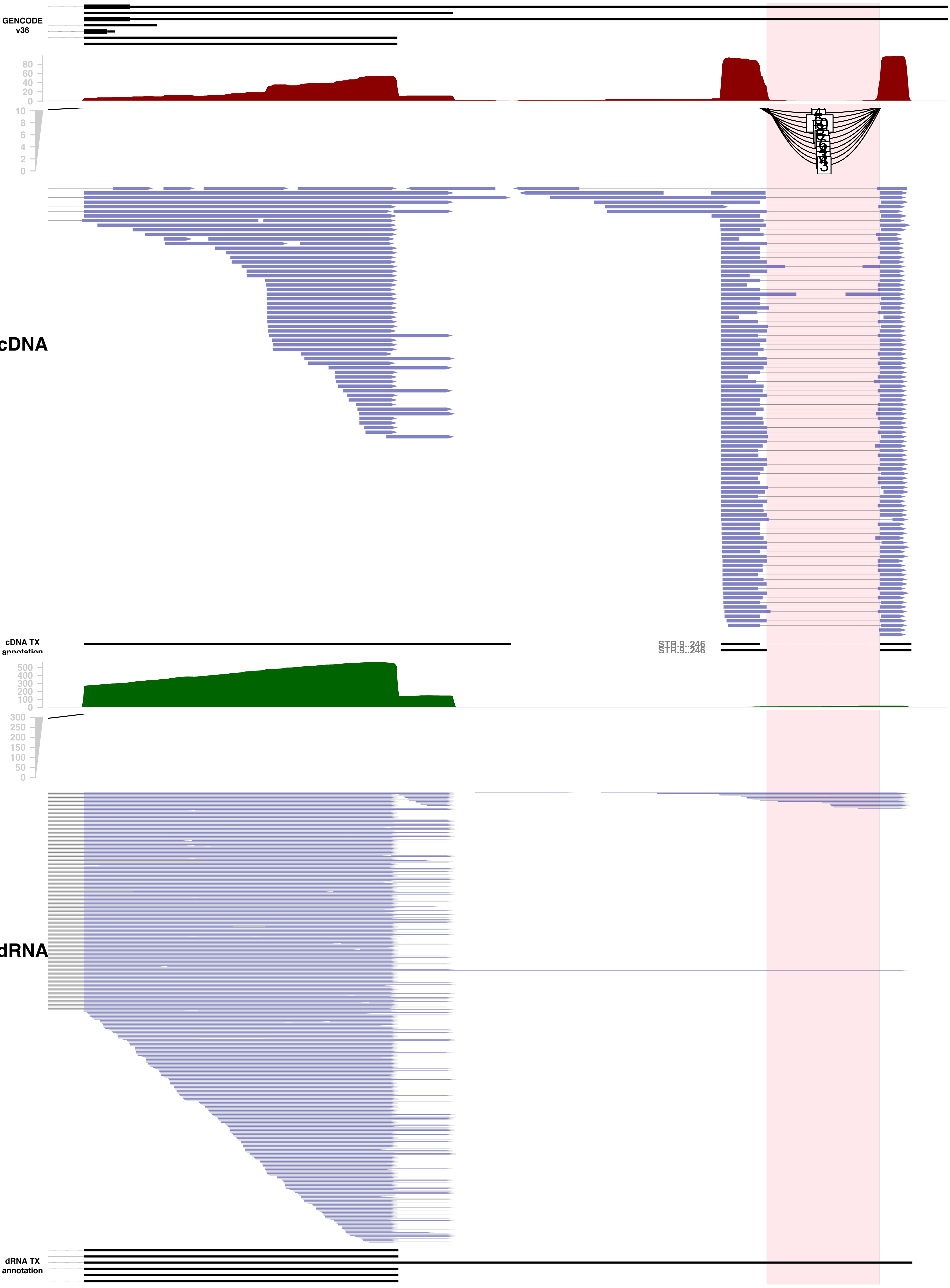

chr4:56459858−56460922:+ PAICS
Cell line: NA12878

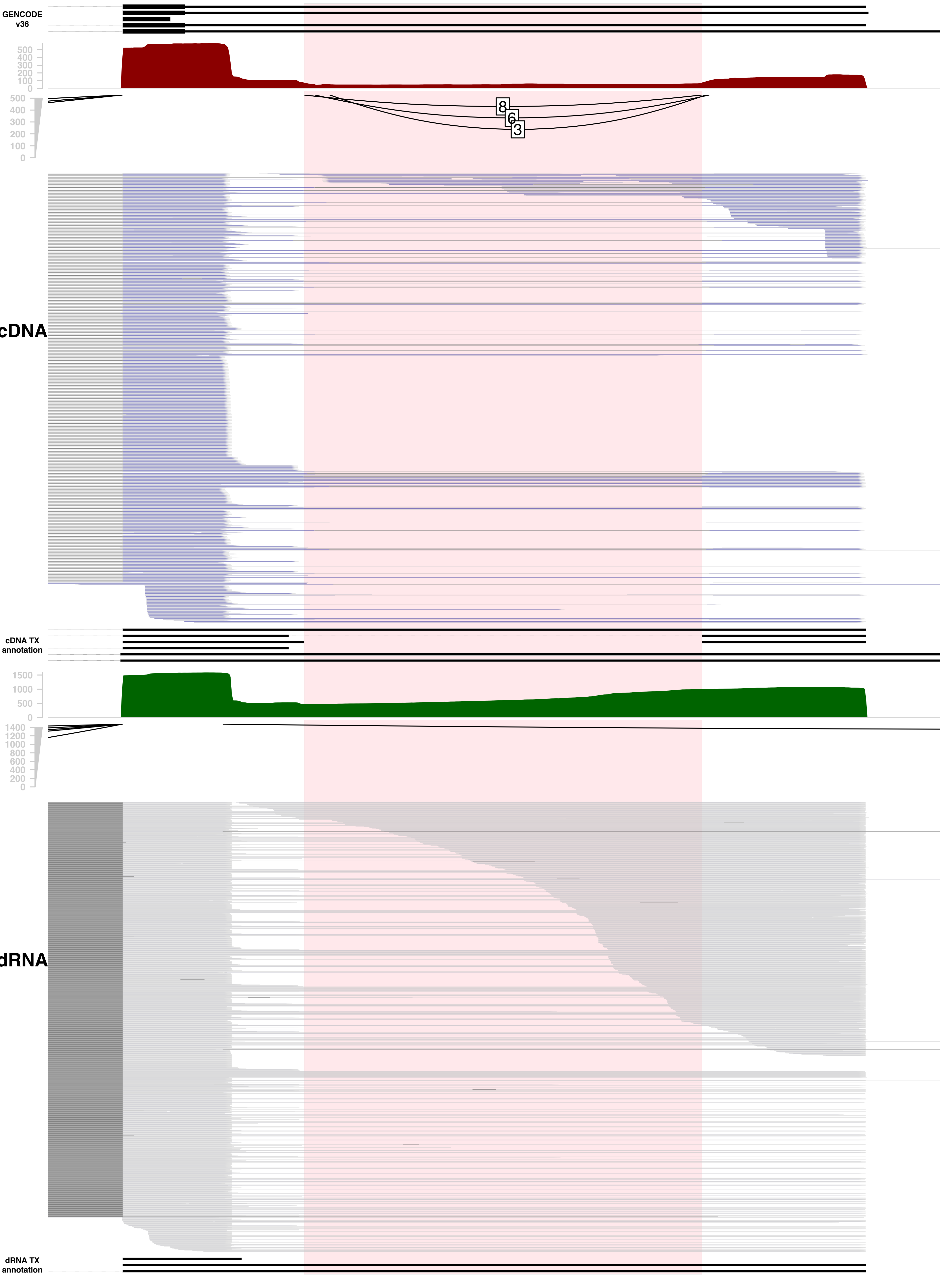

chr4:2938328−2938803:− NOP14
Cell line: NA12878
Direct repeat sequence: CTGG

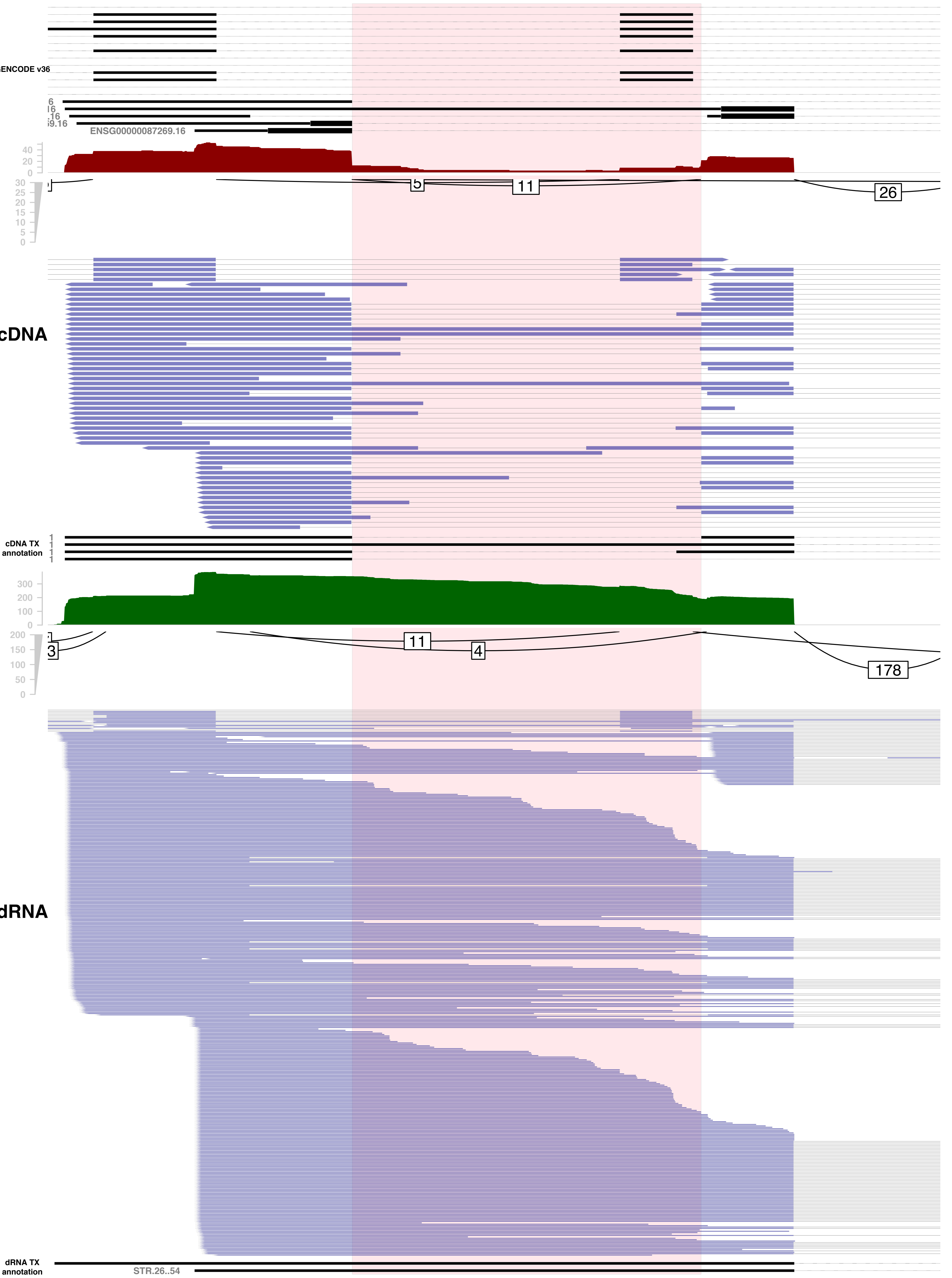

chr5:163437659−163437758:+ CCNG1
Cell line: NA12878

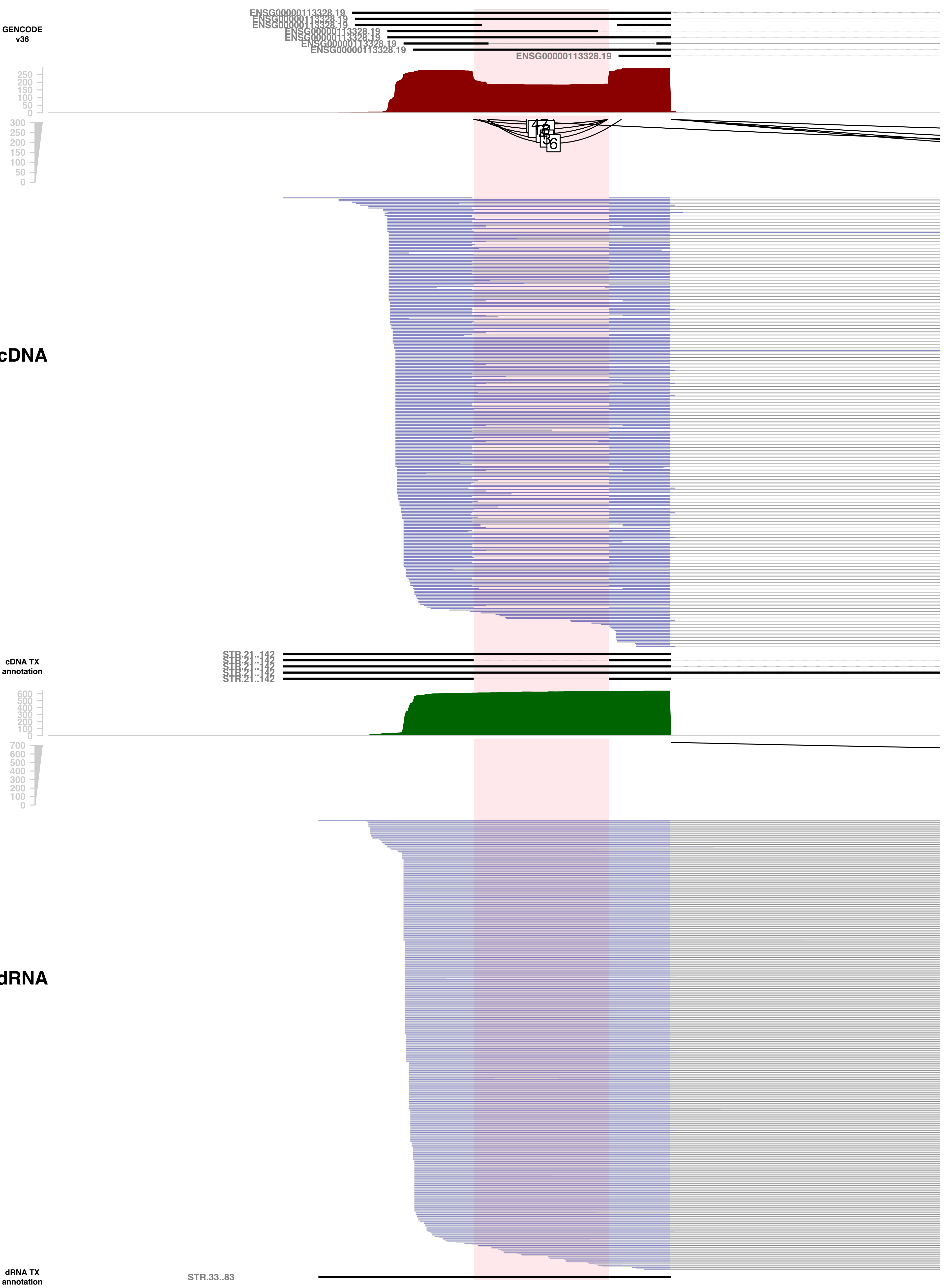

chr5:180835052−180835421:+ LINC00847
Cell line: NA12878
Direct repeat sequence: GCCTTC

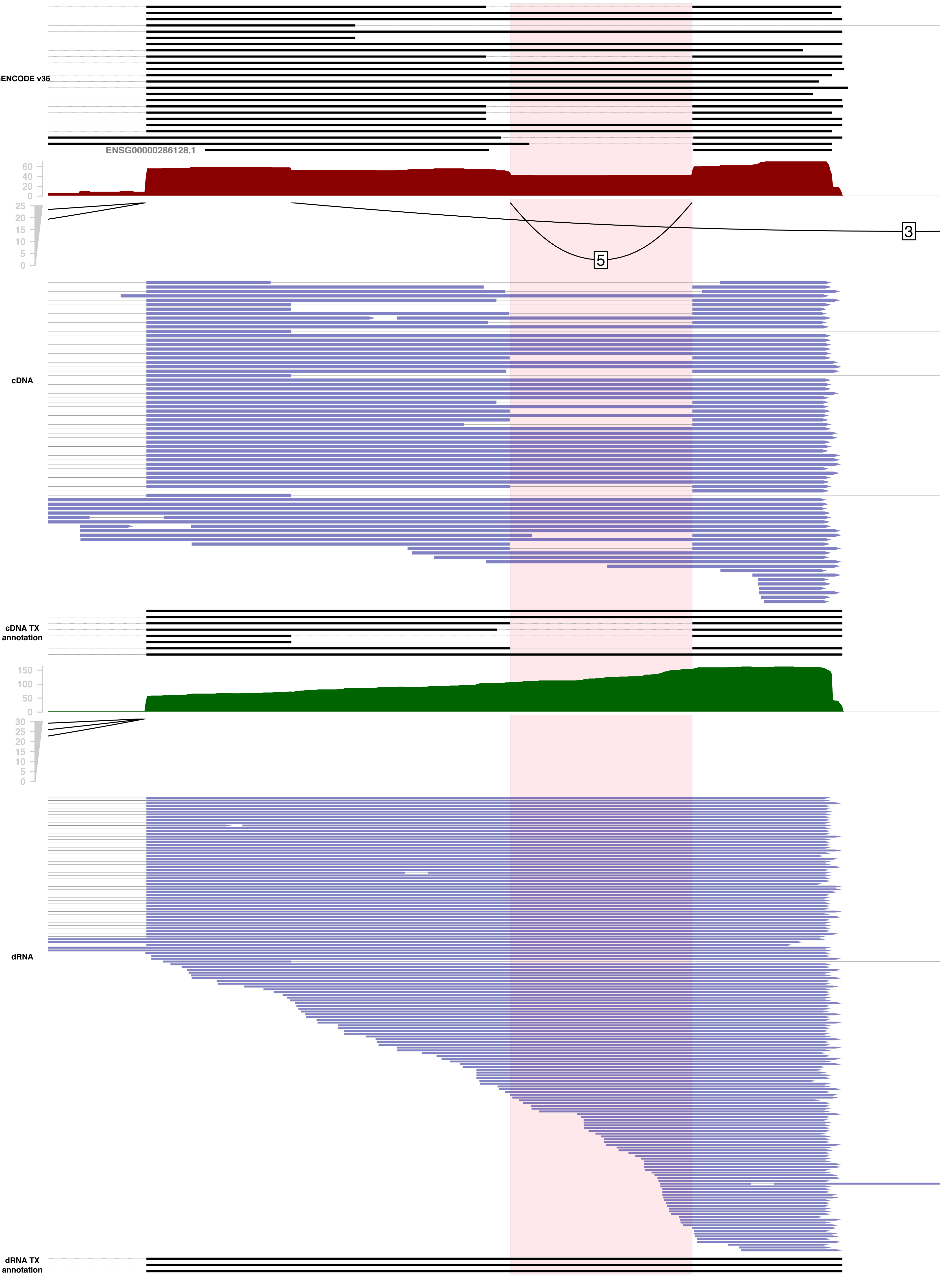

chr6:70588932−70589284:+ SDHAF4

Cell line: NA12878

Direct repeat sequence: GCCT

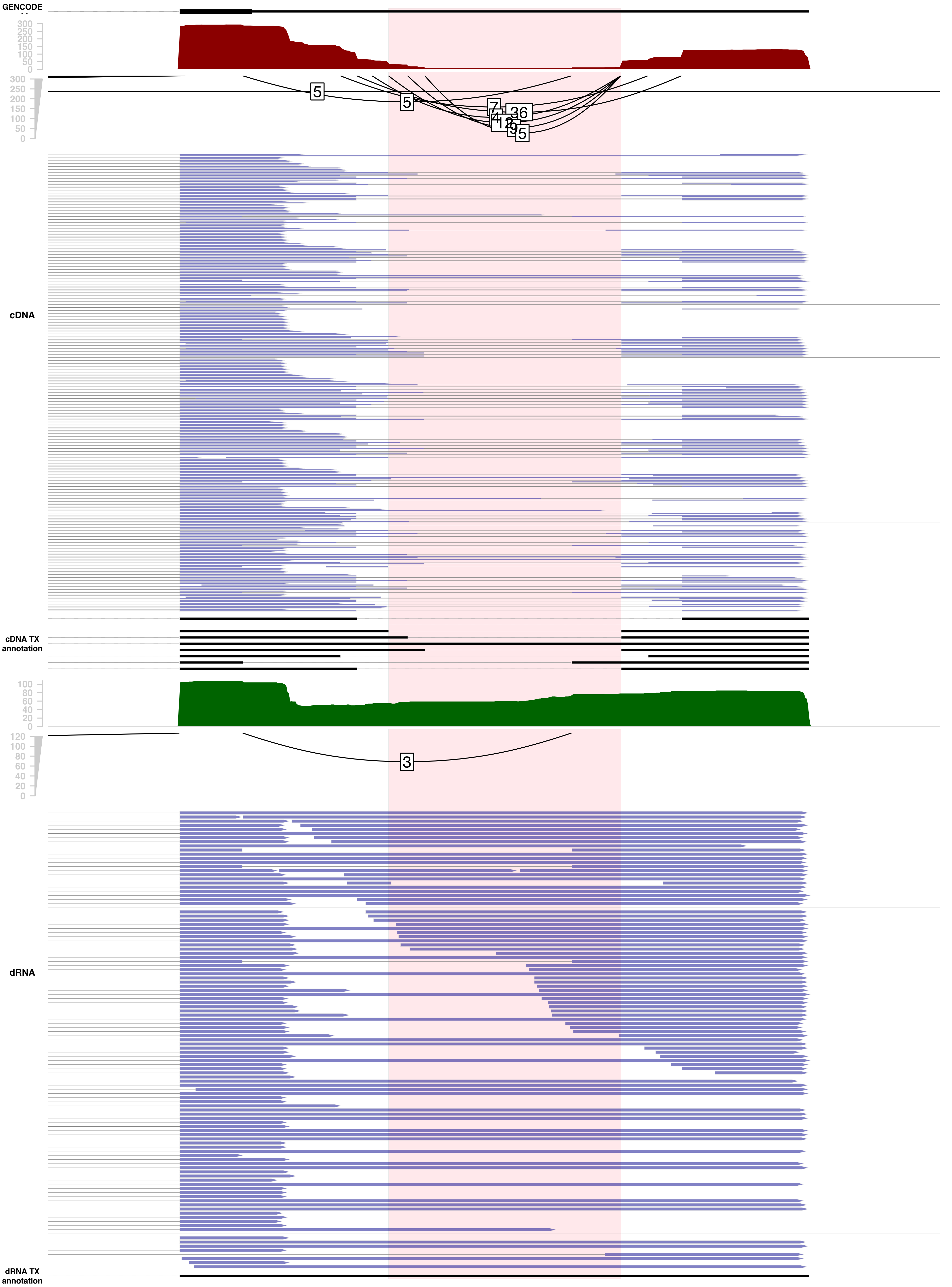

chr6:70588987−70589284:+ SDHAF4
Cell line: NA12878

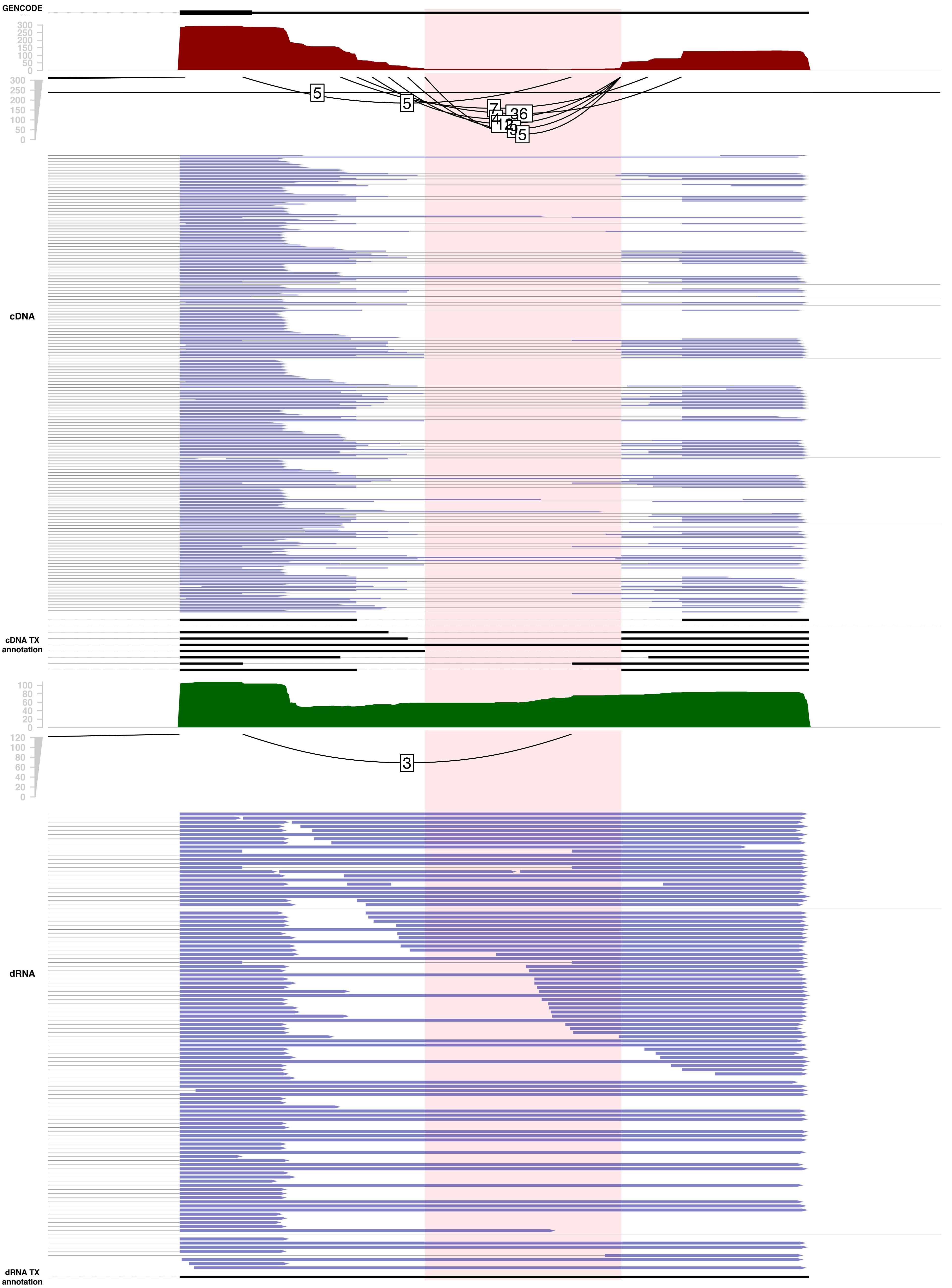

chr6:70588859−70589325:+ SDHAF4

Cell line: NA12878

Direct repeat sequence: TGGCT

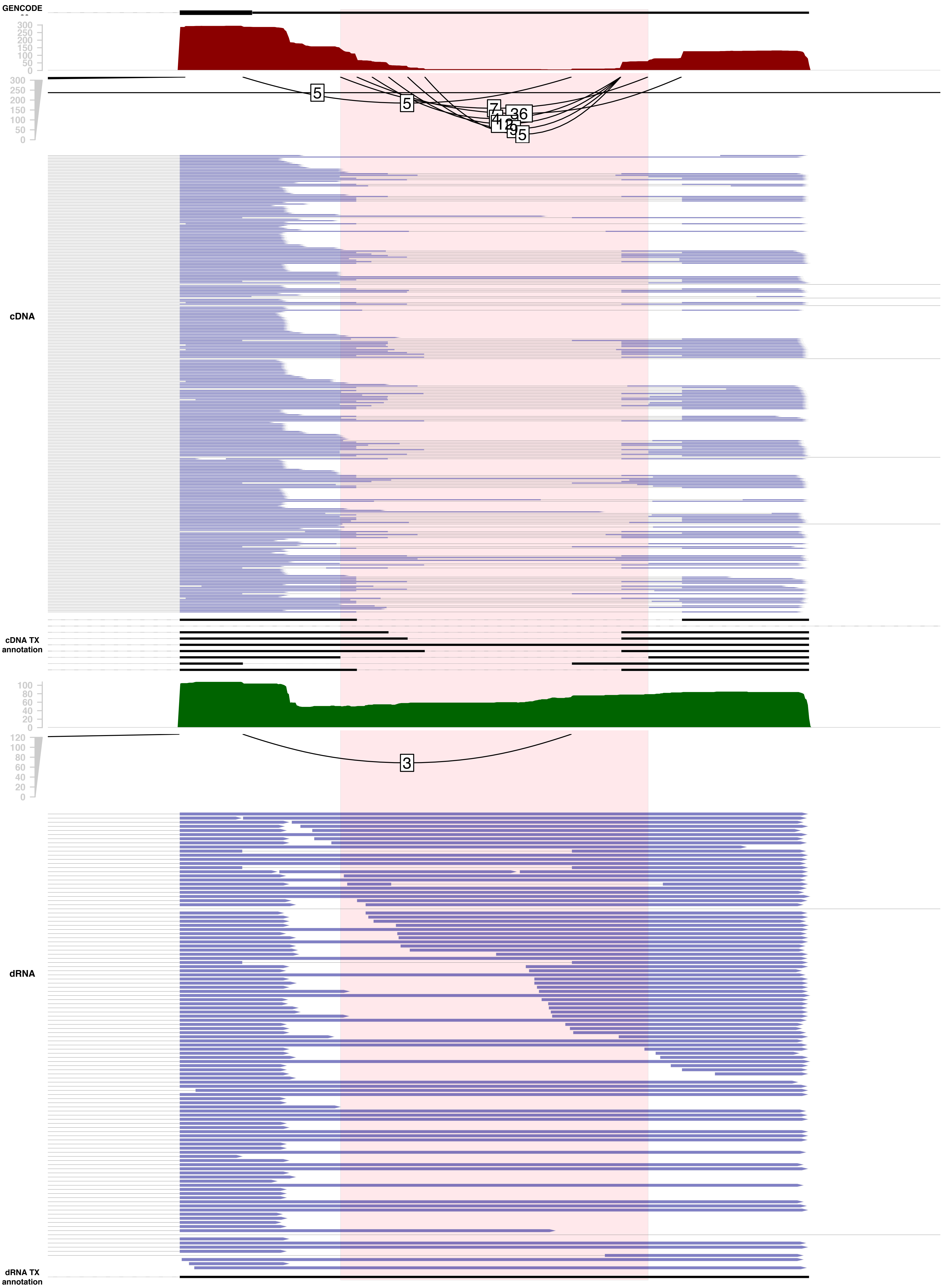

chr6:170584410−170584525:− PDCD2
Cell line: NA12878

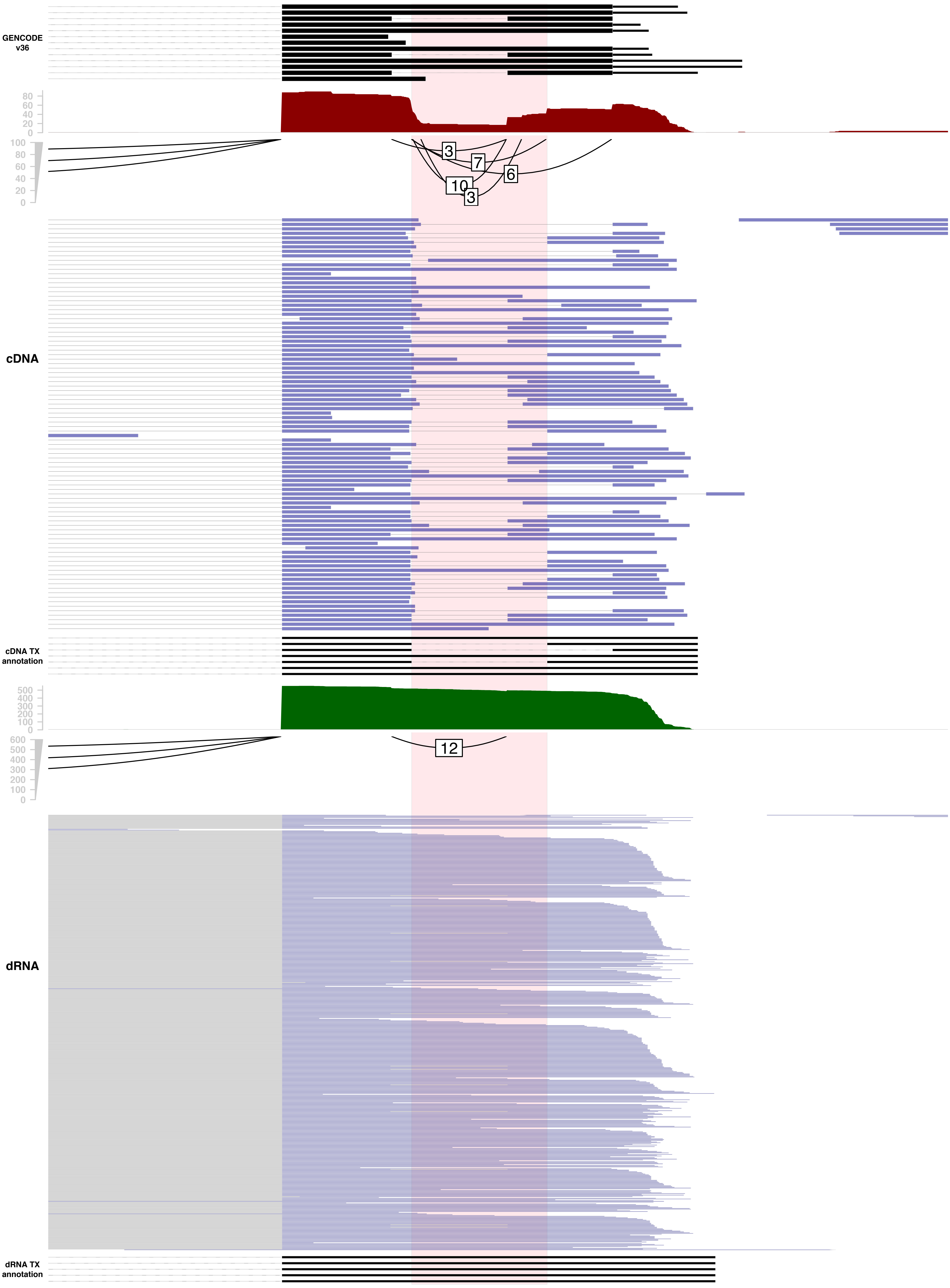

chr6:170584410−170584581:− PDCD2
Cell line: NA12878
Direct repeat sequence: CCCGC

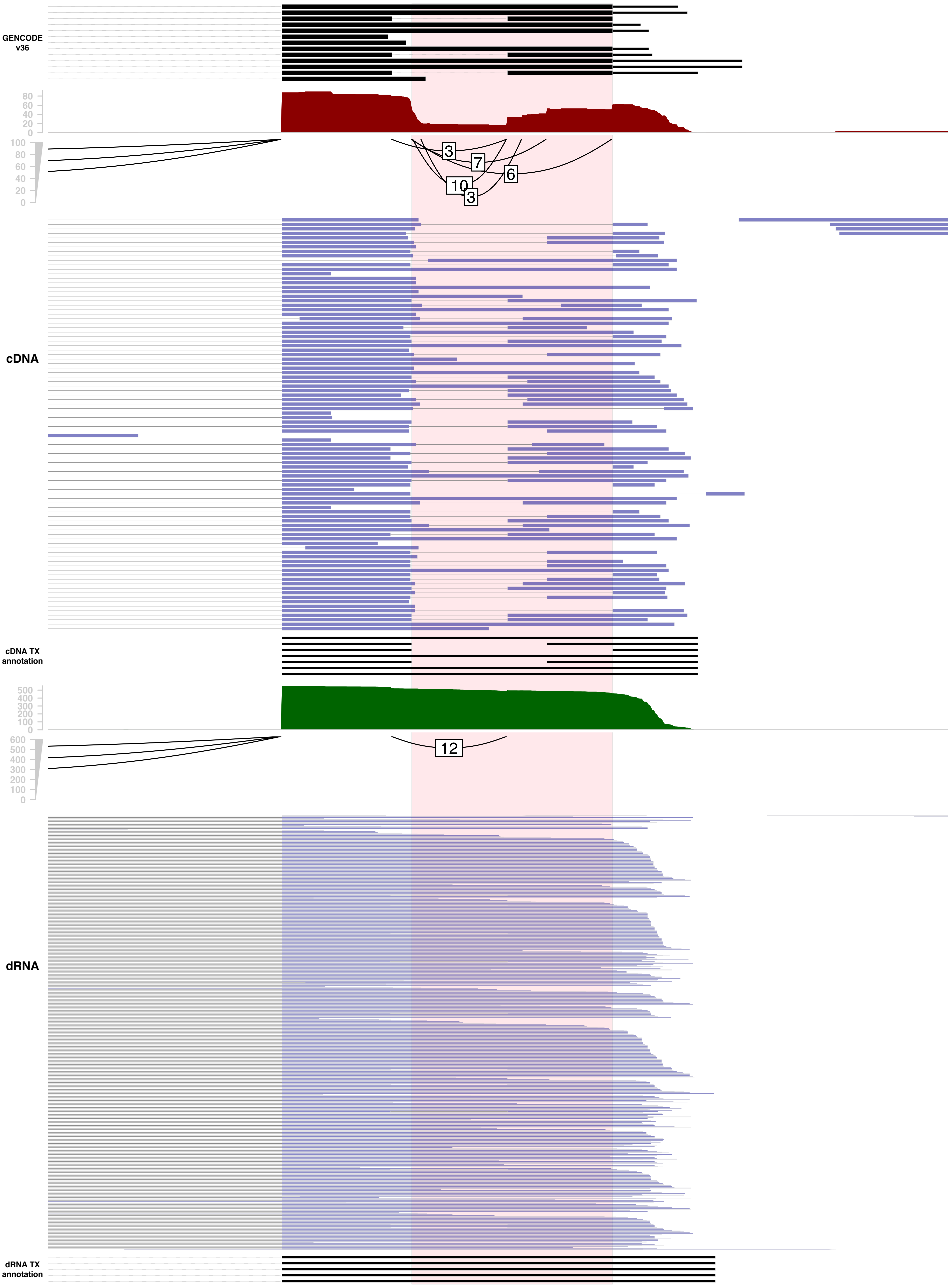

chr14:21460346−21460977:− RAB2B

Cell line: NA12878

Direct repeat sequence: CCTCC

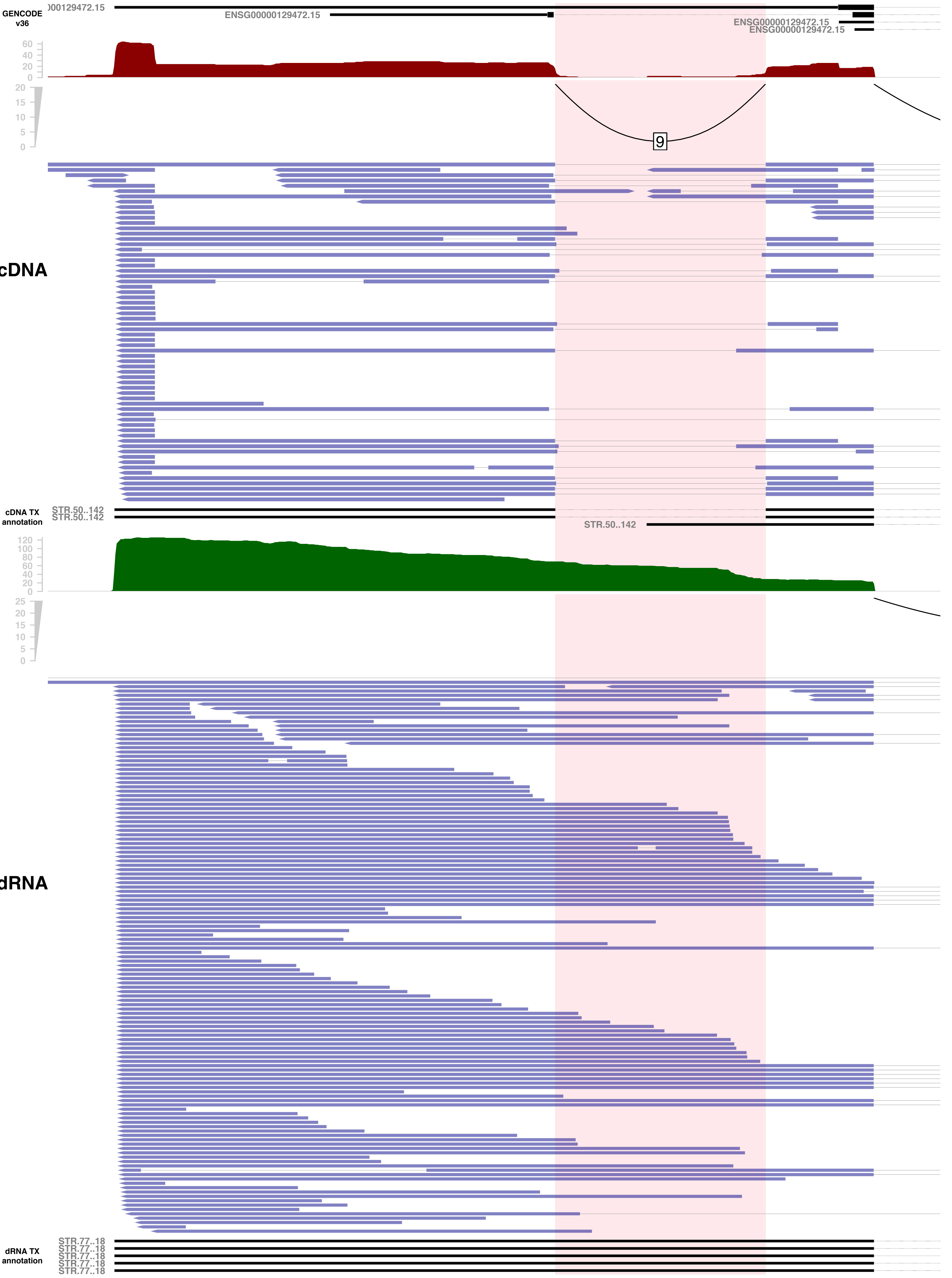

chr15:50706125−50707523:− SPPL2A
Cell line: NA12878
Direct repeat sequence: CCACC

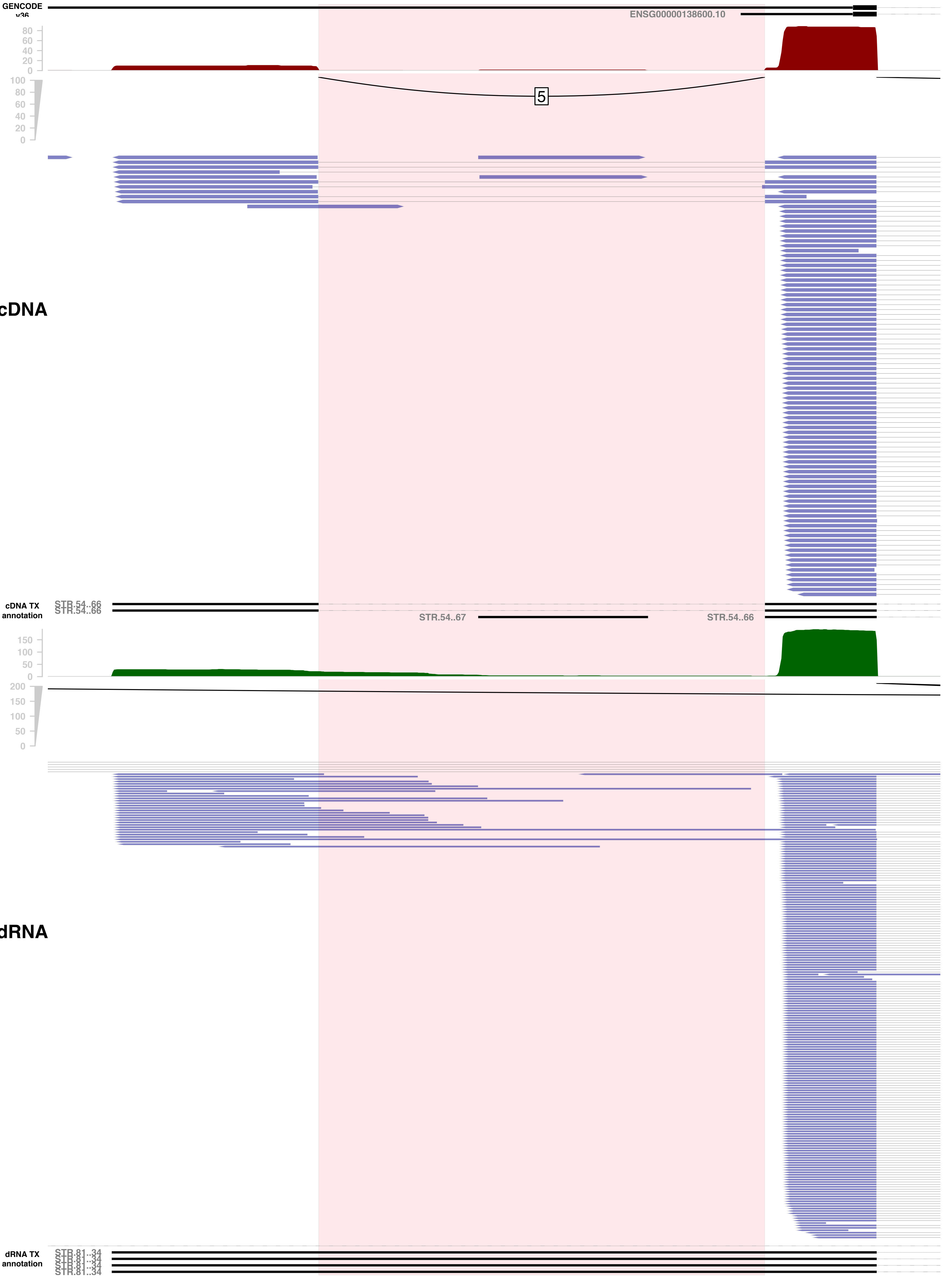

chr16:28932452−28932581:+ CD19
Cell line: NA12878
Direct repeat sequence: CAGCCTGG

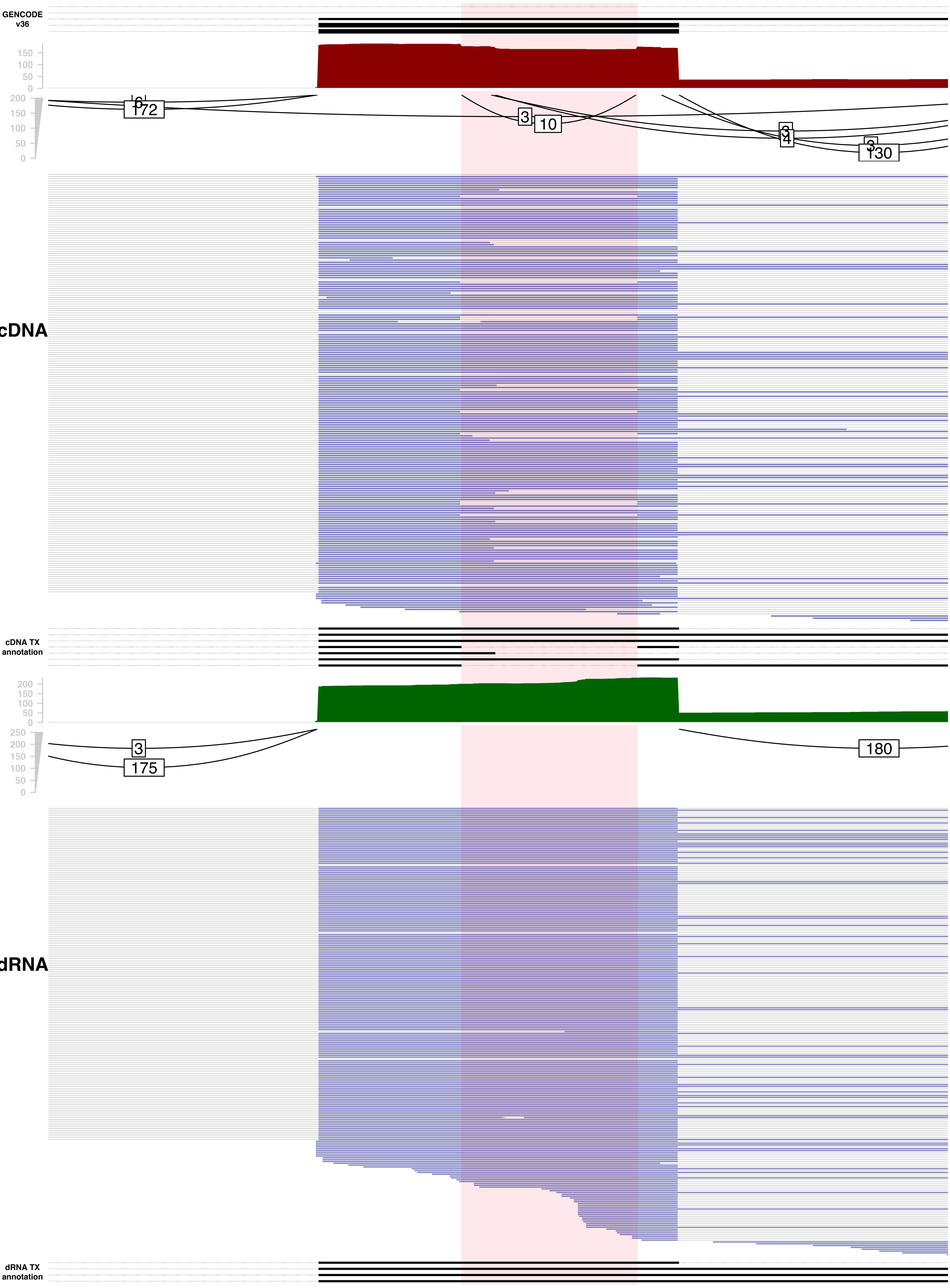

chr17:3663563−3663657:− TAX1BP3,P2RX5−TAX1BP3

Cell line: NA12878

Direct repeat sequence: GACCG

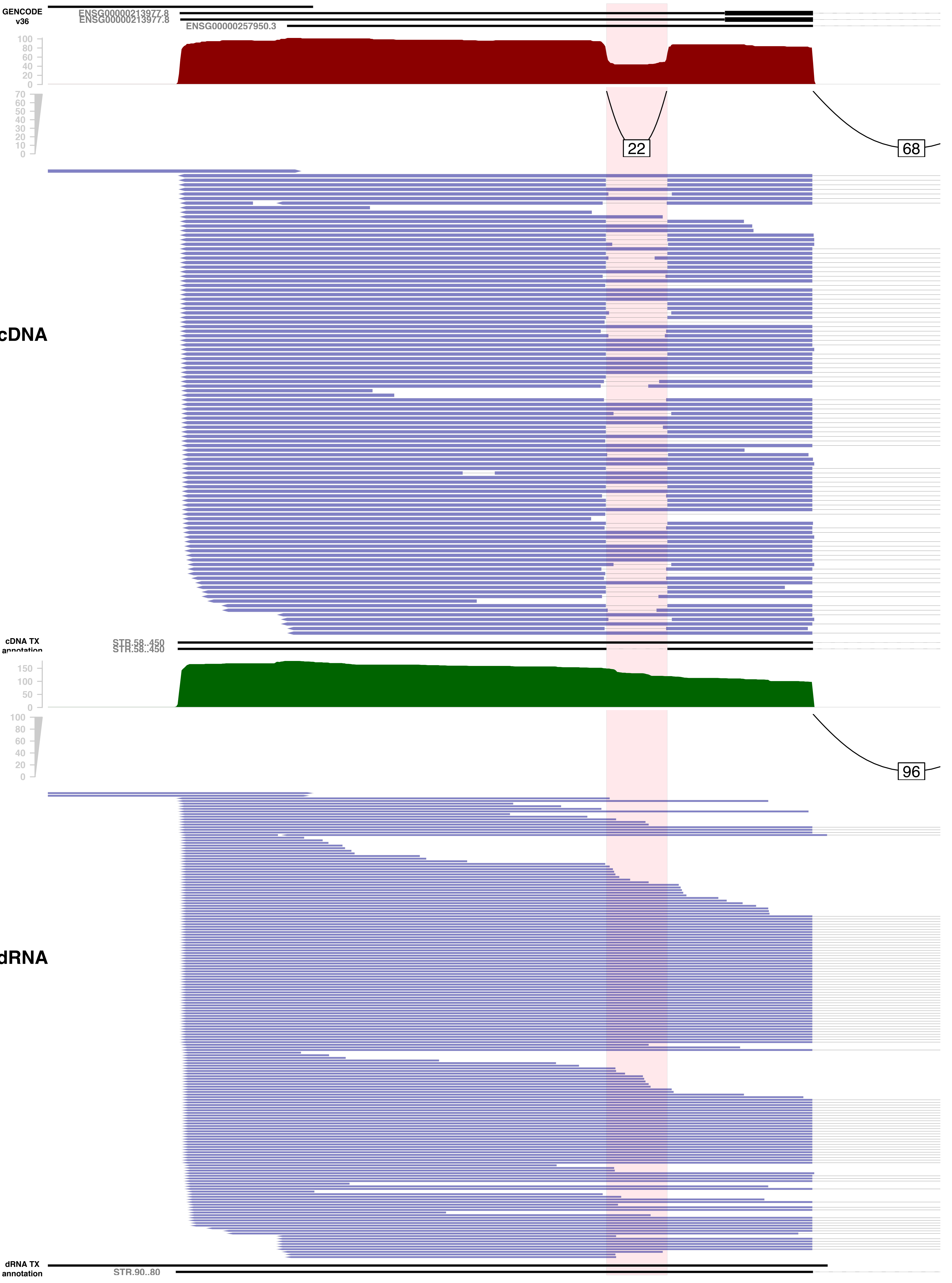

Supplement: Supplementary file 2 — Additional file 2: Data 1. Putative falsitrons in the genomic context. [file 13059_2021_2411_MOESM2_ESM.pdf]
