# Supplementary material for: Direct long-read RNA sequencing identifies a subset of questionable exitrons likely arising from reverse transcription artifacts
Source: Genome Biol. 2021 Jun 28;22:190. doi: 10.1186/s13059-021-02411-1 (PMC8240250; doi:10.1186/s13059-021-02411-1)
Supplement: Supplementary file 5 — Additional file 5. Review history. [file 13059_2021_2411_MOESM5_ESM.docx]

Review History

**First round of review**

**Reviewer 1**

**Are you able to assess all statistics in the manuscript, including the appropriateness of statistical tests used?**

There are no statistics in the manuscript.

**Comments to author:**

This is a truly surprising result, and I was very excited about it. The authors found that small introns are "spliced" out of exons by the RT process during the construction of an RNA-seq library. This creates false introns, and the literature already has reports of many of these "exitrons" in which they are all assumed to be real. Some, perhaps most of them, are not.

I only have one very minor suggestion: the "junction reads" label in figure 1a and 1f doesn't clearly point to the numbers in the small boxes - it took me a while to figure out what those numbers were, and I had to guess. I didn't realize there was a label, "junction reads" until the 4th or 5th time I looked at the figure. I suggest putting the label right next to one of the boxes, with a small arrow pointing to the box, rather than all the way over on the left.

Otherwise this is an excellent paper. I recommend acceptance without any revisions.

**Reviewer 2**

**Are you able to assess all statistics in the manuscript, including the appropriateness of statistical tests used?**

There are no statistics in the manuscript.

**Comments to author:**

The manuscript by Shulz, Torres-Diz et al starts with an analysis of a particular reported exitron (an intron within an exon that preserves the reading frame when retained) in the gene CD19. Upon tracing its origin to a reverse transcription artifact by comparing long-read cDNA and direct RNA sequencing on the Nanopore platform, the authors extend their analysis to identify additional false-positive exitrons ("falsitrons" - I am no fan of this term - please consider changing) as likely RT-artifacts in several other public long-read and short-read cDNA-based datasets. The authors advocate for dRNA sequencing for exitron validation.

Overall, I found the manuscript well-written and interesting to read. While this is quite a short report, I believe that it will be of general interest for those wondering about the benefits of direct RNA sequencing. However, the authors should explicitly discuss the challenges of doing directRNA sequencing given its input amount requirements and low numbers of reads produced (also lower read quality and shorter read lengths).

Given the nanopore-only data and the use of the standard kits, it is difficult to see whether these RT artifacts will apply equally to all cDNA datasets (including PacBio) or only those using particular classes of RT (different RT have different biases related to their "day job" processing specific viral genomes). The authors themselves allude to thermostable RTs potentially overcoming this issue. The authors could expand on this in the results/discussion. A PacBio analysis (using publicly available Sequel 2 data) would be substantial plus for making this universal.

There are a few additional points to address:

-	I was unclear in the methods what annotations the authors are comparing to? Gencode? If so, what version?

-	Why did the authors decide to not use the "—conservative" flag for StringTie? Would StringTie have filtered out this false-positive isoform?

-	I believe that SQANTI originally used a random forest approach to identify RT-artifacts rather than by comparing it to the proteome (the authors also did that in the paper, but the goal was validating any novel isoform). I would fix that part of the discussion to highlight the ML-approach that SQANTI took. I would actually check whether this is still available in modern SQANTI3

**Authors Response**

**Point-by-point responses to the reviewers’ comments:**

Referee 1:

“the "junction reads" label in figure 1a and 1f doesn't clearly point to the numbers in the small boxes - it took me a while to figure out what those numbers were, and I had to guess.”

*We have added a small text box and an arrow to clarify the labeling.*

Referee 2:

1. “the authors should explicitly discuss the challenges of doing directRNA sequencing given its input amount requirements and low numbers of reads produced (also lower read quality and shorter read lengths).”

*A clear statement to that effect has been included in the Conclusions. Regarding the accuracy of dRNA-seq, the official report of Nanopore from May 2021 states that with the new chemistry and algorithms for read calling it reaches above 99% (https://nanoporetech.com/accuracy).*

2. “The authors themselves allude to thermostable RTs potentially overcoming this issue.”

*We have included a statement in the Conclusion mentioning the validity of this approach, for example as implemented in TGIRT-Seq (new Ref. 24).*

3. “A PacBio analysis (using publicly available Sequel 2 data) would be substantial plus for making this universal.”

*As the reviewer had suggested, we expanded our analysis to Iso-Seq (Isoform Sequencing, PacBio) and detected 33 out of 57 falsitrons in the reconstructed isoforms from publicly available Iso-Seq data for several human RNA samples. We also acknowledged in the Conclusions that SQANTI2 correctly filters out the CD19 falsitron but that such filtering could come at the expense of filtering out real exitrons.*

3. “what annotations the authors are comparing to? Gencode? If so, what version?”

*We now state in the Methods that we are using GENCODE gene annotation (v36, genome version hg38)*

4. “why did the authors decide to not use the "—conservative" flag for StringTie? Would StringTie have filtered out this false-positive isoform?”

*The –-conservative flag addresses the issues of coverage and minimum isoform abundance and additionally disables trimming at the end of transcripts. Using our downstream filters, we similarly address the coverage and isoform abundance issues, applying even more stringent requirements on minimum coverage. For our analysis, however, it is important, especially when analyzing dRNA-seq, to have the default trimming at the end of transcripts activated to adjust for coverage drop-outs, like in the case of the 5’ end of CD19.*

5. “I believe that SQANTI originally used a random forest approach to identify RT-artifacts rather than by comparing it to the proteome (the authors also did that in the paper, but the goal was validating any novel isoform). I would fix that part of the discussion to highlight the ML-approach that SQANTI took. I would actually check whether this is still available in modern SQANTI3”

*The reviewer observation is correct, and we have fixed this in our discussion by clarifying the importance of the ML approach implemented in SQANTI. We verified that this approach is still applied in SQANTI3. The official repository specifies that the changes from SQANTI2 to SQANTI3 are essentially the enhanced use of other data types, such as CAGE, to support isoform annotation.*
